# Transcriptional Profiles of Leukocyte Populations Provide a Tool for Interpreting Gene Expression Patterns Associated with High Fat Diet in Mice

**DOI:** 10.1371/journal.pone.0011861

**Published:** 2010-07-29

**Authors:** William R. Swindell, Andrew Johnston, Johann E. Gudjonsson

**Affiliations:** 1 Department of Genetics, Harvard Medical School, Boston, Massachusetts, United States of America; 2 Department of Dermatology, University of Michigan School of Medicine, Ann Arbor, Michigan, United States of America; University of Birmingham, United Kingdom

## Abstract

**Background:**

Microarray experiments in mice have shown that high fat diet can lead to elevated expression of genes that are disproportionately associated with immune functions. These effects of high fat (atherogenic) diet may be due to infiltration of tissues by leukocytes in coordination with inflammatory processes.

**Methodology/Principal Findings:**

The Novartis strain-diet-sex microarray database (GSE10493) was used to evaluate the hepatic effects of high fat diet (4 weeks) in 12 mouse strains and both genders. We develop and apply an algorithm that identifies “signature transcripts” for many different leukocyte populations (e.g., T cells, B cells, macrophages) and uses this information to derive an *in silico* “inflammation profile”. Inflammation profiles highlighted monocytes, macrophages and dendritic cells as key drivers of gene expression patterns associated with high fat diet in liver. In some strains (e.g., NZB/BINJ, B6), we estimate that 50–60% of transcripts elevated by high fat diet might be due to hepatic infiltration by these cell types. Interestingly, DBA mice appeared to exhibit resistance to localized hepatic inflammation associated with atherogenic diet. A common characteristic of infiltrating cell populations was elevated expression of genes encoding components of the toll-like receptor signaling pathway (e.g., *Irf5* and *Myd88*).

**Conclusions/Significance:**

High fat diet promotes infiltration of hepatic tissue by leukocytes, leading to elevated expression of immune-associated transcripts. The intensity of this effect is genetically controlled and sensitive to both strain and gender. The algorithm developed in this paper provides a framework for computational analysis of tissue remodeling processes and can be usefully applied to any *in vivo* setting in which inflammatory processes play a prominent role.

## Introduction

Consumption of a high fat diet is associated with obesity, insulin resistance and the development of chronic health conditions, such as atherosclerosis and Type II diabetes. The rising rates of obesity in some populations have been referred to as an “epidemic”, and there is indication that current trends may worsen in the future [1], [2]. The deleterious health effects associated with obesity have been linked to several metabolic abnormalities, but increasingly, it has been recognized that inflammation is a key process that contributes to negative health outcomes. High fat diets induce a state of heightened inflammation, with elevated levels of C-reactive protein, interleukin-6, and other systemic inflammation markers [3], [4]. Additionally, strong localized inflammation responses to obesity are known to occur within individual tissues. In white adipose tissue, for example, high fat diet can increase macrophage infiltration into fat depots [5], while also promoting infiltration by T-lymphocytes and macrophages [6], [7]. In cardiac tissue, localized recruitment of monocytes is an initiating step in the development of atherosclerotic plaques, which drives development of heart disease in association with high fat diet [8]. In other tissues, including liver, muscle and pancreas, high fat diet can drive excessive accumulation of lipids and their derivatives, leading to a state of lipotoxicity that augments pro-inflammatory signals to promote accumulation and activation of macrophages [9], [10]. High fat diet and obesity are thus associated with systemic inflammation as well as localized inflammatory responses that affect both adipose and non-adipose tissues. This inflammation response may represent a useful target for intervention strategies aimed at combating deleterious health effects of high fat diet and obesity [11]. Inhibitors of the monocyte chemoattractant protein 1 (MCP-1/CCR2) pathway, for instance, have been shown to attenuate insulin resistance and other negative consequences of excessive energy intake in laboratory rodents [12]–[14].

The inflammation response associated with high fat diet is accompanied by shifts in tissue composition, activation of apoptotic pathways, disruption of lipid metabolism, and altered sensitivity to insulin. Recently, the investigation of these complex effects has been facilitated by a large-scale study of hepatic gene expression patterns in male and female mice of 12 mouse strains, in which whole-genome microarrays were used to profile expression in mice that had been maintained on either a high fat (30% fat) or control diet (6% fat) for a period of four weeks [15]. This unique dataset, which includes whole-genome expression patterns from 144 mice, provides a valuable resource for the investigation of gene expression patterns associated with a single dietary intervention. Mechanistic studies of high fat diet in mice have often focused on a single mouse strain, usually C57BL/6J, which is known to exhibit a response to high fat diet that is at least partly idiosyncratic [16]. The data resource provided by Shockley et al. [15] have greatly expanded this focus, providing a tool for identifying aspects of the response to high fat diet that are shared among multiple strains, as well as aspects that are specific to individual strains. An initial analysis of these data highlighted salient features of the hepatic response to high fat diet, including responses that are invariant among all strains and mice, regardless of gender (e.g., increased *Abcg5* expression) [15]. Interestingly, a group of 557 immune-response genes was identified, which were both induced by high fat diet in multiple strains and also correlated with total cholesterol levels [15]. Such genes were disproportionately associated with antigen processing and presentation, and included a number of histocompatibility antigens (e.g., *H2-Ab1*, *H2-Eb1*, *H2-Aa* and *Cd74*). It is possible that, for some immune-associated genes, elevated hepatic expression is a localized response of hepatocytes to damage associated with high fat diet. Alternatively, increased expression of such transcripts in liver may reflect an influx of monocytes, macrophages, T-cells and other white blood cells [17], [18], which express at high levels genes encoding antigens and proteins central to immune processes. This latter possibility suggests that, in mice fed a high fat diet, hepatic gene expression patterns can be used to gauge inflammation intensity, and that targeted analytical methods can be developed to exploit this information to gain insight into the tissue remodeling that accompanies excessive intake of dietary fat.

This study provides a microarray-based characterization of hepatic inflammation responses to high fat diet in male and female mice of 12 different mouse strains. A data-mining algorithm is developed and applied, which leads to the *in silico* calculation of “inflammation profiles” that highlight leukocyte subsets best able to explain gene expression patterns associated with high fat diet. The algorithm utilizes genome-wide expression profiles for leukocyte populations, which are available in public microarray data depositories, to identify sets of “signature transcripts” that represent a molecular fingerprint for particular populations of leukocytes (e.g., CD4+ T cells). For each set of signature transcripts identified, the group-wise response of the set to high fat diet in liver is evaluated, and this response is used to infer whether the associated leukocyte population is likely to infiltrate liver tissue of mice provided a high fat diet. This approach leads to inferences with extremely strong statistical support, and in the present context, generates mechanistic hypotheses useful for building a model of hepatic response to high fat diet, and for understanding how this response is similar and different among mouse strains. The analytical approach developed in this study can also be applied in other *in vivo* settings to better understand inflammation processes on the basis of microarray data.

## Results

### Overview of the transcriptional response to high fat diet in mouse liver

**Figure 1:**
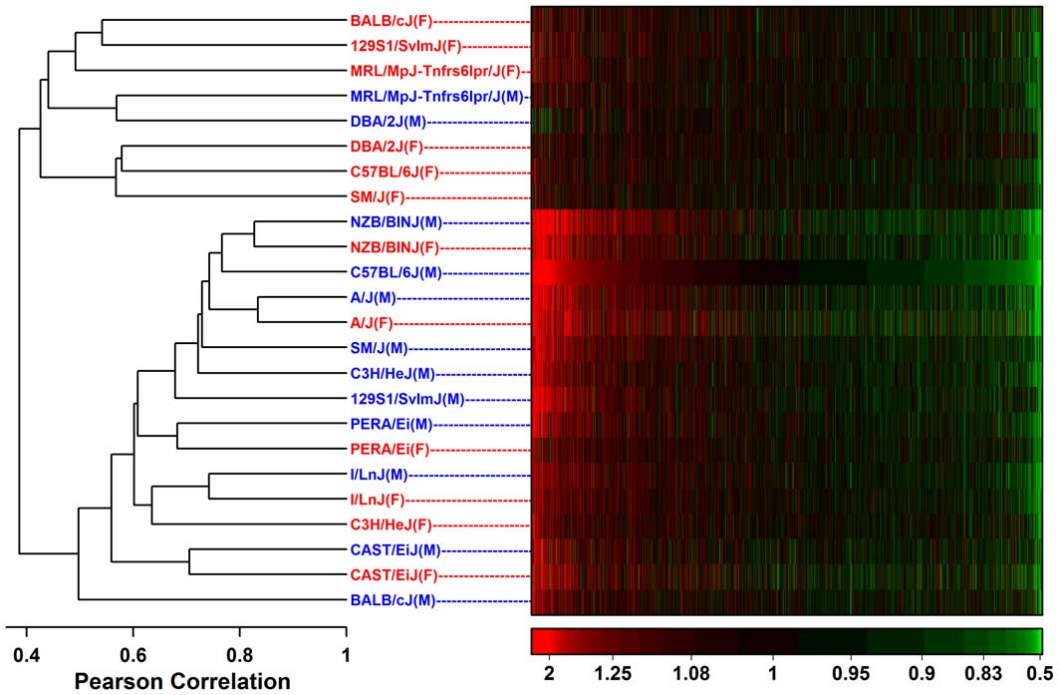
Association among transcriptional responses to high fat diet in male and female mice of 12 mouse strains. The tree diagram was generated from a hierarchical cluster analysis of the 24 strain-gender combinations, where the distance between combinations is based upon the Pearson correlation between fold-change estimates (HF diet/Control) among selected transcripts. The selected transcripts correspond to 17,927 probe sets that were significantly influenced by HF diet (FDR-adjusted P<0.05) with respect to at least one of the 24 strain-gender combinations. In the heatmap, the 17,927 probe sets have been ordered according to the fold-change estimate observed for B6 male mice. The color code used in the heatmap corresponds to the fold-change estimate (HF diet/Control), with red regions representing transcripts elevated by HF diet and green regions representing transcripts decreased by HF diet (see color scale at bottom right). Strain-gender combinations involving male mice are represented by blue labels, while strain-gender combinations involving female mice are represented by red labels.

Microarray data from the Novartis strain-diet-sex survey (GSE10493) was used to evaluate hepatic responses to high fat (HF) diet in male and female mice of 12 mouse strains (129S1/SvImJ, A/J, C57BL/6J, BALB/cJ, C3H/HeJ, CAST/EiJ, DBA/2J, I/LnJ, MRL/MpJ-Tnfrs6lpr/J, NZB/BINJ, PERA/Ei, SM/J) [15]. We compared transcript levels between HF-fed (*n* = 3) and control-fed mice (*n* = 3) for each of 24 strain-gender combinations, where HF-fed mice received 30% Kcal from fat over 4 weeks and control-fed mice received 6% Kcal from fat over the same time period [15]. Response to HF diet varied considerably in magnitude among the 24 strain-gender combinations. In some cases, following FDR adjustment for multiple hypothesis testing, more than 1000 unique genes were either increased or decreased by HF diet (e.g., NZB males and females, A/J females; see Table 1). In other cases, fewer than 50 unique genes were increased or decreased by HF diet (e.g., females of the SM, DBA, B6 and 129 strains; see Table 1). Cluster analysis identified two groups among the strain-gender combinations, with one group exhibiting lower response to HF diet (mostly females; see Figure 1), and the other group exhibiting a heightened response to HF diet (mostly males; see Figure 1). In some cases, the “strain effect” appeared to dominate the “gender effect”, and males and females of the same strain clustered together (e.g., see NZB, A, PERA, I, CAST in Figure 1), although this was not true for some strains (e.g., see females from the BALB, 129 and MRL strains in Figure 1).

**Table 1:** Effect of high fat diet on gene expression in male and female mice of 12 mouse strains.

Strain (Gender)	Increased	Decreased	Increased†	Decreased†	% Increased
129S1/SvImJ (F)	2351 (1699)	1510 (1183)	64 (54)	20 (17)	47.85
129S1/SvImJ (M)	3291 (2380)	2811 (2216)	627 (505)	70 (62)	39.56
A/J (F)	6210 (4171)	6018 (4356)	3315 (2346)	1646 (1234)	39.71
A/J (M)	4825 (3178)	5155 (3920)	1164 (862)	508 (393)	40.55
C57BL/6J (F)	1369 (1037)	1733 (1439)	37 (34)	45 (36)	47.15
C57BL/6J (M)	3284 (2408)	3203 (2316)	391 (328)	239 (205)	40.81
BALB/cJ (F)	3163 (2335)	2637 (1902)	461 (367)	223 (176)	47.18
BALB/cJ (M)	2622 (1944)	2730 (2239)	145 (125)	26 (24)	43.82
C3H/HeJ (F)	3511 (2711)	2414 (1830)	493 (397)	174 (134)	46.95
C3H/HeJ (M)	3393 (2459)	3317 (2409)	1228 (960)	745 (551)	45.1
CAST/EiJ (F)	4359 (2988)	4236 (3237)	1336 (1010)	566 (451)	42.15
CAST/EiJ (M)	2739 (1985)	2120 (1623)	660 (520)	133 (110)	43.21
DBA/2J (F)	2298 (1787)	1777 (1390)	24 (21)	13 (13)	47.08
DBA/2J (M)	2028 (1534)	1792 (1315)	202 (144)	123 (94)	50.15
I/LnJ (F)	2999 (2251)	2043 (1544)	547 (444)	208 (152)	45.31
I/LnJ (M)	2815 (2054)	2161 (1698)	241 (199)	152 (114)	43.84
MRL/MpJ-Tnfrs6lpr/J (F)	2953 (2285)	2897 (2050)	354 (287)	235 (179)	49.1
MRL/MpJ-Tnfrs6lpr/J (M)	2163 (1663)	2347 (1729)	207 (161)	239 (175)	51.63
NZB/BINJ (F)	4900 (3439)	4949 (3418)	2349 (1751)	1743 (1264)	41.65
NZB/BINJ (M)	5329 (3513)	6515 (4861)	2512 (1799)	1249 (991)	35.71
PERA/Ei (F)	3180 (2353)	2422 (1919)	451 (370)	102 (89)	44.32
PERA/Ei (M)	3600 (2671)	2849 (2116)	912 (723)	295 (238)	42.97
SM/J (F)	1297 (1020)	1156 (915)	19 (16)	27 (23)	49.6
SM/J (M)	5066 (3925)	4659 (3150)	477 (394)	1101 (818)	59.73

† Number of significantly increased and decreased transcripts is based upon p-values that have been adjusted using the Benjamini-Hochberg method to control the false discovery rate.

For each strain and gender combination, the table lists the number of probe sets increased and decreased significantly for each comparison between mice maintained on a high fat diet (*n* = 3) and mice maintained on a control diet (*n* = 3). The listed values correspond to the number of significantly altered probe sets (P<0.05). The number of unique mouse genes associated with the significantly altered probe sets is given in parentheses. The fourth and fifth columns list the number of significantly altered probe sets and transcripts, based upon p-values that have been adjusted using the Benjamini-Hochberg approach for controlling the false discovery rate (i.e., FDR-adjusted P<0.05). The final column lists the global percentage of probe sets increased by high fat diet (including all probe sets, both significant and non-significant).

Gene ontology analyses revealed that biological processes associated with immune responses (e.g., antigen processing and presentation, defense response to Gram-positive bacteria, activated T cell proliferation, chemotaxis and cell adhesion) were the most frequently overrepresented gene ontology terms among the genes increased by HF diet (Figures S1 and S2). For instance, in males, genes increased by HF diet were disproportionately associated with antigen processing and presentation in 9 of the 12 mouse strains (Figure S1). Among females, the trend was less strong, and genes increased by HF diet were disproportionately associated with antigen processing and presentation in 5 of 12 mouse strains, although other frequently overrepresented processes included neutrophil chemotaxis (7 of 12 strains) and inflammatory response (7 of 12 strains) (Figure S2). These results are in agreement with those presented by Shockley et al. [15], and suggest that increased expression of genes involved in immune system processes is a major feature of the hepatic response to HF diet, which occurs robustly in male and female mice and among multiple mouse strains.

### An *in silico* hepatic inflammation profile associated with high fat diet in B6 Males

**Figure 2:**
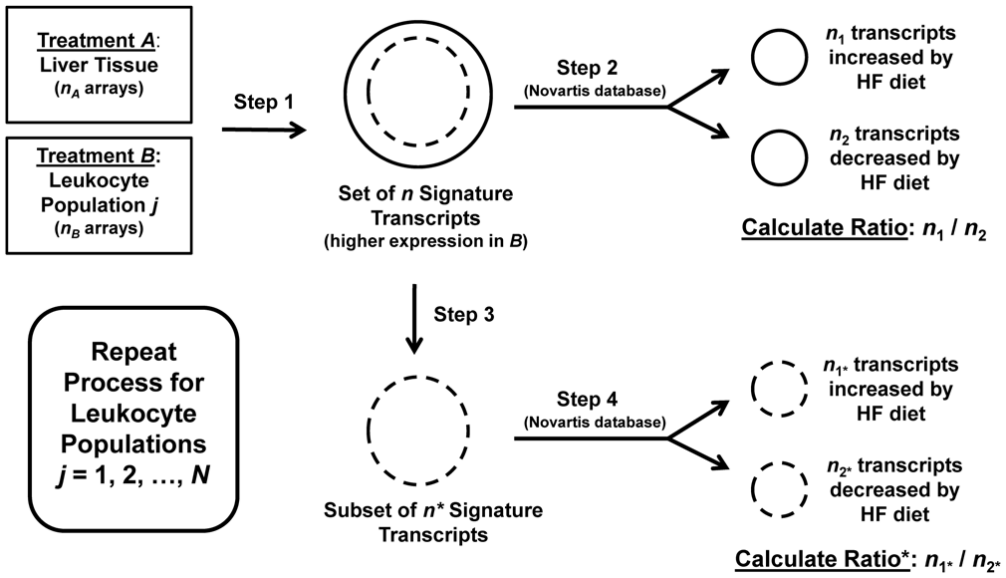
Procedure for calculation of inflammation profiles. The figure illustrates the computational procedure used to calculate inflammation profiles for each of the 24 strain-gender combinations evaluated in this study (see Methods). A total of *N* = 200 leukocyte populations were evaluated for each of the 24 strain-gender combinations. For a given leukocyte population *j*, a set of *n* signature transcripts are identified based upon a two-sample comparison between treatments *A* and *B*. Treatment *A* consists of *n_A_* arrays that have been used to evaluate gene expression in liver tissue of young mice maintained on a normal diet (Table S1). Treatment *B* consists of *n_B_* arrays used to evaluate RNA extracted from the *j*th leukocyte population (Table S2). The *n* signature transcripts identified from this comparison represent those transcripts for which expression is significantly higher in treatment *B* relative to treatment *A* (FDR-adjusted P-value<10^−4^ and fold-change≥16). In step 2, the *n* signature transcripts are divided into the *n*_1_ transcripts increased by high fat diet and the *n*_2_ transcripts decreased by high fat diet, and the ratio between *n*_1_ and *n*_2_ is calculated. The ratio *n*_1_/*n*_2_ serves as a score for the *j*th leukocyte population and provides an indication of whether signature transcripts associated with population *j* are disproportionately elevated in hepatic tissue of mice provided a high fat diet. Steps 3 and 4 lead to the calculation of a second ratio (Ratio* = *n*_1*_/*n*_2*_), which is used as secondary statistical significance criteria for each population (see Methods). In step 3, the *n* signature transcripts are reduced to a subset of n* transcripts. This filtering step excludes transcripts that had been designated as signature transcripts for any of the other *j* - 1 leukocyte populations that, in step 2, had been assigned a higher score (i.e., a higher *n*_1_/*n*_2_ ratio; see Methods). In step 4, the ratio *n*_1*_/*n*_2*_ is calculated based on the n* signature transcripts, in the same fashion as described in step 2.

We hypothesized that, among transcripts elevated by HF diet, over-abundance of transcripts related to immune system processes was at least partly due to an inflammatory response, in which circulating leukocyte populations infiltrate hepatic tissue of mice provided a HF diet, thereby driving increased expression of genes that exhibit high expression in leukocytes. To evaluate this possibility, we developed a data mining procedure, which searches among gene expression profiles of leukocyte populations and their subsets, identifies signature transcripts for each population, and then evaluates whether such signature transcripts are disproportionately elevated in hepatic tissue of mice provided a HF diet (Figure 2). The approach leads to the generation of an *in silico* “inflammation profile” associated with response to HF diet, which provides indication of the degree to which effects of HF diet may be attributable to leukocyte infiltration, and also points to particular cell populations that are likely to be prominent components of the inflammatory infiltrate.

**Figure 3:**
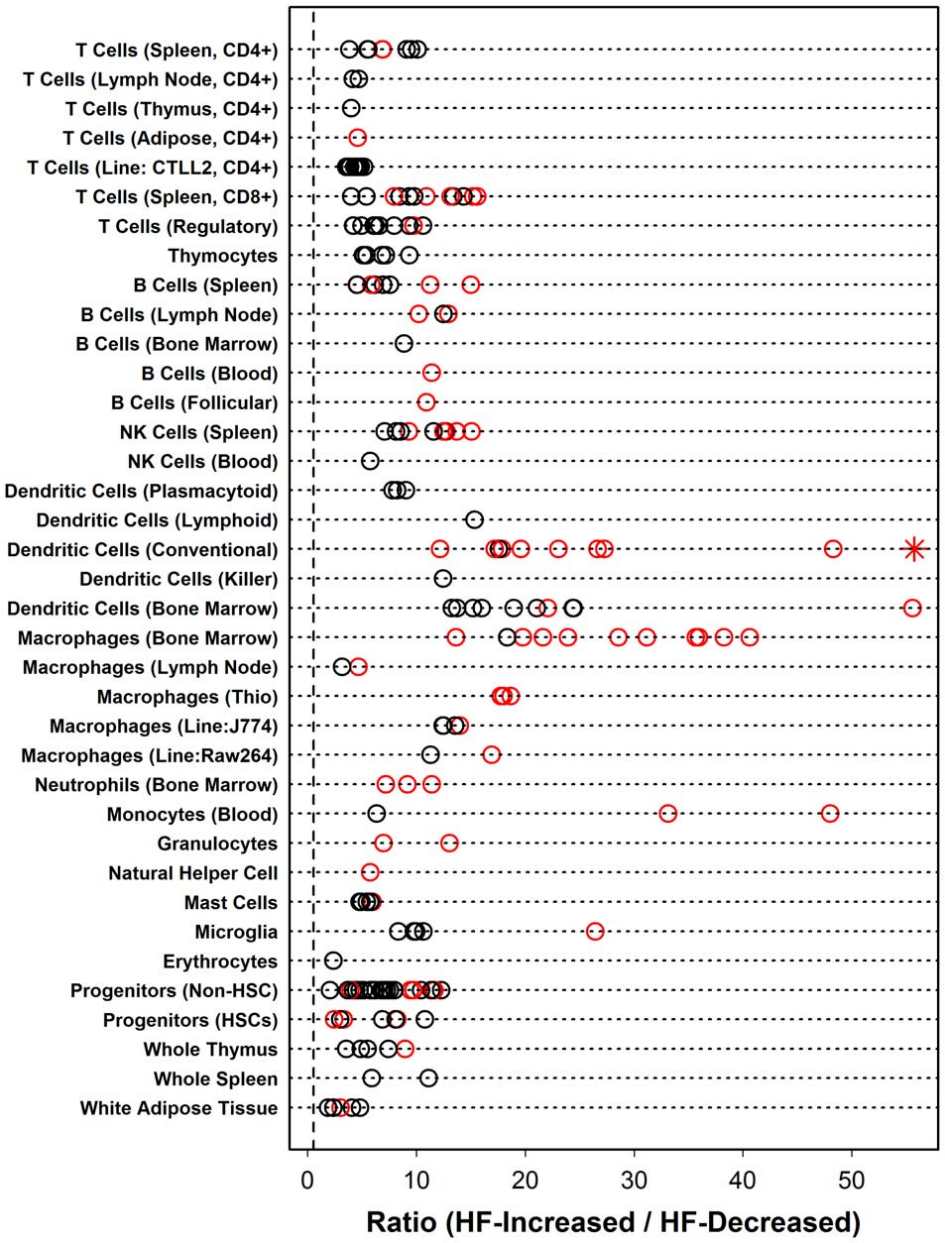
Inflammation profile highlights infiltration of liver tissue by dendritic cells, macrophages and monocytes in C57BL/6J male mice provided a high fat (HF) diet. For each of *N* leukocyte populations, signature transcripts were identified, and the ratio of HF-increased to HF-decreased transcripts among these signature transcripts was determined (see Figure 2). Each symbol represents one of the *N* = 200 populations evaluated, which have been categorized according to labels listed in the left margin. The placement of each symbol along the horizontal axis corresponds to the ratio between the number of HF-increased and HF-decreased transcripts (among the *n* signature transcripts associated with a given population; see Figure 2). The dotted vertical line indicates the ratio of HF-increased to HF-decreased transcripts among all probe sets represented on the Affymetrix 430 2.0 Mouse Genome Array. Black symbols represent populations for which significance criteria were not satisfied, while red symbols correspond to populations for which all significance criteria was satisfied (see Methods). The highest-scoring population is represented by an asterisk symbol (*) (CD8A- myeloid dendritic cells). The figure corresponds to the inflammation profile calculated for C57BL/6J male mice provided a high fat diet. Profiles calculated for each of the 23 other strain-gender combinations are included as Figure S3.

**Figure 4:**
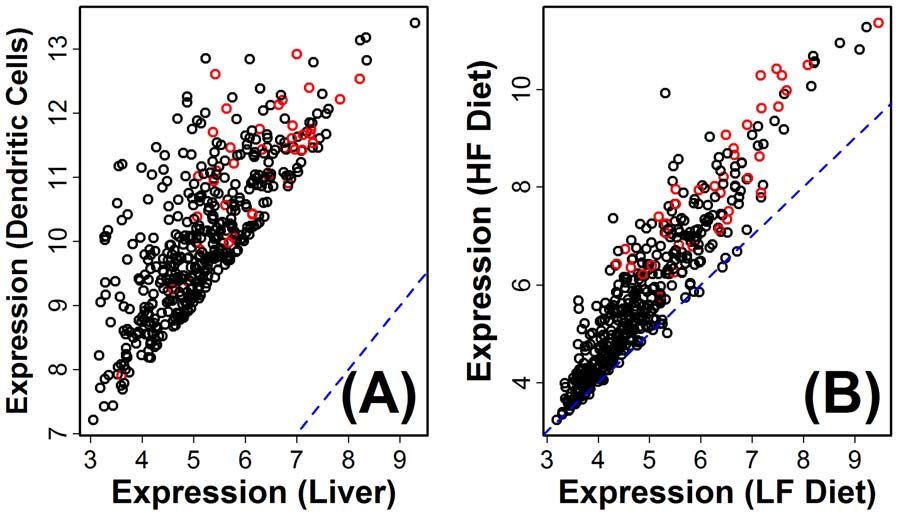
High fat diet disproportionately increases expression of dendritic cell-associated transcripts in liver tissue from C57BL/6J male mice. The inflammation profile calculated for C57BL/6J male mice (Figure 3) identified 454 transcripts associated with CD8A- myeloid dendritic cells as disproportionately elevated within hepatic tissue of B6 males provided a high fat diet (see Gene Expression Omnibus samples GSM258647 and GSM258648). In part (A), each symbol represents one of these 454 transcripts, and the axes correspond to the average expression level in mouse liver tissue (i.e., treatment *A* in Figure 2) or CD8A- myeloid dendritic cells (i.e., treatment *B* in Figure 2). In part (B), the 454 transcripts are plotted with respect to their average expression level in hepatic tissue among B6 mice provided a high fat diet (vertical axis) and B6 mice provided a low fat diet (horizontal axis). In both (A) and (B), red symbols correspond to transcripts that were significantly increased by high fat diet (FDR-adjusted P<0.05), and the dashed blue line represents equal expression with respect to the vertical and horizontal axes.

**Figure 5:**
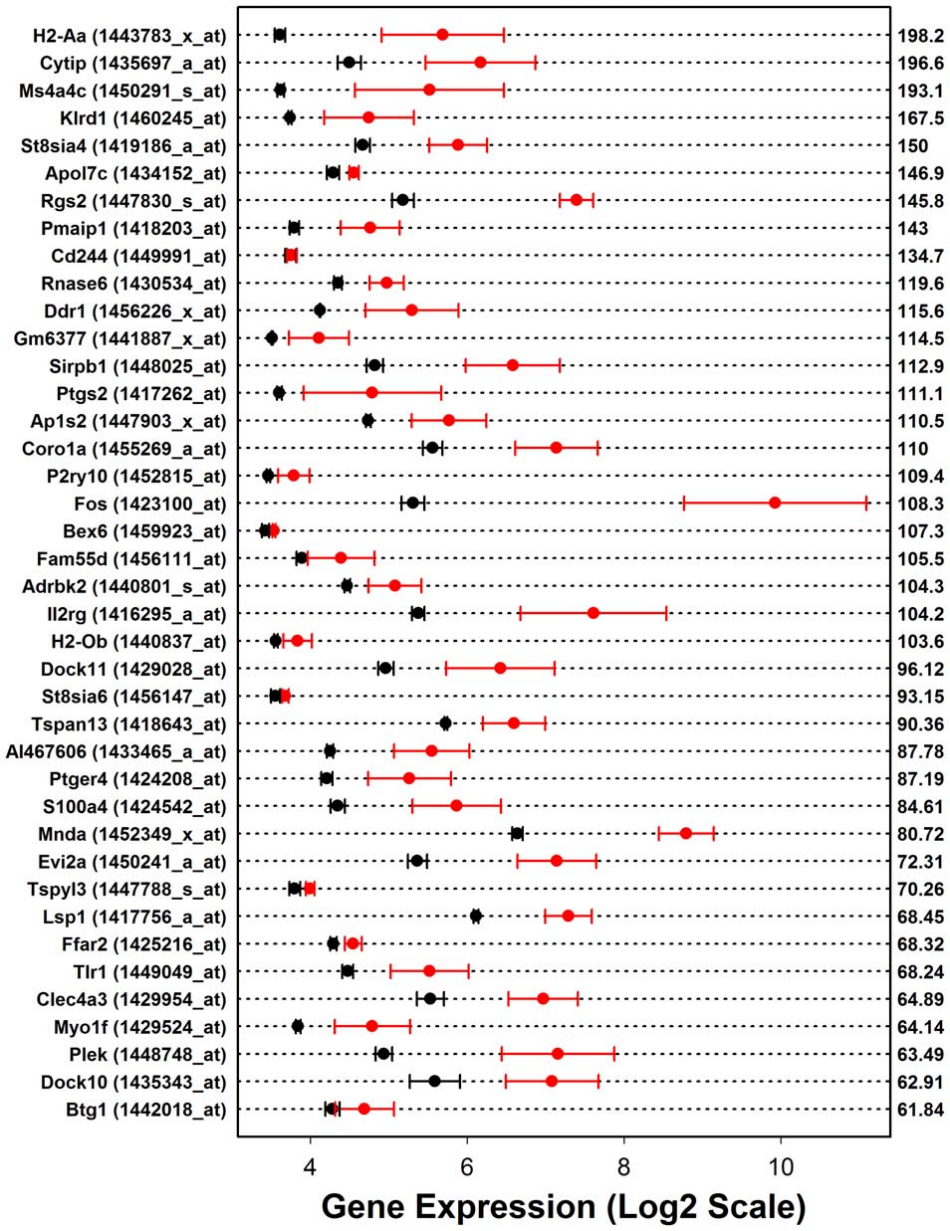
High fat diet increases hepatic expression for each of the 40 transcripts most highly expressed in dendritic cells relative to liver tissue (C57BL/6J males). The inflammation profile calculated for C57BL/6J male mice (Figure 3) identified 454 transcripts associated with CD8A- myeloid dendritic cells as disproportionately elevated within hepatic tissue of B6 males provided a high fat diet (see Gene Expression Omnibus samples GSM258647 and GSM258648). The chart lists the top 40 signature transcripts that were associated with this dendritic cell population. These transcripts have been ordered according to values listed on the right side of the chart, which represent the expression ratio between the dendritic cell population and liver tissue (i.e., ratio between average expression of treatments *B* and *A* in Figure 2). Within the chart, red symbols indicate the average expression of each transcript among male B6 mice provided a high fat diet (*n* = 3), while black symbols represent average expression of control mice provided a standard laboratory diet (*n* = 3). The error bars associated with each average expression estimate spans ± one standard error.

The method was first applied to calculate an inflammation profile associated with response to HF diet in B6 male mice, which is perhaps the most commonly investigated strain and gender in physiological studies of excessive dietary fat intake (Figure 3). This revealed that, in B6 males, signature transcripts associated with many leukocyte populations are disproportionately elevated in hepatic tissue of B6 males provided a HF diet, including CD4+ and CD8+ T cells, B cells, NK cells, dendritic cells (DCs), macrophages (Mφ), neutrophils, monocytes, granulocytes and natural helper cells (Figure 3). Among all cell types, however, those from the monocyte- Mφ and monocyte-DC lineages scored most highly (Figure 3). For instance, the strongest trend was present among signature transcripts associated with a population of CD8A- myeloid dendritic cells (Gene Expression Omnibus samples GSM258647 and GSM258648). With respect to this population, the procedure identified a total of 454 signature transcripts, and nearly all of these were elevated in mice provided a HF diet relative to mice provided a control diet (Figure 4). In particular, of the 454 signature transcripts, 446 were increased by HF diet while only 8 were decreased (ratio = 446/8 = 55.7; FDR-adjusted P = 7.15×10^−161^). Moreover, 37 of the 454 signature transcripts were increased significantly (FDR-adjusted P<0.05), while none were significantly decreased (FDR-adjusted P = 7.71×10^−24^). Transcripts most strongly associated with this dendritic cell population included *H2-Aa*, *Cytip* and *Ms4a4c*, and each of these transcripts was elevated in hepatic tissue of mice provided a HF diet (Figure 5). These results provided strong indication that, as a group, DC-associated transcripts are disproportionately elevated in liver of B6 males provided a HF diet, consistent with the hypothesis that leukocyte populations, particularly DCs, infiltrate hepatic tissue of B6 males as part of an inflammatory response that accompanies excessive fat intake.

### *In silico* hepatic inflammation profiles associated with high fat diet in male and female mice of 12 mouse strains

**Figure 6:**
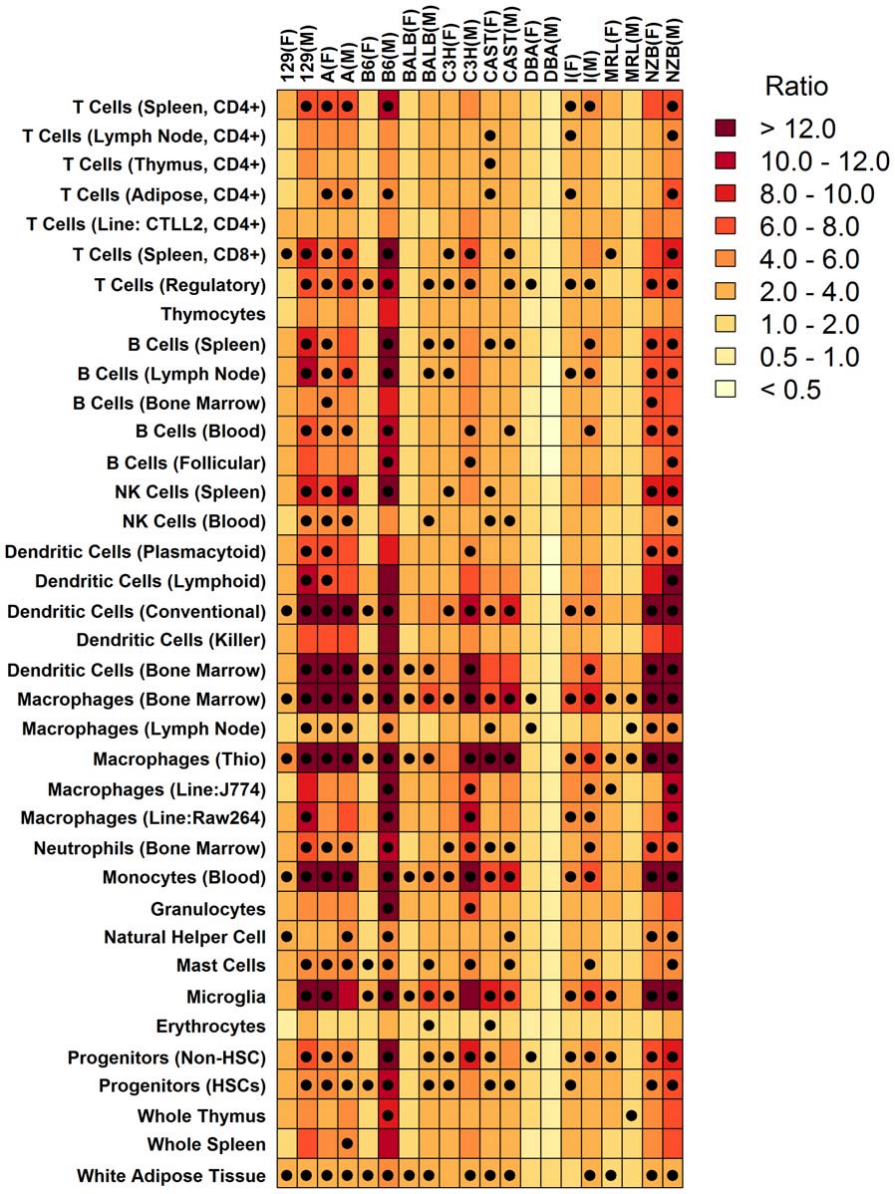
Summary of inflammation profiles calculated for each of 12 mouse strains and both genders. This figure provides a summary of the inflammation profiles calculated with respect to each of 24 strain-gender combinations (see Figure 3 and Figure S3). The *N* = 200 cell populations evaluated by our analysis were categorized into classes listed on the side of the chart. For each strain-gender combination (columns), the largest ratio (HF-increased/HF-decreased; see Figure 2) among cell populations associated with a given class was identified. The color-code in the chart corresponds to this largest ratio (see legend), where darker colors provide indication that a cell population associated with the row label infiltrates hepatic tissue with HF diet. Black dots indicate that, for a given strain-gender combination and class of cell populations, there was at least one population for which statistical significance criteria was satisfied (i.e., see red symbols in Figure 3; see Methods for explanation of statistical significance criteria).

The above analyses focus on B6 male mice, but the most interesting aspect of the Novartis strain-diet-sex survey data is the possibility of examining how patterns compare across a diversity of mouse strains and between both genders. We thus calculated a complete inflammation profile for each of the other 23 strain-gender combinations (Figure S3). A summary of all inflammation profiles is provided in Figure 6. This analysis revealed a number of consistencies among the strain-gender combinations, as well as certain aberrant patterns.

The most consistent pattern associated with HF diet was increased expression of transcripts associated with bone-marrow derived Mφ (Figure 6). This effect was detected with respect to all strain-gender combinations, with the exception of DBA males (see below). Aside from bone-marrow derived Mφ, HF diet also increased transcripts associated with thioglycollate-elicited peritoneal Mφ, conventional DCs, regulatory T cells and white adipose tissue (Figure 6). There was strong correspondence between the magnitude of inflammation profile ratios (HF-increased/HF-decreased) and the clustering patterns observed in Figure 1. In particular, ratios were especially large for NZB mice (males and females), A/J mice (males and females), 129 males and B6 males, and in Figure 1, these strain-gender combinations clustered together in a single group. This result suggests that the genome-wide response to HF diet is correspondent with, and perhaps partly dependent upon, the leukocyte-infiltration signatures detected by inflammation profiles.

**Figure 7:**
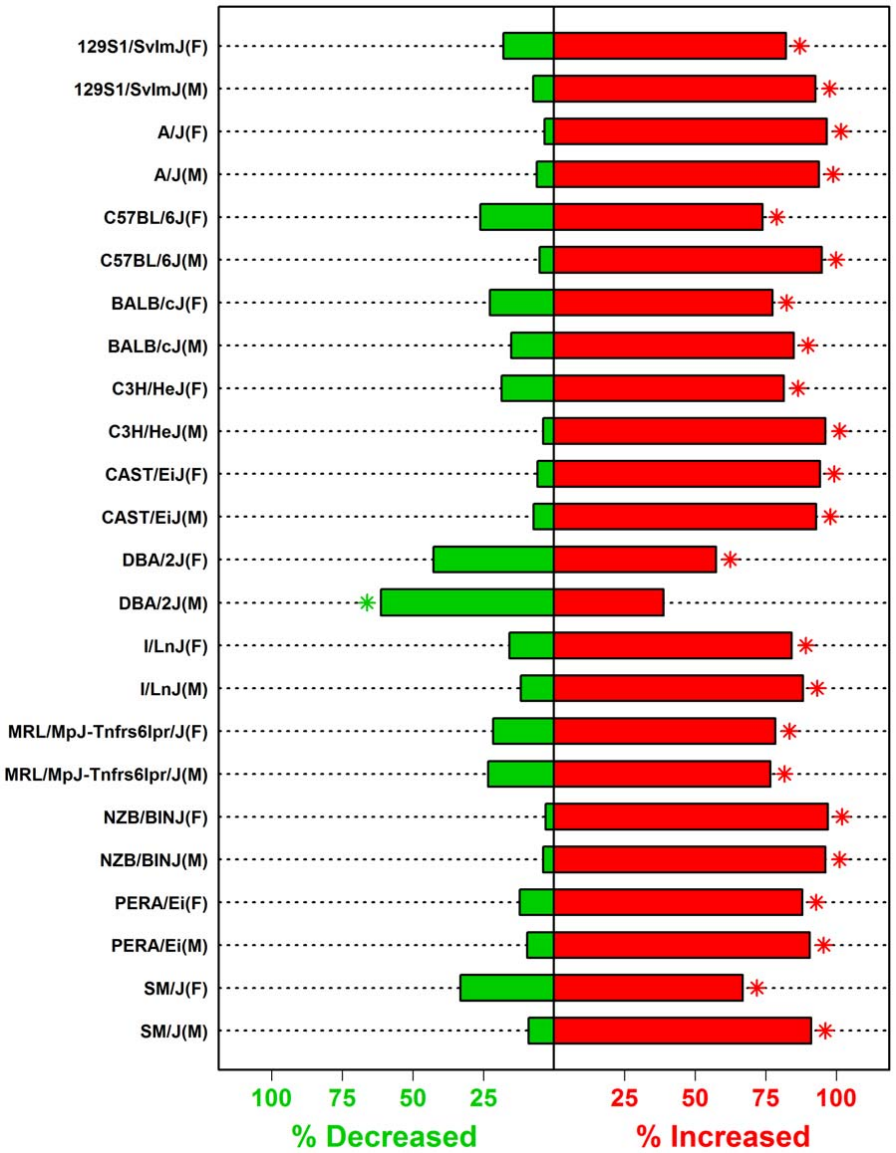
Effect of high fat (HF) diet on expression of macrophage-associated transcripts in male and female mice of 12 strains. Inflammation profiles calculated for each of the 24 strain-gender combinations (Figure 3 and Figure S3) revealed that 570 signature transcripts associated with thioglycollate-elicited peritoneal Mφ (Gene Expression Omnibus samples 258701 and 258702) were most consistently elevated by HF diet in all strain-gender combinations. For each strain-gender combination, the bar graph indicates the percentage of the 570 Mφ-associated transcripts that increased or decreased in hepatic tissue of mice provided with a high fat diet. The asterisk symbol (*) indicates that the percentage of increased or decreased transcripts is significantly large or small (P<0.05; hypergeometric test).

The single cell population that, across all strains and genders, consistently scored most highly on inflammation profiles was a population of thioglycollate-elicited peritoneal Mφ (Gene Expression Omnibus samples 258701 and 258702) (Figure 7). For this Mφ population, 570 signature transcripts were identified (e.g., *Mmp12*, *Gpnmb*, *Bex1* and *Il7r*), and among the 24 strain-gender combinations, HF diet usually increased 70–90% of these signature transcripts in liver (Figure 7). For 23 of 24 strain-gender combinations, the proportion of the 570 transcripts increased by HF diet was statistically significant (Figure 7). An extreme case was NZB/BINJ females, for which 553 of the 570 signature transcripts (97.01%) were increased by HF diet, with 406 signature transcripts exhibiting a statistically significant increase (FDR-adjusted P<0.05). At the other end of the spectrum, transcripts associated with thioglycollate-elicited peritoneal Mφ were not strongly elevated by HF diet in DBA mice (Figure 7). In DBA females, only 57.37% of signature transcripts (327 of 570) were elevated by HF diet. In DBA males, however, 61.2% of signature transcripts (349 of 570) were *decreased* by HF diet, and this proportion of HF-*decreased* transcripts was statistically significant (FDR-adjusted P = 1.57×10^−8^; Figure 7).

There was considerable variation in terms of the degree to which inflammation profile results correlated between males and females of the same mouse strain (Figure S4). For some strains, leukocyte populations that scored highly in males and also tended to score highly in females, with an overall correlation between inflammation profile ratios that exceeded 0.90 (e.g., strains A, C3H, I and K; Figure S4). On the other hand, for the DBA and SM mouse strains, the correlation between males and females was relatively low (0.16 and 0.36, respectively), suggesting potential gender differences in the degree and type of hepatic inflammation that develops in response to HF diet (Figure S4).

### DBA mice exhibit resistance to inflammatory gene expression patterns associated with high fat diet

**Figure 8:**
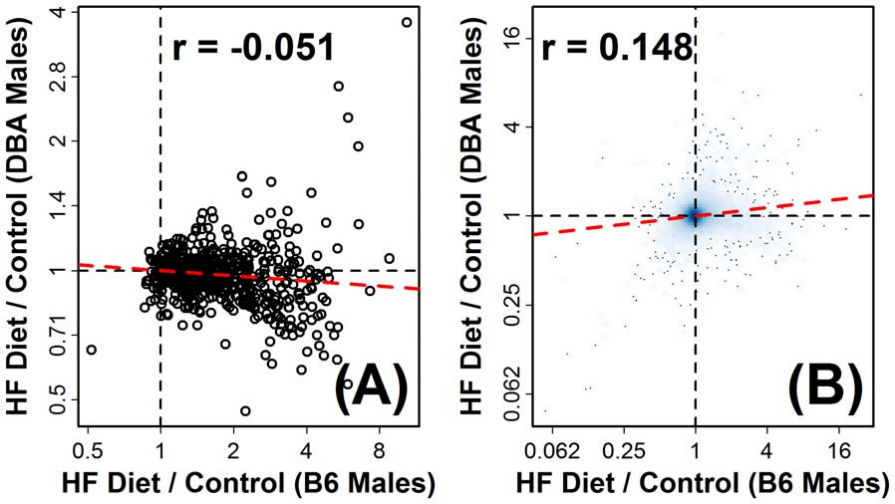
Macrophage-associated transcripts exhibit disparate responses to high fat diet in C57BL/6J relative to DBA/2J male mice. A population of thioglycollate-elicited peritoneal Mφ was identified, on the basis of inflammation profiles (Figures 3 and S3), as the most consistently high-scoring population among the 24 strain-gender combinations. The 570 signature transcripts associated with this population were disproportionately elevated by HF diet in most strains, with the exception of DBA male mice (see Figure 7). In part (A), the effects of high fat diet on expression of these 570 transcripts in C57BL/6J male mice (horizontal axis) is shown relative to the effects of high fat diet in DBA/2J male mice (vertical axis). Note that both the horizontal and vertical axis correspond to log_2_ scales. The dotted red line represents a least-squares regression estimate, and the correlation (*r*) value displayed in the figure is the Pearson correlation between fold-change estimates (HF Diet/Control) from the two mouse strains. In part (B), the same relationship between fold-change estimates is shown, except the figure is based on all 45,101 probe sets represented on the Affymetrix 430 2.0 array, where the density of shading indicates the number of probe sets associated with a given region in the plotting area.

The DBA mice were aberrant relative to other mouse strains and, based upon inflammation profiles, there was little or no indication that HF diet strongly elevated expression of leukocyte-associated transcripts. With regard to the 570 transcripts associated with thioglycollate-elicited peritoneal Mφ (see above; Figure 7), there was no correlation between DBA and B6 males in terms of how these transcripts responded to HF diet (*r* = −0.051; see Figure 8). A close inspection of inflammation profiles calculated for DBA mice reveals further aspects of the strain that are unique (Figure S3). In DBA females, ratios of HF-increased to HF-decreased signature transcripts were much lower relative to all other strain-gender combinations (HF-increased/HF-decreased ratio<2; Figure S3). In DBA males, trends were especially striking, with leukocyte-associated transcripts exhibiting a response to HF diet that was opposite to that in other mouse strains (Figure S3). For instance, 282 signature transcripts were identified with respect to one CD8+ T cell population, and 208 (73.8%) of these transcripts were decreased in hepatic tissue of DBA males provided the HF diet (FDR-adjusted P = 4.15×10^−15^; Figure S3). These results suggest that DBA mice, and particularly DBA males, exhibit resistance to the elevated expression of leukocyte-associated transcripts with HF diet that is characteristic of other mouse strains included in our analysis.

### The gene expression phenotype of high-scoring cell populations: Toll-like receptor signaling as a potential mediator of hepatic infiltration

**Figure 9:**
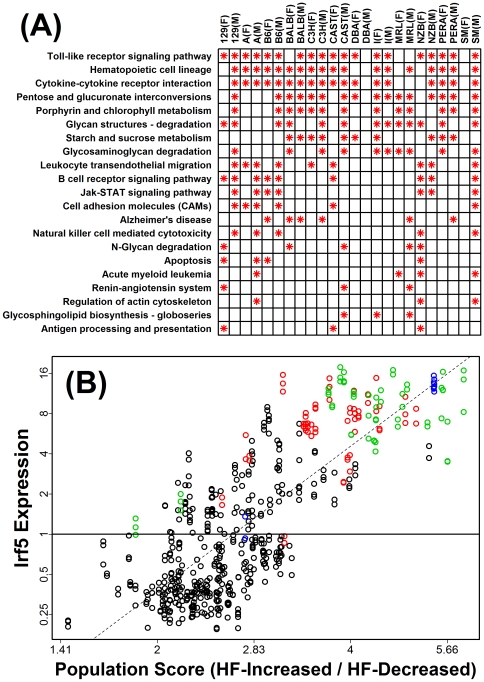
Shared characteristics of gene expression phenotypes associated with infiltrating cell populations. It was hypothesized that high-scoring cell populations may share common characteristics (e.g., elevated expression of genes that facilitate homing to liver or attachment to endothelial surfaces). All transcripts were therefore evaluated to determine if their expression in leukocyte populations correlated with the scores assigned to populations based upon inflammation profiles (i.e., HF-increased/HF-decreased ratios; see Figures 3 and Figure S3). The analysis was repeated for each strain-gender combination, and in each case, transcripts were ranked to identify the 200 transcripts for which expression most strongly increased among cell populations assigned high scores on the inflammation profile associated with each strain-gender combination. In part (A), a summary of all results in shown, based upon KEGG pathway terms. For the 200 transcripts identified with respect to each strain-gender combination, significantly overrepresented KEGG terms were identified (P<0.05). In the chart, a red asterisk symbol indicates that the corresponding KEGG term (rows) was overrepresented with respect to analyses performed for a given strain-gender combination (columns). Part (B) shows an example of a transcript (*Irf5*) for which expression levels were generally elevated among cell populations assigned high inflammation profile scores (C3H female mice; Figure S3). Each point corresponds to a data sample (i.e., array hybridization) associated with one of the 200 leukocyte populations evaluated (Table S2). The horizontal axis indicates the inflammation profile score for the population associated with each data sample (i.e., HF-increased/HF-decreased ratios; C3H female mice; see Figure S3; log_2_ scale). The vertical axis corresponds to *Irf5* expression (log_2_ scale). Red, green and blue points represent data samples associated with DCs, Mφ and monocytes, respectively. The dashed line corresponds to a least squares regression estimate (P = 9.1×10^−118^).

The ability of a cell population to infiltrate hepatic tissue may depend upon expression of key receptors that facilitate homing to liver, attachment to endothelial surface, or transendothelial migration into the tissue [19]. We therefore characterized the gene expression phenotype of high-scoring cell populations to identify potential unifying characteristics (Figure 9). Populations with high scores on inflammation profiles were more likely to highly express transcripts associated with the toll-like receptor (TLR) signaling pathway, degradation of glycan structures, leukocyte transendothelial migration, Jak-STAT signaling, cytokine-cytokine receptor interaction, and cell adhesion molecules (Figure 9A). The most robust characteristic of high-scoring cell populations was elevated expression of genes associated with TLR signaling (e.g., *Tlr2*, *Tlr13*, *Irf5*, *Myd88*, *Il10rb*). For 19 of the 24 strain-gender combinations that we evaluated, transcripts associated with TLR signaling were overrepresented among the 200 transcripts for which expression was most strongly correlated with population scores on inflammation profiles (Figure 9A). The *Irf5* gene, for example, encodes a transcription factor (interferon regulatory factor 5) that is activated downstream of the TLR-Myd88 signaling pathway [20]. Our analyses revealed that, with respect to C3H females as well as males of the 129, B6 and I strains, expression of *Irf5* in leukocyte populations was a better predictor of inflammation profile score than any other single transcript (Figure 9B). This trend was, in part, attributable to the high expression of *Irf5* in cell types usually assigned high scores on inflammation profiles (e.g., Mφ, DCs and monocytes; see Figure 9B). Nevertheless, there were some Mφ and monocyte populations with low inflammation profile scores, and in these populations, *Irf5* expression was comparatively low (Figure 9B). Likewise, among other high-scoring populations, apart from those associated with the monocyte-Mφ and monocyte-DC lineages, expression of *Irf5* expression was comparatively high (Figure 9B). These results indicate that high expression of *Irf5* (and other genes associated with TLR signaling) is a common characteristic of cell populations that appear to infiltrate hepatic tissue in mice provided a HF diet.

### Expression of macrophage-associated transcripts is correlated with total cholesterol and other phenotypic characteristics

We hypothesized that leukocyte-associated transcripts associated with high scoring populations from inflammation profiles may be linked to certain phenotypic characteristics that are sensitive to HF diet (e.g., cholesterol, triglycerides, glucose). For instance, as shown in Figure 5, the transcript *H2-Aa* was a signature transcript of CD8A- myeloid dendritic cells, and previously, Shockley et al. [15] reported that this and other antigen-associated transcripts exhibited a strong correlation with total cholesterol levels among 120 mice evaluated in the Novartis strain-diet-sex study. To determine if this was a general characteristic of leukocyte-associated transcripts, we focused on the 570 signature transcripts associated with the consistently high-scoring population of thioglycollate-elicited peritoneal Mφ (see Figure 7), and evaluated whether these transcripts tended to have a strong correlation with total cholesterol levels. As predicted, there was an unusually large correlation between the expression of these 570 signature transcripts and total cholesterol levels (Figure S5). On average among the 570 transcripts, the correlation with total cholesterol was 0.556, which was significantly larger than correlations observed for other probe sets represented on the array (*r* = −0.079; P = 7.71×10^−291^ based on t-test comparison; see Figure S5). Correlations were especially strong for those transcripts that were most increased by HF diet (Figure S5). We next evaluated average correlations among the 570 transcripts with respect to other phenotypic characteristics (mean *r* = 0.349, high density lipoprotein; mean *r* = 0.314, glutamate dehydrogenase; mean *r* = −0.280, triglycerides; mean *r* = −0.200, glucose; mean *r* = −0.173, nonesterified fatty acids; mean *r* = 0.136, calcium; mean *r* = 0.109, blood urine nitrogen; mean *r* = 0.097, body weight), and in each case the average correlation differed significantly relative to all those represented on the array platform (P<2.07×10^−56^). These analyses indicate that hepatic expression of Mφ-associated transcripts is correlated with multiple phenotypic measures that are linked to pathological aspects of the HF diet.

### Greater than 50% of high fat-increased transcripts can be explained by hepatic infiltration of monocytes, macrophages or dendritic cells in some mouse strains

**Figure 10:**
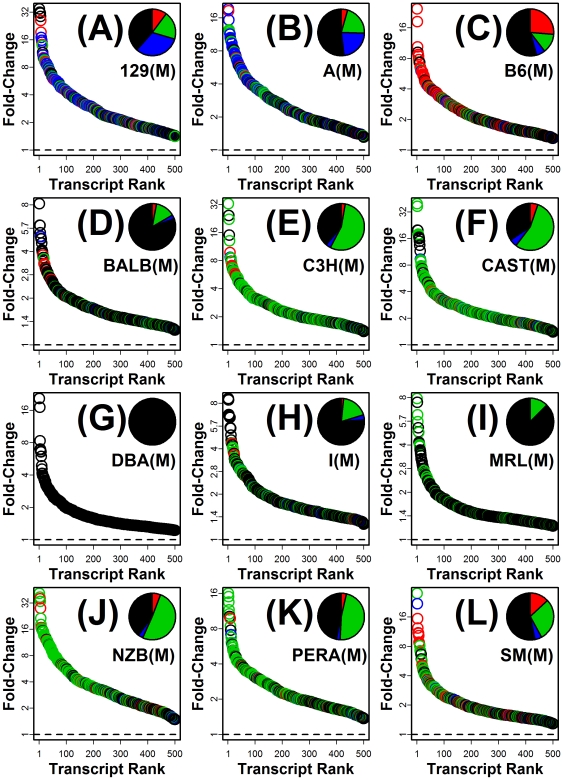
Monocyte, macrophage and dendritic cell infiltration explains gene expression patterns associated with high fat (HF) diet in multiple mouse strains (males). The 500 transcripts for which expression was most strongly increased by HF diet were identified for each strain. The 500 transcripts were first identified on the basis of FDR-adjusted p-values and then ranked according to the fold-change estimate associated with HF diet (i.e., HF diet/control). Panels correspond to male mice of the following strains: (A) 129S1/SvImJ, (B) A/J, (C) C57BL/6J, (D) BALB/cJ, (E) C3H/HeJ, (F) CAST/EiJ, (G) DBA/2J, (H) I/LnJ, (I) MRL/MpJ-Tnfrs6lpr/J, (J) NZB/BINJ, (K) PERA/Ei and (L) SM/J. Each panel plots ranked fold-change estimates associated with the top 500 HF-increased transcripts. Of the top 500 transcripts, some were signature transcripts of dendritic cells, macrophages or monocytes, and in such cases, increased expression with HF diet was explainable on the basis of leukocyte infiltration. In each panel, those HF-increased transcripts that were explained by dendritic cell, macrophage or monocyte infiltration are represented by red, green and blue symbols, respectively. HF-increased transcripts not associated with these cell types are represented by black symbols. Pie charts indicate the proportion of HF-increased transcripts for which increased expression by high fat diet appeared to be explained by dendritic cell infiltration (red), macrophage infiltration (green), or monocyte infiltration (blue), with the black region corresponding to the fraction of transcripts explained by some other cell type or for which no explanation appeared likely.

What proportion of transcripts elevated by HF diet in liver might be attributable to infiltration by monocytes, Mφ, or DCs as part of an inflammatory response? To address this question, we evaluated the 500 transcripts most strongly increased by HF diet for each of the 24 strain-gender combinations (Figures 10 and S6). For each strain-gender combination, we evaluated the top 500 transcripts individually, and for a given transcript, we determined whether that transcript had been designated as a signature transcript of a high-scoring (statistically significant) cell population in the inflammation profile calculated for the corresponding strain-gender combination (Figure S3). If the transcript was indeed a signature transcript of a significant population, that transcript was “assigned” to that cell population, and this served as an explanation for why that transcript was elevated by HF diet in liver. In males, the proportion of HF-increased transcripts explained by monocytes, Mφ, or DCs varied between 12.4% (MRL males) and 65.6% (CAST males) (Figure 10). An exception was DBA males, for which none of the top 500 transcripts were explained on this basis (because no significant cell populations were identified in the inflammation profile for DBA male mice; see Figure S3). In females, we estimated that between 7.2% (DBA females) and 64.8% (CAST females) of the top 500 transcripts were, potentially, explainable on the basis of monocytes, Mφ, or DC infiltration (Figure S6).

## Discussion

High fat diets can promote systemic inflammation and may lead to a metabolic state associated with negative long-term health outcomes [3]–[7]. Microarray experiments have provided a systems-level view of the major effects of high fat (HF) diet within individual tissues, and have shown that HF diet often increases the expression of genes disproportionately associated with immune system processes, such as the processing and presentation of antigens [15], [21]–[24]. We hypothesized that, in liver, this effect of HF diet is due to infiltration of hepatic tissue by white blood cells, and we have applied an algorithm to test whether this hypothesis is consistent with data from the Novartis strain-diet-sex survey (GSE10493). This hypothesis was supported by our analyses and we show that “signature transcripts” highly expressed in certain leukocyte populations (e.g., thioglycollate-elicited peritoneal Mφ) are often overwhelmingly elevated in hepatic tissue of mice provided the high fat diet. This effect, which appears to reflect the degree of localized hepatic inflammation, has a genetic component and differs in intensity among males and females of 12 mouse strains. In some strains (NZB male and female mice, B6 males and A/J females), we estimate that 50–60% of genes elevated by HF diet can be explained on the basis of leukocyte infiltration. On the other hand, in DBA males and females, there was little or no indication that hepatic gene expression patterns associated with HF diet were shaped by leukocyte infiltration. These results, taken together, suggest that inflammatory processes and resultant tissue remodeling partly explain genome-wide expression patterns associated with HF diet, that microarray data can be exploited to gain biological insights into these patterns, and that the intensity of hepatic inflammation in response to HF diet is genetically regulated and heterogeneous among inbred mouse strains.

The HF diet perturbs the hepatocyte-leukocyte balance that normally exists within the liver microenvironment [17], [25], [26], and there are multiple (non-mutually exclusive) models that may explain how the inflammatory cascade is initiated by HF diet to ultimately promote an influx of leukocytes into the liver [19]. Healthy liver contains a balance of hepatocytes and other intrahepatic cell populations, including resident macrophages (Kupffer cells), in addition to immune cells entering from circulation. This balance can be altered by triggers that increase generation of chemoattractant compounds by hepatocytes to enhance the recruitment of immune cells, or by cellular modifications intrinsic to circulating leukocyte populations that increase their capacity for adhesion or migration into endothelial tissues [19]. In the present context, *in silico* inflammation profiles have provided a tool for gauging the relative intensity of inflammatory gene expression in different mouse strains, and also provide an unbiased method for identifying populations that appear most likely to explain observed effects of HF diet on hepatic gene expression. For most mouse strains, there was strong evidence that HF diet increased the hepatic expression of transcripts highly expressed in bone marrow-derived cells from the monocyte- Mφ and monocyte-DC lineages (Figure 7). This inference is based upon marked and statistically significant patterns in the data (e.g., see Figure 4; FDR-adjusted P-value less than 10^−150^), and is also in agreement with previous immunohistochemical investigations, which have shown that HF diet increases abundance of these cell types in mice of multiple genetic backgrounds [17], [25], [27]. We characterized a gene expression phenotype associated with high-scoring cell populations, with the expectation that the capacity of cells to home to liver tissue and attach to endothelial substrates would depend upon expression of receptors or key cellular components. This analysis revealed that a unifying characteristic of cell populations assigned high scores on inflammation profiles was high expression of genes encoding components of the toll-like receptor (TLR) signaling pathway (e.g., *Irf5* and *Myd88*) (Figure 9). Our findings, therefore, add to the growing recognition of this pathway as a contributor to obesity-associated inflammation [11], [17], [28]–[36], and suggest that heightened expression of TLR genes in several mouse strains may predispose leukocytes to respond to *in vivo* signals that arise due to excessive fat intake (e.g., elevated fatty acids). This possibility is consistent with findings from recent studies, which have shown that deletion of toll-like receptor 4 (*Tlr4*) in hematopoietic cells abrogates HF diet-associated inflammation in liver and adipose tissue [29], and that liver damage due to adoptive transfer of immature myeloid cells (CD11b^+^Ly6C^hi^Ly6G^−^) from HF-fed mice occurs only when donor cells carry the gene encoding the Myd88 intracellular adaptor protein [25].

Development of hepatic inflammation depends upon several parallel mechanisms and the interplay between circulating cytokines and concurrent processes in both hepatocytes and nonparenchymal cells. For instance, HF diet may stimulate hepatocytes to increase local production of monocyte chemoattractant protein-1 (MCP-1/CCL2), which serves as a chemoattractant signal that would draw monocytes from circulation, which could then differentiate locally into Mφ or DC progeny cells [37]. Indeed, inspection of the Novartis strain-diet-sex database reveals that, on average among all 24 strain-gender combinations, expression of *Mcp-1*/*Ccl2* is increased by 58% (range: 4% decrease in SM females; 3-fold increase in NZB males and A females; significant increase in 16 of 24 cases). This strengthening of a chemoattractant gradient could, moreover, be accompanied by increased fractional abundance of the circulating inflammatory Ly-6C^hi^ monocyte subset [38], which express specialized ligands that facilitate tethering of monocytes to endothelial substrates [39]. Hepatic inflammation may also be heighted by damage to liver tissue associated with HF diet [15]. The hepatic blood supply from the portal vein, for example, is highly sensitive to alterations in the absorption capacity of the intestinal mucosa. Along these lines, systemically high cytokine levels associated with HF diet may disrupt tight junction complexes in the intestine to compromise intestinal permeability, ultimately leading to elevated endotoxin levels in the portal blood supply, which may damage liver tissue and promote release of inflammatory compounds by hepatic stellate cells and Kupffer cells [40]. Lastly, given a HF diet, inflammatory signals emitted by interstitial fat deposits in hepatic tissue may be enhanced, which would serve to tighten the association between such fat deposits and white blood cells. Inflammation profiles, for instance, suggested increased presence of T cell-associated transcripts in liver of HF-fed mice of most strains (Figure 6), and this could arise from a closer association with intra-hepatic adipose tissue and T lymphocytes [6], [7], [41]–[43]. At the same time, signature transcripts of white adipose tissue were also disproportionately elevated by HF diet, suggesting that some degree of fat expansion occurred with HF diet, which could augment still further the abundance of adipose-associated T cell subsets and proinflammatory signals derived from adipose, leading to accelerated lymphocyte recruitment and an overall reinforcement of the inflammatory cascade [44].

It is increasingly recognized that genotype is an important factor shaping response to dietary intervention in mice [45]–[51]. To understand such genotype-by-environment interactions, the diversity of existing mouse strains provides a valuable research tool [49]. Following 4 weeks of the HF diet, nearly all strains exhibited increased abundance of leukocyte-associated transcripts in liver, indicative of localized inflammation, but this response was markedly attenuated in DBA mice. In DBA males, transcripts associated with certain leukocyte populations were disproportionately *decreased* by HF diet (e.g., CD8+ T cells, B cells, DCs and Mφ), providing evidence that the diet had possible anti-inflammatory effects in mice of this strain and gender. This observation is supported by an independent analysis recently reported by Zhu et al. [50], which showed that after 1–21 weeks of HF diet (0.5% cholic acid, 1.25% cholesterol, 15% fat), hepatic tissue of female DBA mice exhibited decreased expression of genes associated with “immune response and inflammation”, while the opposite pattern was observed in B6 mice. Additionally, Zhu et al. [50] noted that, relative to B6 mice, apoptosis of hepatic cells was reduced in DBA females. The present study, therefore, in combination with the results of Zhu et al. [50], suggests that for HF diets of 1–21 weeks in duration, DBA mice of both genders exhibit some level of resistance to hepatic inflammation. It is uncertain whether this response of DBA mice to HF diet is strictly liver-specific, or whether it is indicative of an aberrant pattern by which systemic inflammation progresses in this mouse strain. Previously, Kirk et al. [52] compared nine inbred mouse strains and showed that, in DBA mice, plasma cholesterol levels were unusually hyporesponsive to multiple HF diets, which may have been due to decreased absorption of dietary cholesterol [48], [52]. In other studies, however, in which slightly different HF diets were evaluated, plasma cholesterol levels have been observed to increase in DBA mice [49]. DBA mice also appear susceptible to increased body weight when provided a HF diet. A recent study, in fact, evaluated 42 inbred mouse strains provided an atherogenic diet for 18 weeks, and showed that DBA mice gained more body weight than any other strain [53], [54]. Lastly, DBA mice may exhibit a differential response to healthy diets, such as caloric restriction, which is a well-studied anti-inflammatory diet known to increase lifespan in most inbred mouse strains [55]. In particular, Forster et al. [46] reported a series of experiments in which long-term caloric restriction increased lifespan in B6 mice and B6D2F1 hybrids, but led to a slight lifespan *decrease* in mice of the DBA strain.

The present analysis indicates that, following 4 weeks of HF diet, inflammatory gene expression patterns in hepatic tissue of different mouse strains can vary. It is important to note, however, that these observations correspond to a fixed time point following the start of HF diet (i.e., 4 weeks), and that development of hepatic inflammation may proceed along a time course, with a more acute short-term phase followed by a longer-term chronic phase in which inflammation is partly attenuated [21], [22]. A recent study of hepatic gene expression data in Apo3Leiden mice (B6 background), for example, has indicated that an acute inflammatory response to HF diet develops in the short-term (1 day–1 week), but that this response partly attenuates between 8 to 16 weeks with the progression of steatosis [21]. In our analysis, the 4 weeks of HF diet is neither a short-term or long-term response, but can be viewed as an intermediate time point, which may lie at the transition between distinct phases of the hepatic or systemic responses to HF diet. Potentially, differences between mouse strains with respect to inflammation intensity that we observed may not represent variations in the intensity of hepatic inflammation developing in each strain, but rather HF-response patterns that are temporally out-of-step between strains. In the case of DBA mice, one possibility is that hepatic inflammation occurs more rapidly relative to other strains, such that after 4 weeks of HF diet, DBA mice are at an advanced and less acute inflammatory stage and thus falsely appear to exhibit resistance to hepatic inflammation. However, the study of Zhu et al. [50] (see above) argues against this possibility, since Zhu et al. [50] showed that inflammatory gene expression in DBA females was hypo-responsive to HF diet across a range of time points between 1 and 21 weeks. Nevertheless, we note that data analyzed in this analysis are specific to the 4-week time point, and thus do not permit evaluation of the temporal progression of HF-associated inflammatory gene expression patterns among the 12 inbred strains evaluated. The generation of a more comprehensive dataset, involving a diversity of strains and a time series of gene expression profiles, would be a challenging task, but would provide a basis for understanding how progression of hepatic inflammation in response to HF diet may differ among inbred mouse strains.

This study has provided a targeted analysis of hepatic gene expression patterns in HF-fed and control mice, and results are consistent with the hypothesis that hepatic infiltration of white blood cells is a robust response to HF diet in mice that occurs in multiple strains and both genders. These findings provide a reference point for future studies investigating effects of HF diet in various mouse strains and both genders. Further work should be directed at evaluating whether HF diet promotes a similar microarray-based inflammation signature in non-hepatic tissues, evaluating how strongly inflammation signatures correlate with results generated from immunohistochemical analyses, and determining whether supplementation of HF diets with cholic acid augments inflammatory processes [16]. While our results indicate that development of hepatic inflammation is a robust response to 4 weeks of HF diet, we have also shown that this response is not universal and appears attenuated in DBA mice. This result highlights the strain-specificity of dietary responses, which challenges efforts to construct general models of dietary response that will have applicability to multiple mouse strains, to other species, and potentially, to humans. For this reason, further generation of comprehensive multi-strain datasets is needed to ensure that conceptual models of dietary response are not genotype-specific or dependent upon the properties of a single mouse genotype. Finally, findings from this study demonstrate that a complex inflammatory process, involving shifts in cellular composition combined with altered transcription within cells, is indeed amenable to computational analysis guided by a biological rationale. This *in silico* strategy is likely to be equally useful in other contexts in which gene expression patterns are the cumulative product of intracellular processes and a broader inflammatory response (e.g., cancer, neurodegeneration, psoriasis, atherosclerosis, aging).

## Methods

### Novartis strain-diet-sex microarray database

The effects of high fat (HF) diet were analyzed using the Novartis strain-diet-sex survey database, which can be obtained from Gene Expression Omnibus under accession numberGSE10493. The complete dataset, consisting of 144 CEL files, was downloaded and expression scores were subsequently calculated using the robust multichip average (RMA) method. We note that a web-based query tool for exploring these data has been made available online at http://cgd-array.jax/org/DietStrainSurvey [15]. The experimental procedures associated with animal care and tissue processing have been described by Shockley et al. [15]. In brief, for HF treatments, mice were provided a diet with 30% Kcal from dairy fat, which contained 1% cholesterol by weight and 0.5% cholic acid. For control treatments, mice were provided with a standard diet containing only 6% fat (Cat. No. 5K52, Lab Diets, St. Louis, MO). Animals were housed in specific pathogen free facilities prior to sacrifice at 10–13 weeks of age (20–55 weeks of age in the case of CAST/EiJ and PERA/Ei mice). Mice were fasted approximately 5 hours before sacrifice and perfused with saline before dissection. Phenotypic data collected from mice utilized in these experiments can be accessed and downloaded from http://cgd.jax.org/datasets/expression/10strain.shtml. Further details regarding general phenotypic characteristics of each mouse strain can be obtained from the Mouse Phenome Database (MPD) [56] and a convenient webpage with MPD links for each of the 12 mouse strains considered in our analysis is available (see: http://phenome.jax.org/db/q?rtn=projects/strainlist&projsym=GX-Shockley1).

### Algorithm for calculation of inflammation profiles

Inflammation profiles were calculated for each of 12 mouse strains and both genders (i.e., 24 strain-gender combinations). Inflammation profiles are an *in silico* device that can be used to interpret gene expression patterns, and in the present context, are used to gauge intensity of inflammation in response to HF diet, and to infer which types of leukocytes best explain hepatic gene expression differences between HF-fed and control mice. The overall process is summarized in Figure 2. In step 1, a set of *n* signature transcripts is identified for each of *j* = 1,…, *N* leukocyte populations. This step required two sets of reference microarray data (i.e., treatments *A* and *B* in Figure 2), both of which correspond to data obtained from Gene Expression Omnibus, which are further described in Tables S1 and S2. All data samples listed in Tables S1 and S2 were generated using the Affymetrix 430 2.0 oligonucleotide microarray, which is the same platform utilized in the Novartis strain-diet-sex survey. The first set of reference data (i.e., treatment *A*) corresponds to a batch of *n*_A_ = 65 CEL files obtained from GEO, where each CEL file was generated from an array hybridization with source material isolated from mouse liver (Table S1).The second set of reference data (i.e., treatment *B*) corresponds to a batch of *n_B_* CEL files from the *j*th leukocyte population (Table S2). For each leukocyte population evaluated, there were at least two replicate samples available (i.e., *n_B_*≥2), and on average, there were 2.80 replicates available per population. To identify *n* signature transcripts for any one leukocyte population, *n*_B_ CEL files associated with that population were jointly normalized with the liver reference dataset (i.e., the *n*_A_ = 65 CEL files). The LIMMA algorithm (linear models for microarray data) was then used to identify transcripts differentially expressed between the leukocyte population and the liver reference data. We utilized a high threshold for the identification of transcripts with significantly higher expression in the leukocyte population (i.e., FDR-adjusted P-value<10^−4^ and fold-change≥16), with FDR-adjustment of p-values carried out using the Benjamini-Hochberg method [57]. Given this threshold, there was, on average, *n* = 594 signature transcripts identified for each leukocyte population evaluated in our analysis (range: 152≤*n*≤1311). The set of *n* signature transcripts is then analyzed, in step 2, to determine if the set is disproportionately elevated by HF diet in liver tissue. If a leukocyte population invades hepatic tissue with HF diet, the *n*_1_ signature transcripts increased by HF diet should greatly exceed the *n*_2_ signature transcripts decreased by HF diet, and the ratio *n*_1_/*n*_2_ should be large (where *n* = *n*_1_+*n*_2_).

Inflammation profiles show, for each strain-gender combination, the ratio *n*_1_/*n*_2_ generated from steps 1 and 2 (as shown in Figure 2). However, it is expected that, for any given population, some of the *n* signature transcripts will also be signature transcripts of other populations. For instance, there were 19,900 pairwise combinations among the *N* = 200 cell populations evaluated in our analysis. For each pairwise combination, we evaluated the overlap of signature transcripts and found that the median level of overlap, among all pairwise combinations, was 25.39% (1st quartile: 19.54%; 3rd quartile: 32.41%). Such overlap of signature transcripts can be problematic, since it may be that a given cell population does not invade hepatic tissue of HF-fed mice, but nevertheless, the cell type shares many signature transcripts with another population that actually does invade hepatic tissue of HF-fed mice. In this case, the non-invading population may have a large *n*_1_/*n*_2_ ratio, and thus appear to invade hepatic tissue, when in fact it does not. To address this potential problem, we also evaluate a second ratio for each population, *n*_1*_/*n*_2*_, which is generated from steps 3 and 4 (see Figure 2). The ratio *n*_1*_/*n*_2*_ is calculated based upon a reduced set of n* signature transcripts, which for each population, is obtained by filtering the *n* signature transcripts, in order to exclude any transcripts that are also signature transcripts for another population with a larger *n*_1_/*n*_2_ ratio (i.e., step 3 in Figure 2). The n* signature transcripts therefore represent transcripts highly expressed in a particular leukocyte population (relative to liver), which are uniquely expressed in that population relative to any other populations for which the ratio *n*_1_/*n*_2_ is larger. For nearly all populations, therefore, the value of n* is less than *n*, with the exception of the highest-ranked population (with the largest *n*_1_/*n*_2_ ratio), for which no sub-setting can be performed and *n* = n* (see Figure 2). The advantage of considering both *n*_1_/*n*_2_ and *n*_1*_/*n*_2*_ ratios is that, for populations not invading hepatic tissue but sharing many signature transcripts with other populations that do invade hepatic tissue, the ratio *n*_1_/*n*_2_ would be large but the ratio *n*_1*_/*n*_2*_ would not be. The consideration of both *n*_1_/*n*_2_ and *n*_1*_/*n*_2*_ ratios to evaluate statistical significance (see below) thus serves to de-correlate patterns of statistical significance and to reduce the chance that some populations will spuriously emerge as significant because their signature transcripts overlap with those of another high-scoring population.

### Leukocyte populations evaluated in inflammation profiles

Most of the *N* = 200 populations evaluated in our procedure were white blood cell populations. For convenience, we have collectively referred to these as “leukocyte populations” throughout this paper. We note, however, that not all populations represented among the 200 we evaluate are in fact leukocytes (Table S2). Some populations correspond to RNA extracted from certain types of progenitor cells isolated from bone marrow, which are precursors to circulating blood cells (e.g., hematopoietic stem cells, common lymphoid progenitors, erythroblasts; see Table S2). In other cases, populations correspond to RNA extracted from whole organs (e.g., thymus, spleen, white adipose tissue; see Table S2). These populations were evaluated in parallel with true white blood cells subsets because of their role in leukocyte development (e.g., thymus, spleen, progenitors). In the case of adipose tissue, it was suspected that HF diet could augment adipose tissue deposits in liver, and thus promote elevated expression of adipose-associated transcripts by a mechanism comparable to the elevated expression of immune-associated transcripts due to white blood cell infiltration.

### Statistical significance criteria for inflammation profiles

The inflammation profiles presented in our analysis (e.g., Figure 3 and Figure S3) include red symbols that correspond to populations for which statistical significance criteria were satisfied, along with black symbols, which represent populations for which significance criteria were not satisfied. There were two criteria for statistical significance. First, the ratio *n*_1_/*n*_2_ needed to be significantly large, as compared to the ratio of HF-increased to HF-decreased transcripts observed among all 45,101 transcripts represented on the Affymetrix 430 Mouse Genome 2.0 array. The significance of this ratio was evaluated using a hypergeometric test to determine the likelihood of observing *n*_1_ HF-increased transcripts within a sample of *n*_1_+*n*_2_ transcripts sampled from all those evaluated on the Affymetrix 430 Mouse Genome 2.0 array. P-values generated from this test were then adjusted for multiple testing among all 200 populations using the Hochberg method [58], which is a conservative p-value adjustment method that is valid for the case in which p-values are non-negatively correlated. We note that, while the value *n*_1_ is the test statistic considered by the hypergeometric test, the value of *n*_1_ is directly proportional to *n*_1_/*n*_2_, and thus we view this approach as a test of the *n*_1_/*n*_2_ ratio.

The second criteria for statistical significance was the same test, except applied to the ratio *n*_1*_/*n*_2*_ rather than *n*_1_/*n*_2_. For this test, the hypergeometric distribution was used to determine, for each population, whether the observed value of *n*_1*_ was significantly large, given the null scenario in which a random sample of *n*_1*_+*n*_2*_ transcripts are chosen from all those evaluated on the array platform. As above, p-values generated from this test were adjusted using the conservative Hochberg correction to account for multiple testing among the 200 leukocyte populations. For inflammation profiles presented in this study (Figure 3 and Figure S3), red symbols correspond to populations for which both hypergeometric tests (as applied to *n*_1_/*n*_2_ and *n*_1*_/*n*_2*_) are significant (FDR-adjusted P<0.05). The main advantage of using the hypergeometric distribution to evaluate significance of *n*_1_/*n*_2_ and *n*_1*_/*n*_2*_ ratios is that the approach is straightforward and does not require the repeated simulation of a null distribution for every leukocyte population considered, which would have been computationally expensive in the present context. We note, however, that our algorithm could be adapted to utilize alternative statistical measures of correspondence between leukocyte-associated and HF-associated gene expression patterns, such as the test statistic used in the gene set enrichment analysis (GSEA) algorithm proposed by Subramanian et al. [59].

## Supporting Information

Figure S1 Gene ontology biological processes overrepresented among transcripts increased by high fat diet in males of 12 mouse strains. Transcripts significantly increased by high fat (HF) in males were identified with respect to 12 mouse strains (see Table 1; FDR-adjusted P<0.05). For each set of genes exhibiting significantly increased expression with HF diet, significantly overrepresented gene ontology (GO) terms were identified (see Falcon and Gentleman 2007, Bioinformatics 23:257–58). GO terms have been clustered according to a similarity measure based upon the number of GO ancestor terms shared between any two GO terms. Terms overrepresented with respect to 9+, 8 or 7 mouse strains are shown as red, blue and green font, respectively. All other terms, overrepresented with respect to fewer than 7 mouse strains, are shown as standard black font.
(0.03 MB PDF)

Figure S2 Gene ontology biological processes overrepresented among transcripts increased by high fat diet in females of 12 mouse strains. Transcripts significantly increased by high fat (HF) in females were identified with respect to 12 mouse strains (see Table 1; FDR-adjusted P<0.05). For each set of genes exhibiting significantly increased expression with HF diet, significantly overrepresented gene ontology (GO) terms were identified (see Falcon and Gentleman 2007, Bioinformatics 23:257–58). GO terms have been clustered according to a similarity measure based upon the number of GO ancestor terms shared between any two GO terms. Terms overrepresented with respect to 9+, 8 or 7 mouse strains are shown as red, blue and green font, respectively. All other terms, overrepresented with respect to fewer than 7 mouse strains, are shown as standard black font.
(0.02 MB PDF)

Figure S3 Inflammation profile associated with high fat diet in 24 strain-gender combinations. The procedure illustrated in Figure 2 was used to calculate inflammation profiles associated with high fat diet for each of 24 strain-gender combinations. In each profile, symbols correspond to individual cell populations, where large ratios (HF-increased/HF-decreased) indicate that signature transcripts of a given population are disproportionately elevated in hepatic tissue of mice provided a high fat diet. The dotted vertical line corresponds to the ratio of HF-increased to HF-decreased transcripts observed among all 45,101 transcripts on the Affymetrix 430 2.0 array platform. Black symbols represent cell populations that did not meet criteria for statistical significance (i.e., the HF-increased/HF-decreased ratio was not larger than expected by chance alone). Red symbols represent cell populations for which statistical significance criteria were satisfied (i.e., the HF-increased/HF-decreased ratio was significantly large; see Methods for description of statistical criteria). The highest-scoring population is represented by a red asterisk symbol rather than an open circle.
(0.11 MB PDF)

Figure S4 Association between male and female inflammation profile results for 12 mouse strains. The procedure illustrated in Figure 2 was used to calculate inflammation profiles for both genders of 12 mouse strains (see Figure S3). For each strain, it was expected that inflammation profile results would be similar between genders; such that, for a given leukocyte population, the ratio of HF-increased to HF-decreased signature transcripts would be similar between males and females. To evaluate this expectation, we compared inflammation profile ratios between male and female mice of a given strain with respect to the 200 leukocyte populations considered in this study. In figures (A)–(L), each point represents an individual leukocyte population. The horizontal axis corresponds to inflammation profile ratios (HF-increased/HF-decreased signature transcripts) calculated with respect to males of the indicated strain, while the vertical axis corresponds to ratios calculated with respect to females of the indicated strain. Both horizontal and vertical axes are log2 scales. The red dashed line is a least-squares regression fit and the Pearson correlation coefficient is listed in the lower right of each figure (A)–(L). Blue symbols are used to indicate leukocyte populations for which statistical significance criteria were satisfied with respect to both males and females (all other leukocyte populations are represented by black symbols; see Methods for description of statistical significance criteria).
(0.18 MB PDF)

Figure S5 Transcripts associated with thioglycollate-elicited peritoneal macrophages exhibit hepatic gene expression patterns that are correlated with total cholesterol. A population of thioglycollate-elicited peritoneal macrophages was identified, on the basis of inflammation profiles (Figure 3 and Figure S3), as the most consistently high-scoring population among the 24 strain-gender combinations. For each of 570 signature transcripts associated with this population, the vertical axis corresponds to the correlation between hepatic gene expression and total cholesterol among 120 mice evaluated in the Novartis strain-gender-diet survey. The horizontal axis corresponds to the fold-change associated with hepatic gene expression levels in liver of B6 males provided a high fat diet (n = 3) relative to controls (n = 3). The dotted horizontal line represents the average correlation value calculated among all 45,101 probe sets included on the Affymetrix 430 2.0 Mouse Genome array (r = −0.079), and the gray region corresponds to this value plus/minus one standard deviation (i.e., −0.079±0.001). The solid red line indicates the average correlation value among the 570 macrophage-associated transcripts (r = 0.556±0.009). The dotted vertical line represents the average fold-change value (HF-diet/Control) among all Affymetrix 430 2.0 transcripts (i.e., 1.00±0.001).
(0.12 MB TIF)

Figure S6 Monocyte, macrophage and dendritic cell infiltration explains gene expression patterns associated with high fat diet in most mouse strains (females). The analysis described in Figure 10 was applied to each of the 12 mouse strains, except the top 500 transcripts increased by high fat diet in females were evaluated. Panels correspond to male mice of the following strains: (A) 129S1/SvImJ, (B) A/J, (C) C57BL/6J, (D) BALB/cJ, (E) C3H/HeJ, (F) CAST/EiJ, (G) DBA/2J, (H) I/LnJ, (I) MRL/MpJ-Tnfrs6lpr/J, (J) NZB/BINJ, (K) PERA/Ei and (L) SM/J.
(0.38 MB TIF)

Table S1 Data samples used to construct reference database for liver tissue (i.e., Treatment A from Figure 2). The procedure described in Figure 2 indicates that n_A_ arrays were used as a reference set to identify signature transcripts associated with a given cell population. A total of n_A_ = 56 arrays were obtained from Gene Expression Omnibus and assigned to this reference set treatment. These 56 hybridizations are listed below. As indicated in the table, each sample corresponds to a hybridization that involves RNA extracted from liver of young male or female mice (≤16 weeks of age).
(0.04 MB PDF)

Table S2 Data samples used to identify signature transcripts for leukocyte populations (i.e., treatment B in Figure 2). In total, N = 200 cell populations were evaluated, and the procedure carried out for the jth leukocyte population is described in Figure 2, where j = 1, …, 200. This table lists all 200 cell populations for which the procedure was applied. Most cell populations represent leukocytes, although some correspond to progenitor cells, thymus, spleen, or white adipose tissue. For each population, the samples listed correspond to replicate CEL files obtained from Gene Expression Omnibus. These samples were used as the n_B_ biological replicates in Treatment B (see Figure 2) in order to identify signature transcripts for each population.
(0.10 MB PDF)
