# Supplementary figures and images for: Transcriptional Profiles of Leukocyte Populations Provide a Tool for Interpreting Gene Expression Patterns Associated with High Fat Diet in Mice
Source: PLoS One. 2010 Jul 29;5(7):e11861. doi: 10.1371/journal.pone.0011861 (PMC2912331; doi:10.1371/journal.pone.0011861)

# HF-Increased Genes: Significant GO Biological Process Terms (Males)

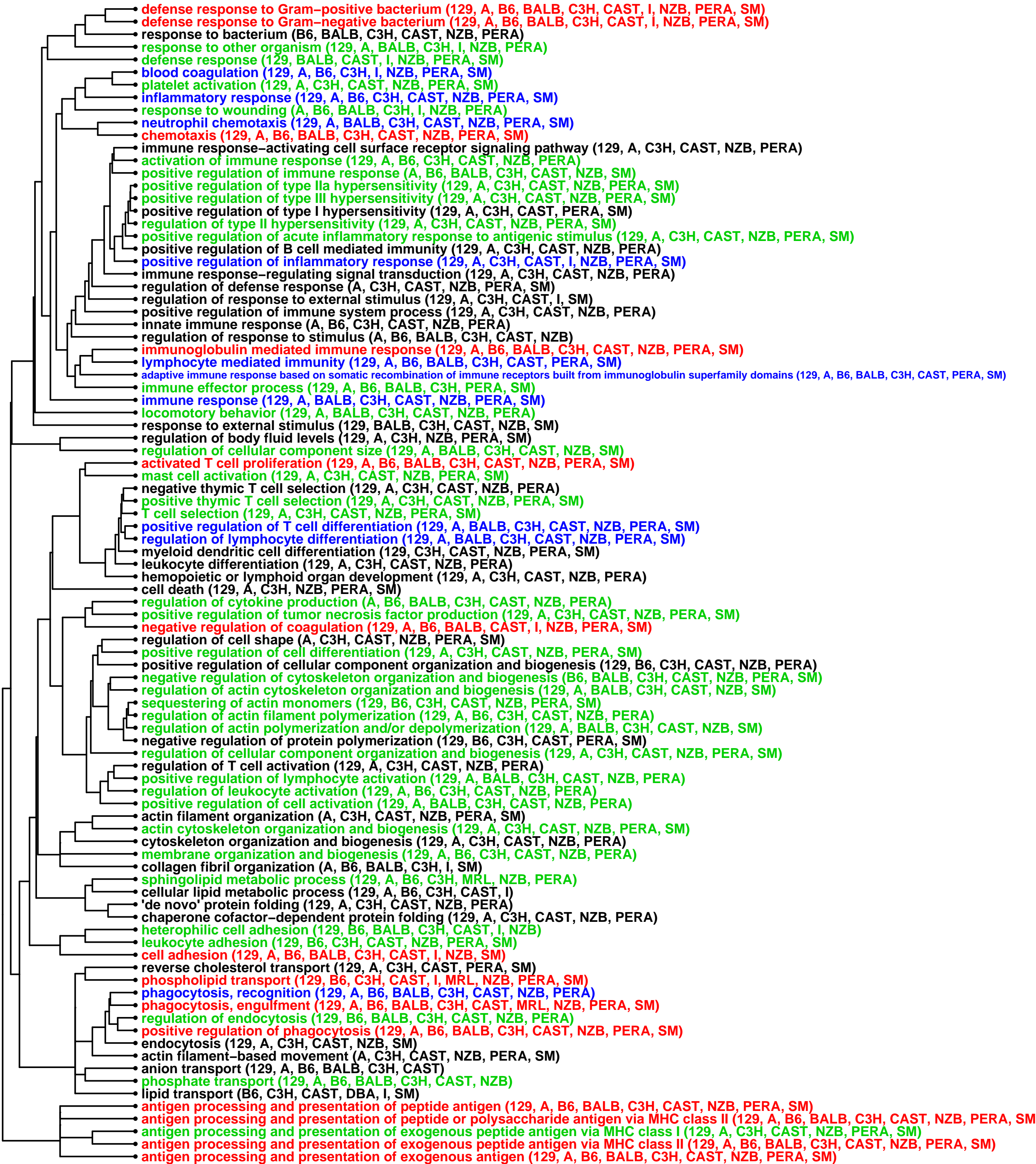

Supplement: Figure S1 — Gene ontology biological processes overrepresented among transcripts increased by high fat diet in males of 12 mouse strains. Transcripts significantly increased by high fat (HF) in males were identified with respect to 12 mouse strains (see Table 1; FDR-adjusted P<0.05). For each set of genes exhibiting significantly increased expression with HF diet, significantly overrepresented gene ontology (GO) terms were identified (see Falcon and Gentleman 2007, Bioinformatics 23:257–58). GO terms have been clustered according to a similarity measure based upon the number of GO ancestor terms shared between any two GO terms. Terms overrepresented with respect to 9+, 8 or 7 mouse strains are shown as red, blue and green font, respectively. All other terms, overrepresented with respect to fewer than 7 mouse strains, are shown as standard black font. (0.03 MB PDF) [file pone.0011861.s001.pdf]

# HF-Increased Genes: Significant GO Biological Process Terms (Females)

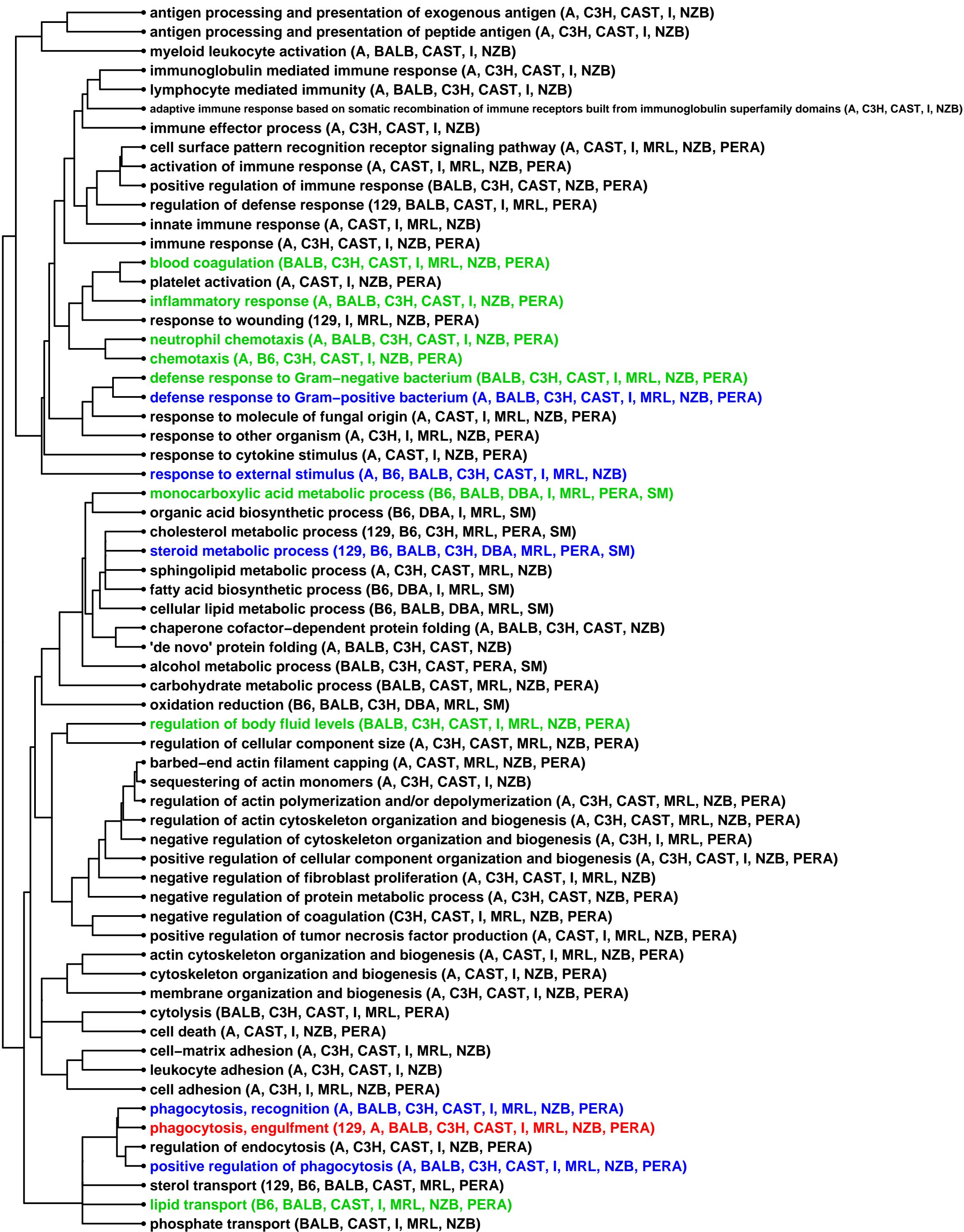

Supplement: Figure S2 — Gene ontology biological processes overrepresented among transcripts increased by high fat diet in females of 12 mouse strains. Transcripts significantly increased by high fat (HF) in females were identified with respect to 12 mouse strains (see Table 1; FDR-adjusted P<0.05). For each set of genes exhibiting significantly increased expression with HF diet, significantly overrepresented gene ontology (GO) terms were identified (see Falcon and Gentleman 2007, Bioinformatics 23:257–58). GO terms have been clustered according to a similarity measure based upon the number of GO ancestor terms shared between any two GO terms. Terms overrepresented with respect to 9+, 8 or 7 mouse strains are shown as red, blue and green font, respectively. All other terms, overrepresented with respect to fewer than 7 mouse strains, are shown as standard black font. (0.02 MB PDF) [file pone.0011861.s002.pdf]

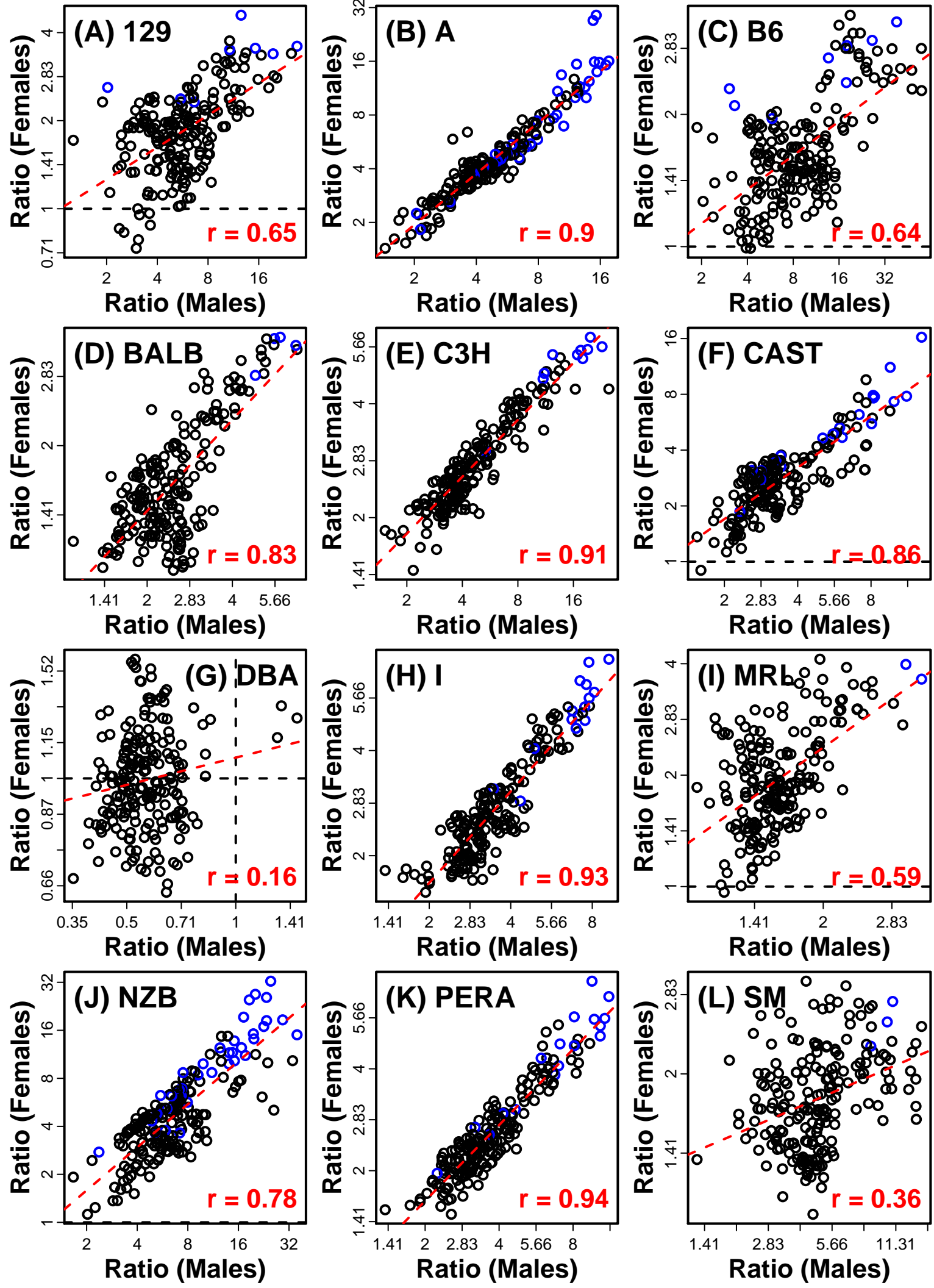

Supplement: Figure S4 — Association between male and female inflammation profile results for 12 mouse strains. The procedure illustrated in Figure 2 was used to calculate inflammation profiles for both genders of 12 mouse strains (see Figure S3). For each strain, it was expected that inflammation profile results would be similar between genders; such that, for a given leukocyte population, the ratio of HF-increased to HF-decreased signature transcripts would be similar between males and females. To evaluate this expectation, we compared inflammation profile ratios between male and female mice of a given strain with respect to the 200 leukocyte populations considered in this study. In figures (A)–(L), each point represents an individual leukocyte population. The horizontal axis corresponds to inflammation profile ratios (HF-increased/HF-decreased signature transcripts) calculated with respect to males of the indicated strain, while the vertical axis corresponds to ratios calculated with respect to females of the indicated strain. Both horizontal and vertical axes are log2 scales. The red dashed line is a least-squares regression fit and the Pearson correlation coefficient is listed in the lower right of each figure (A)–(L). Blue symbols are used to indicate leukocyte populations for which statistical significance criteria were satisfied with respect to both males and females (all other leukocyte populations are represented by black symbols; see Methods for description of statistical significance criteria). (0.18 MB PDF) [file pone.0011861.s004.pdf]

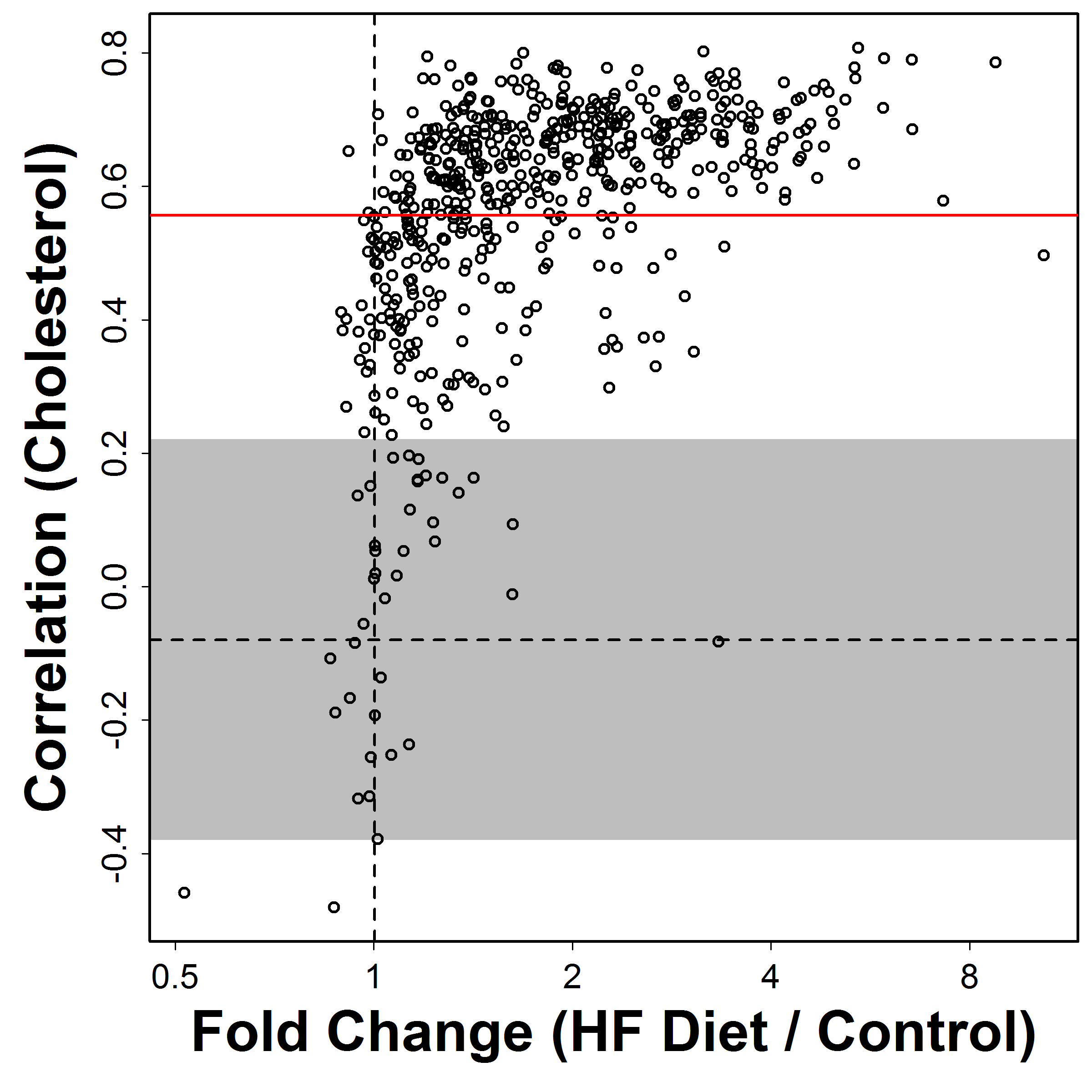

Supplement: Figure S5 — Transcripts associated with thioglycollate-elicited peritoneal macrophages exhibit hepatic gene expression patterns that are correlated with total cholesterol. A population of thioglycollate-elicited peritoneal macrophages was identified, on the basis of inflammation profiles (Figure 3 and Figure S3), as the most consistently high-scoring population among the 24 strain-gender combinations. For each of 570 signature transcripts associated with this population, the vertical axis corresponds to the correlation between hepatic gene expression and total cholesterol among 120 mice evaluated in the Novartis strain-gender-diet survey. The horizontal axis corresponds to the fold-change associated with hepatic gene expression levels in liver of B6 males provided a high fat diet (n = 3) relative to controls (n = 3). The dotted horizontal line represents the average correlation value calculated among all 45,101 probe sets included on the Affymetrix 430 2.0 Mouse Genome array (r = −0.079), and the gray region corresponds to this value plus/minus one standard deviation (i.e., −0.079±0.001). The solid red line indicates the average correlation value among the 570 macrophage-associated transcripts (r = 0.556±0.009). The dotted vertical line represents the average fold-change value (HF-diet/Control) among all Affymetrix 430 2.0 transcripts (i.e., 1.00±0.001). (0.12 MB TIF) [file pone.0011861.s005.tif]

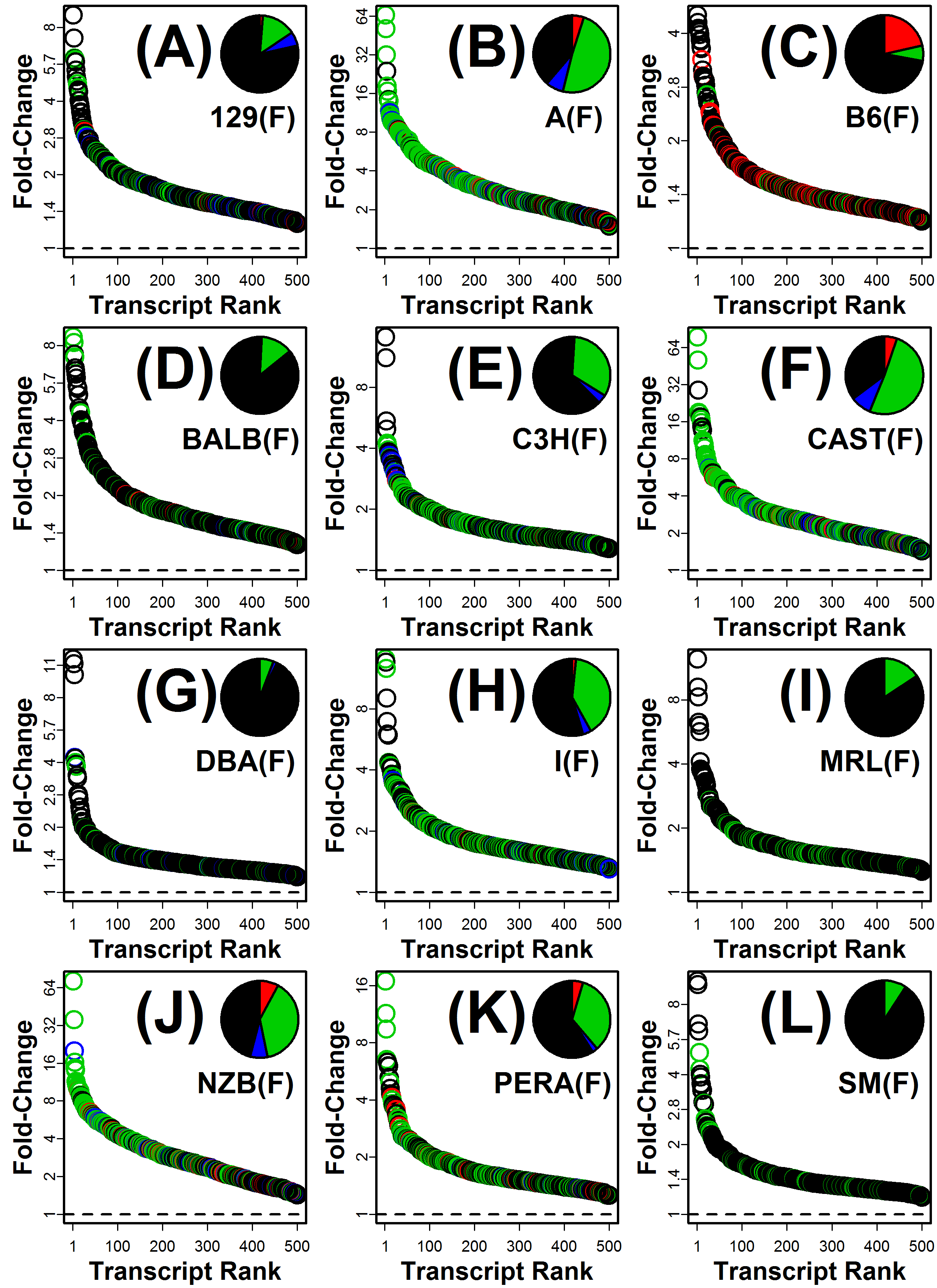

Supplement: Figure S6 — Monocyte, macrophage and dendritic cell infiltration explains gene expression patterns associated with high fat diet in most mouse strains (females). The analysis described in Figure 10 was applied to each of the 12 mouse strains, except the top 500 transcripts increased by high fat diet in females were evaluated. Panels correspond to male mice of the following strains: (A) 129S1/SvImJ, (B) A/J, (C) C57BL/6J, (D) BALB/cJ, (E) C3H/HeJ, (F) CAST/EiJ, (G) DBA/2J, (H) I/LnJ, (I) MRL/MpJ-Tnfrs6lpr/J, (J) NZB/BINJ, (K) PERA/Ei and (L) SM/J. (0.38 MB TIF) [file pone.0011861.s006.tif]
