# Supplementary material for: Transcriptional Profiles of Leukocyte Populations Provide a Tool for Interpreting Gene Expression Patterns Associated with High Fat Diet in Mice
Source: PLoS One. 2010 Jul 29;5(7):e11861. doi: 10.1371/journal.pone.0011861 (PMC2912331; doi:10.1371/journal.pone.0011861)
Supplement: Figure S3 — Inflammation profile associated with high fat diet in 24 strain-gender combinations. The procedure illustrated in Figure 2 was used to calculate inflammation profiles associated with high fat diet for each of 24 strain-gender combinations. In each profile, symbols correspond to individual cell populations, where large ratios (HF-increased/HF-decreased) indicate that signature transcripts of a given population are disproportionately elevated in hepatic tissue of mice provided a high fat diet. The dotted vertical line corresponds to the ratio of HF-increased to HF-decreased transcripts observed among all 45,101 transcripts on the Affymetrix 430 2.0 array platform. Black symbols represent cell populations that did not meet criteria for statistical significance (i.e., the HF-increased/HF-decreased ratio was not larger than expected by chance alone). Red symbols represent cell populations for which statistical significance criteria were satisfied (i.e., the HF-increased/HF-decreased ratio was significantly large; see Methods for description of statistical criteria). The highest-scoring population is represented by a red asterisk symbol rather than an open circle. (0.11 MB PDF) [file pone.0011861.s003.pdf]

Supporting Information File 3

Transcriptional Profiles of Leukocyte Populations Provide a Tool for Interpreting Gene Expression Patterns associated with High Fat Diet in Mice

William R. Swindell, Andrew Johnson, Johann E. Gudjonsson

Inflammation Profiles for 24 Strain-Gender Combinations

The procedure illustrated in Figure 2 was used to calculate inflammation profiles associated with high fat diet for each of the 24 strain-gender combinations evaluated in the Novartis strain-gender-diet dataset (i.e., males and females of 12 mouse strains: 129S1/SvImJ, A/J, C57BL/6J, BALB/cJ, C3H/HeJ, CAST/EiJ, DBA/2J, I/LnJ, MRL/MpJ-Tnfrs6lpr/J, NZB/BINJ, PERA/Ei, SM/J). For each strain-gender combination, this file shows inflammation profiles associated with each strain-gender combination, which can be compared to that associated with B6 male mice (see Figure 3). Each symbol corresponds to an individual cell population evaluated, where large ratios (HF-increased / HF-decreased) indicate that signature transcripts of a given population are disproportionately elevated in hepatic tissue of mice provided a high fat diet. The dotted vertical line shown in each figure corresponds to the ratio of HF-increased to HF-decreased transcripts observed among all 45,101 transcripts on the Affymetrix 430 2.0 array platform. Black symbols represent cell populations that did not meet criteria for statistical significance (i.e., the HF-increased / HF-decreased ratio was not unusual among signature transcripts of such cell populations). Red symbols represent cell populations for which statistical significance criteria were satisfied (i.e., the HF-increased / HF-decreased ratio was unusual among signature transcripts; see Methods for description of statistical criteria). The highest-scoring population is represented by an asterisk symbol (*) rather than an open circle.

Contact: William R. Swindell, wswindell@genetics.med.harvard.edu

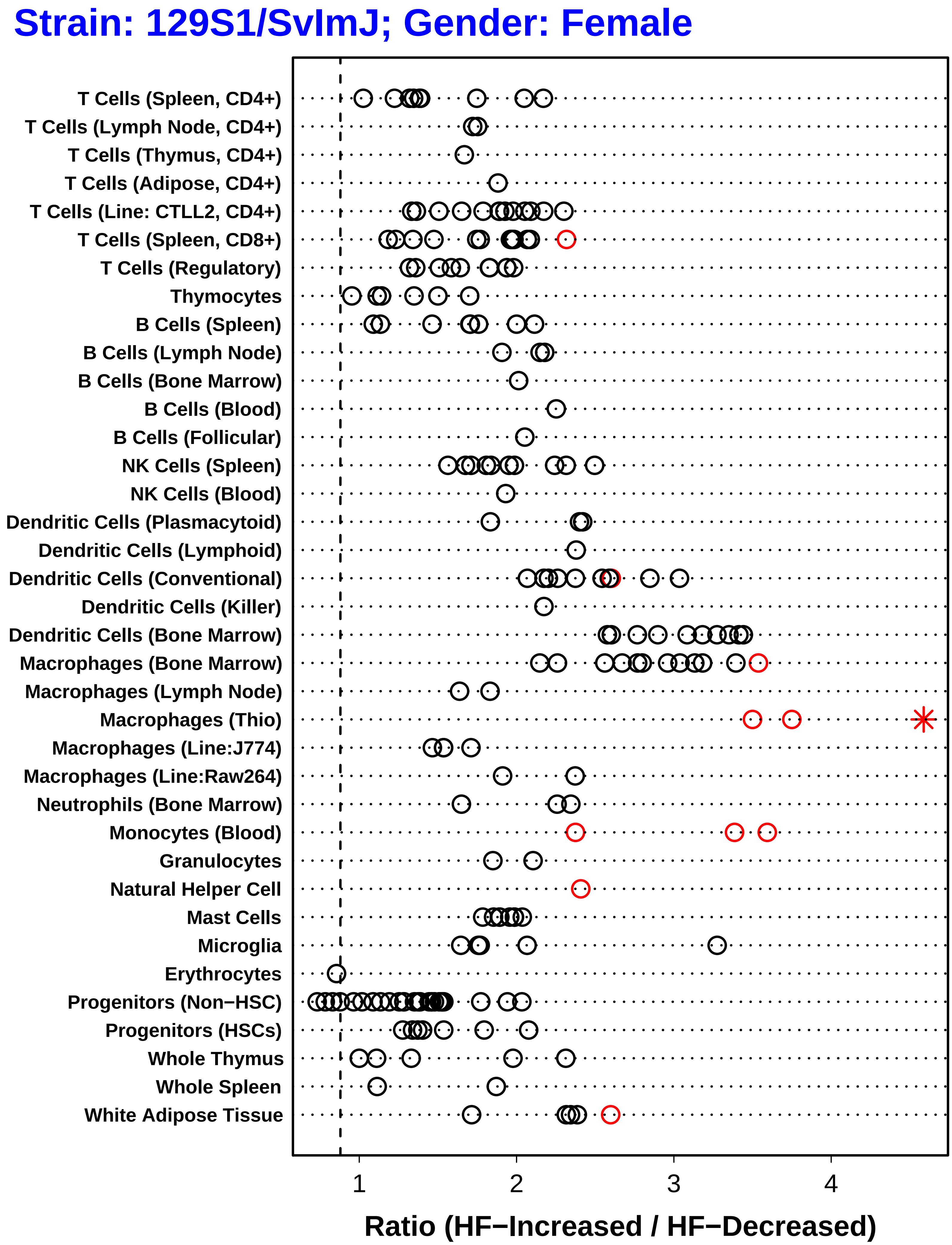

Strain: 129S1/SvImJ; Gender: Female
T Cells (Spleen, CD4+)
T Cells (Lymph Node, CD4+)
T Cells (Thymus, CD4+)
T Cells (Adipose, CD4+)
T Cells (Line: CTLL2, CD4+)
T Cells (Spleen, CD8+)
T Cells (Regulatory)
Thymocytes
B Cells (Spleen)
B Cells (Lymph Node)
B Cells (Bone Marrow)
B Cells (Blood)
B Cells (Follicular)
NK Cells (Spleen)
NK Cells (Blood)
Dendritic Cells (Plasmacytoid)
Dendritic Cells (Lymphoid)
Dendritic Cells (Conventional)
Dendritic Cells (Killer)
Dendritic Cells (Bone Marrow)
Macrophages (Bone Marrow)
Macrophages (Lymph Node)
Macrophages (Thio)
Macrophages (Line:J774)
Macrophages (Line:Raw264)
Neutrophils (Bone Marrow)
Monocytes (Blood)
Granulocytes
Natural Helper Cell
Mast Cells
Microglia
Erythrocytes
Progenitors (Non−HSC)
Progenitors (HSCs)
Whole Thymus
Whole Spleen
White Adipose Tissue
1
2
3
4
Ratio (HF−Increased / HF−Decreased)

Strain: 129S1/SvImJ; Gender: Male

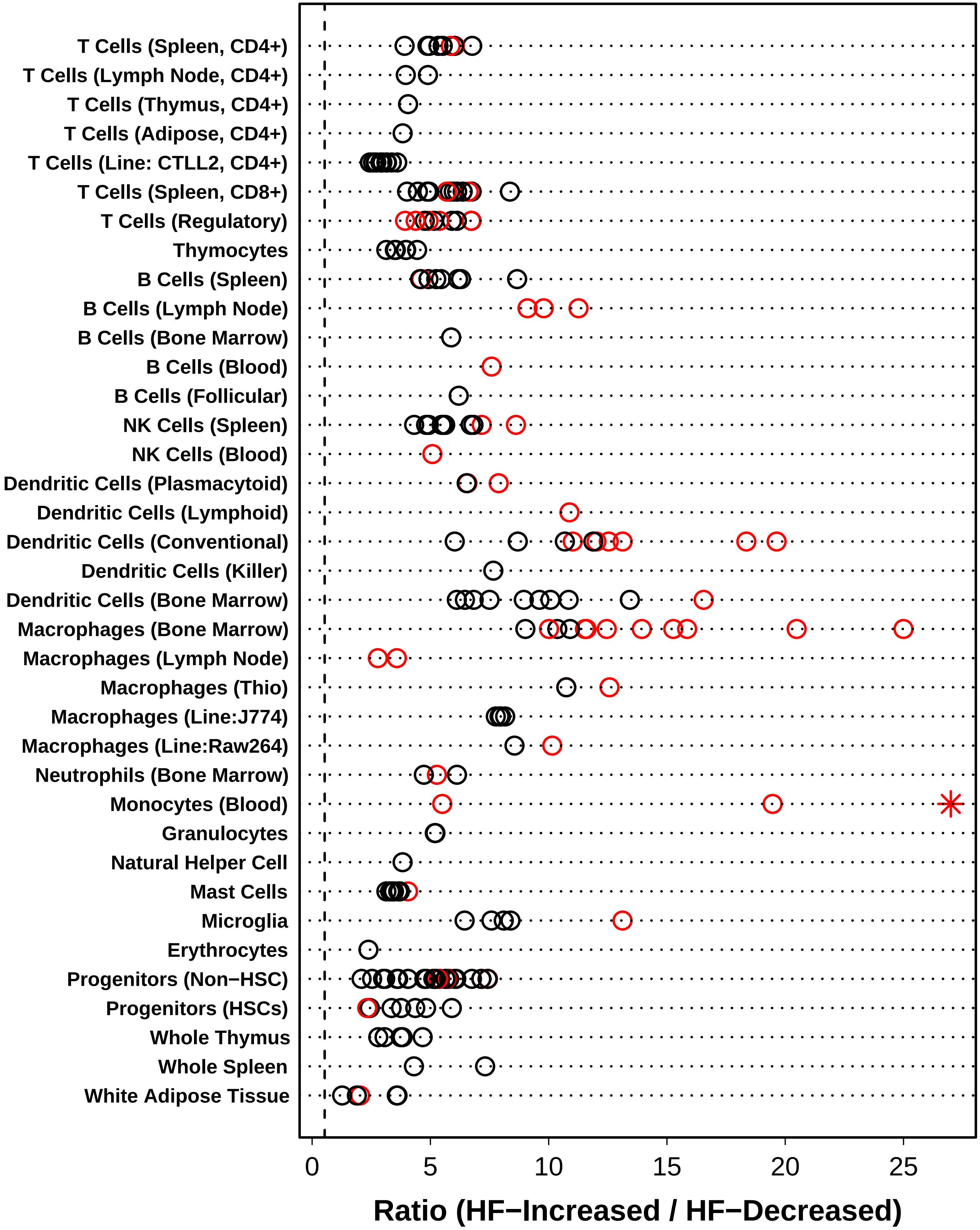

Strain: A/J; Gender: Female

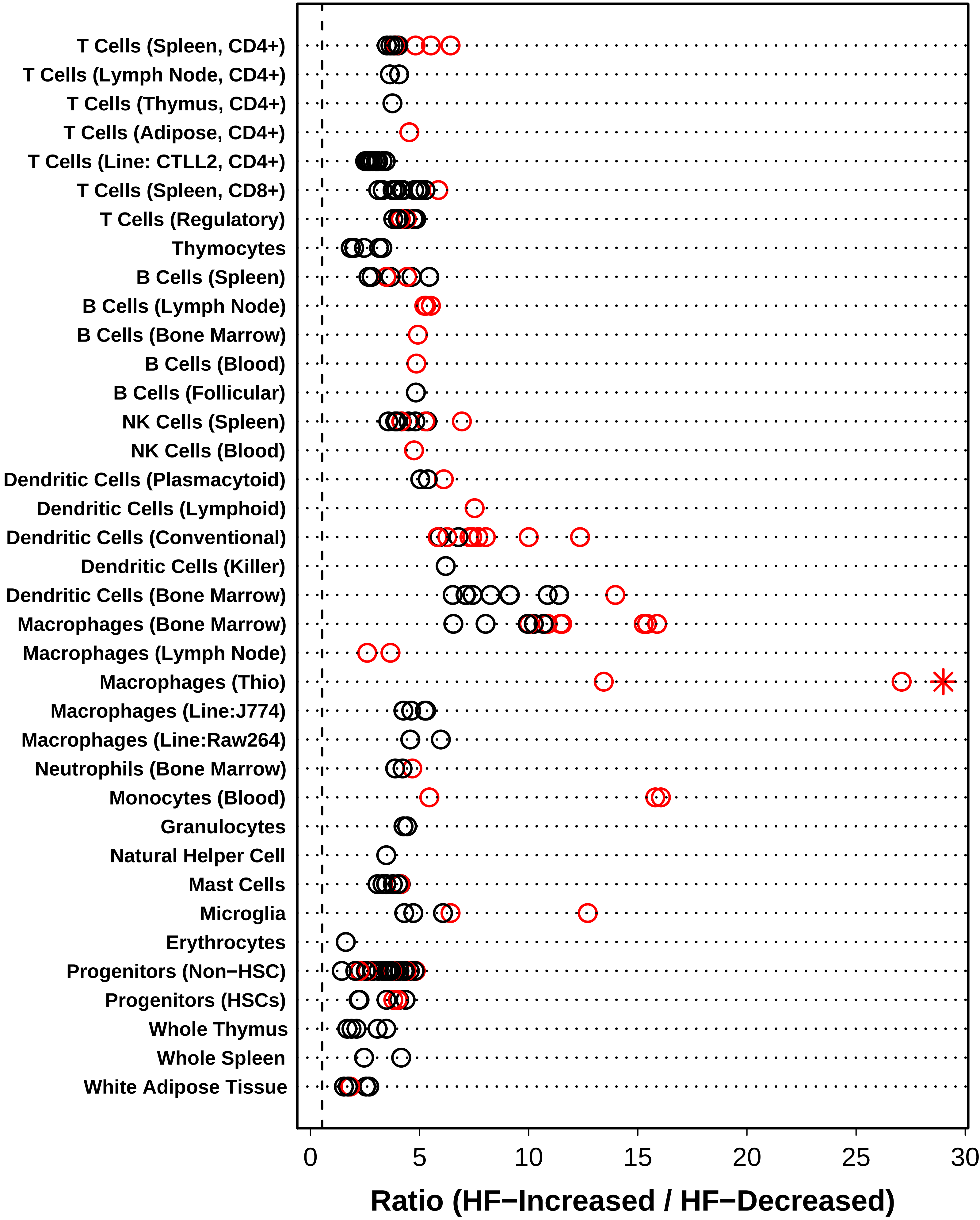

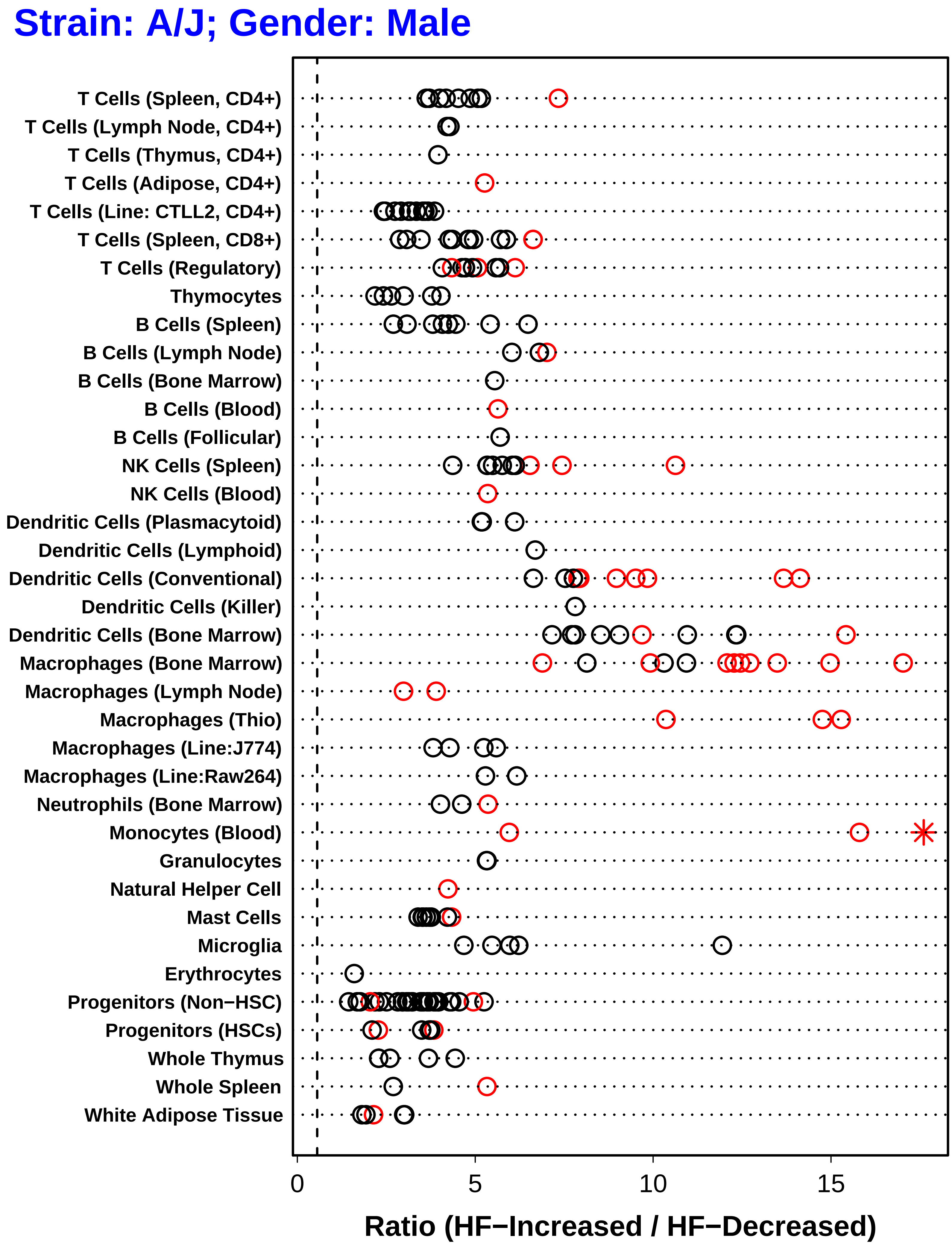
Strain: A/J; Gender: Male
T Cells (Spleen, CD4+)
T Cells (Lymph Node, CD4+)
T Cells (Thymus, CD4+)
T Cells (Adipose, CD4+)
T Cells (Line: CTLL2, CD4+)
T Cells (Spleen, CD8+)
T Cells (Regulatory)
Thymocytes
B Cells (Spleen)
B Cells (Lymph Node)
B Cells (Bone Marrow)
B Cells (Blood)
B Cells (Follicular)
NK Cells (Spleen)
NK Cells (Blood)
Dendritic Cells (Plasmacytoid)
Dendritic Cells (Lymphoid)
Dendritic Cells (Conventional)
Dendritic Cells (Killer)
Dendritic Cells (Bone Marrow)
Macrophages (Bone Marrow)
Macrophages (Lymph Node)
Macrophages (Thio)
Macrophages (Line:J774)
Macrophages (Line:Raw264)
Neutrophils (Bone Marrow)
Monocytes (Blood)
Granulocytes
Natural Helper Cell
Mast Cells
Microglia
Erythrocytes
Progenitors (Non−HSC)
Progenitors (HSCs)
Whole Thymus
Whole Spleen
White Adipose Tissue
0
5
10
15
Ratio (HF−Increased / HF−Decreased)

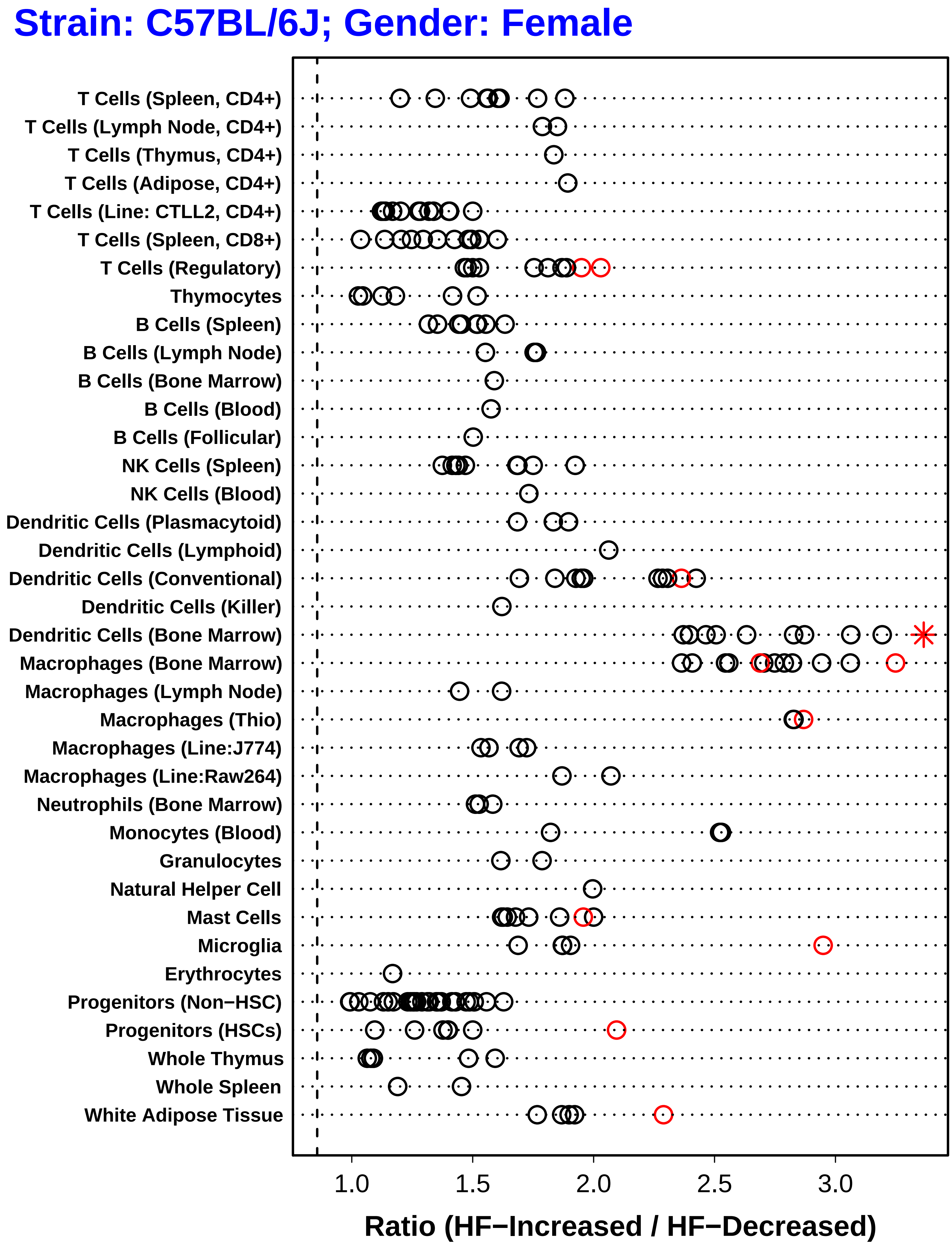
Strain: C57BL/6J; Gender: Female
T Cells (Spleen, CD4+)
T Cells (Lymph Node, CD4+)
T Cells (Thymus, CD4+)
T Cells (Adipose, CD4+)
T Cells (Line: CTLL2, CD4+)
T Cells (Spleen, CD8+)
T Cells (Regulatory)
Thymocytes
B Cells (Spleen)
B Cells (Lymph Node)
B Cells (Bone Marrow)
B Cells (Blood)
B Cells (Follicular)
NK Cells (Spleen)
NK Cells (Blood)
Dendritic Cells (Plasmacytoid)
Dendritic Cells (Lymphoid)
Dendritic Cells (Conventional)
Dendritic Cells (Killer)
Dendritic Cells (Bone Marrow)
Macrophages (Bone Marrow)
Macrophages (Lymph Node)
Macrophages (Thio)
Macrophages (Line:J774)
Macrophages (Line:Raw264)
Neutrophils (Bone Marrow)
Monocytes (Blood)
Granulocytes
Natural Helper Cell
Mast Cells
Microglia
Erythrocytes
Progenitors (Non−HSC)
Progenitors (HSCs)
Whole Thymus
Whole Spleen
White Adipose Tissue
1.0
1.5
2.0
2.5
3.0
Ratio (HF−Increased / HF−Decreased)

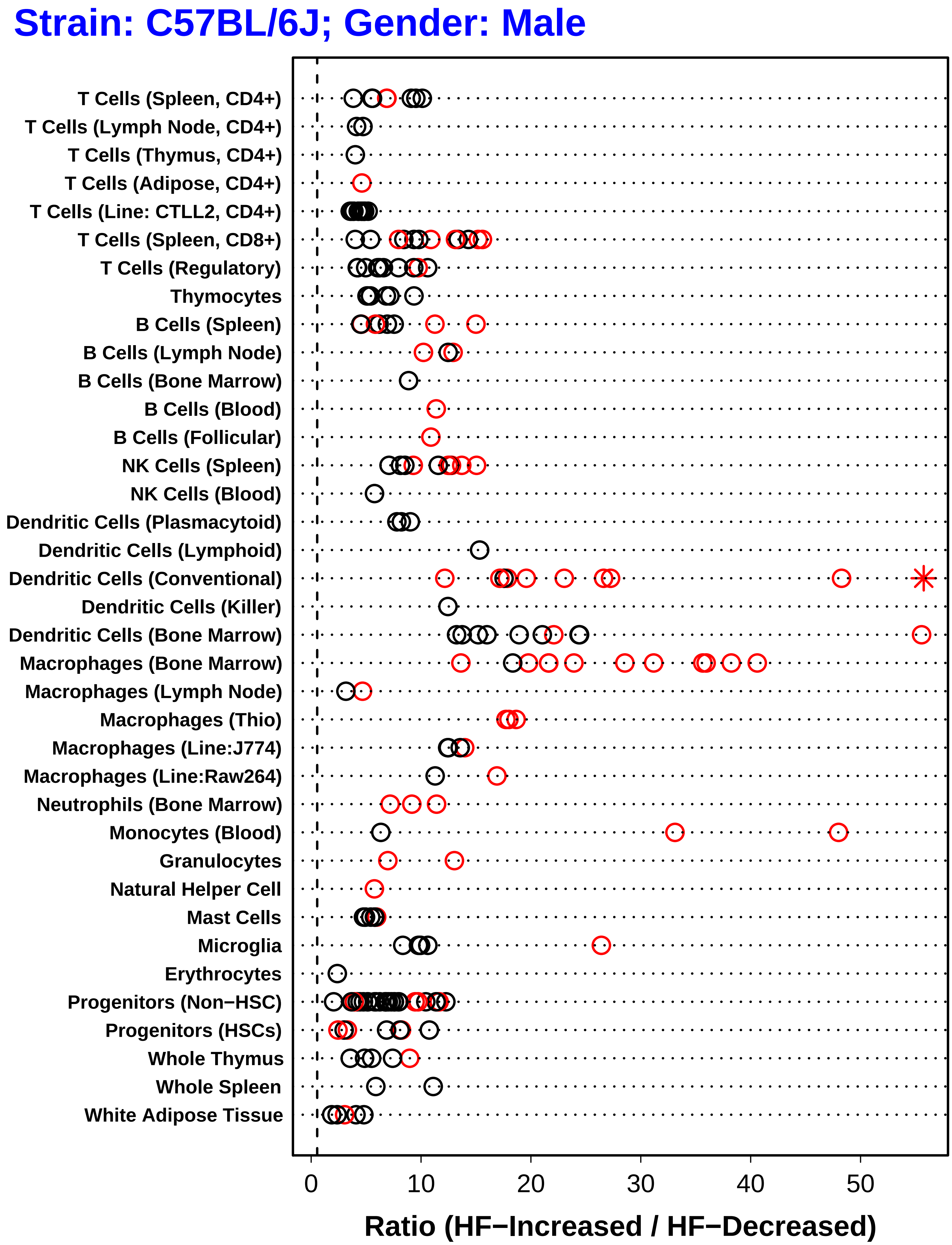
Strain: C57BL/6J; Gender: Male
T Cells (Spleen, CD4+)
T Cells (Lymph Node, CD4+)
T Cells (Thymus, CD4+)
T Cells (Adipose, CD4+)
T Cells (Line: CTLL2, CD4+)
T Cells (Spleen, CD8+)
T Cells (Regulatory)
Thymocytes
B Cells (Spleen)
B Cells (Lymph Node)
B Cells (Bone Marrow)
B Cells (Blood)
B Cells (Follicular)
NK Cells (Spleen)
NK Cells (Blood)
Dendritic Cells (Plasmacytoid)
Dendritic Cells (Lymphoid)
Dendritic Cells (Conventional)
Dendritic Cells (Killer)
Dendritic Cells (Bone Marrow)
Macrophages (Bone Marrow)
Macrophages (Lymph Node)
Macrophages (Thio)
Macrophages (Line:J774)
Macrophages (Line:Raw264)
Neutrophils (Bone Marrow)
Monocytes (Blood)
Granulocytes
Natural Helper Cell
Mast Cells
Microglia
Erythrocytes
Progenitors (Non−HSC)
Progenitors (HSCs)
Whole Thymus
Whole Spleen
White Adipose Tissue
0
10
20
30
40
50
Ratio (HF−Increased / HF−Decreased)

Strain: BALB/cJ; Gender: Female

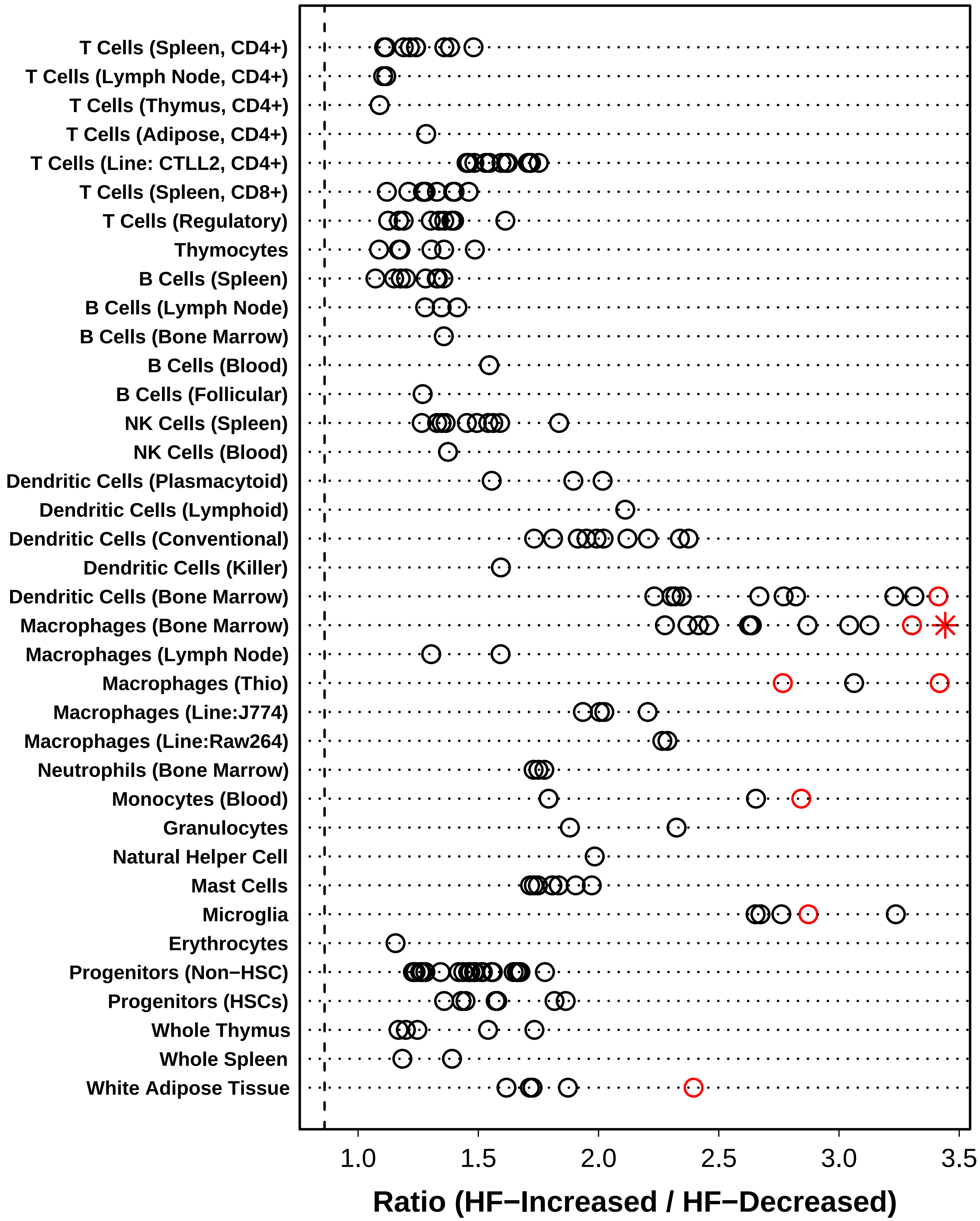

Strain: BALB/cJ; Gender: Male

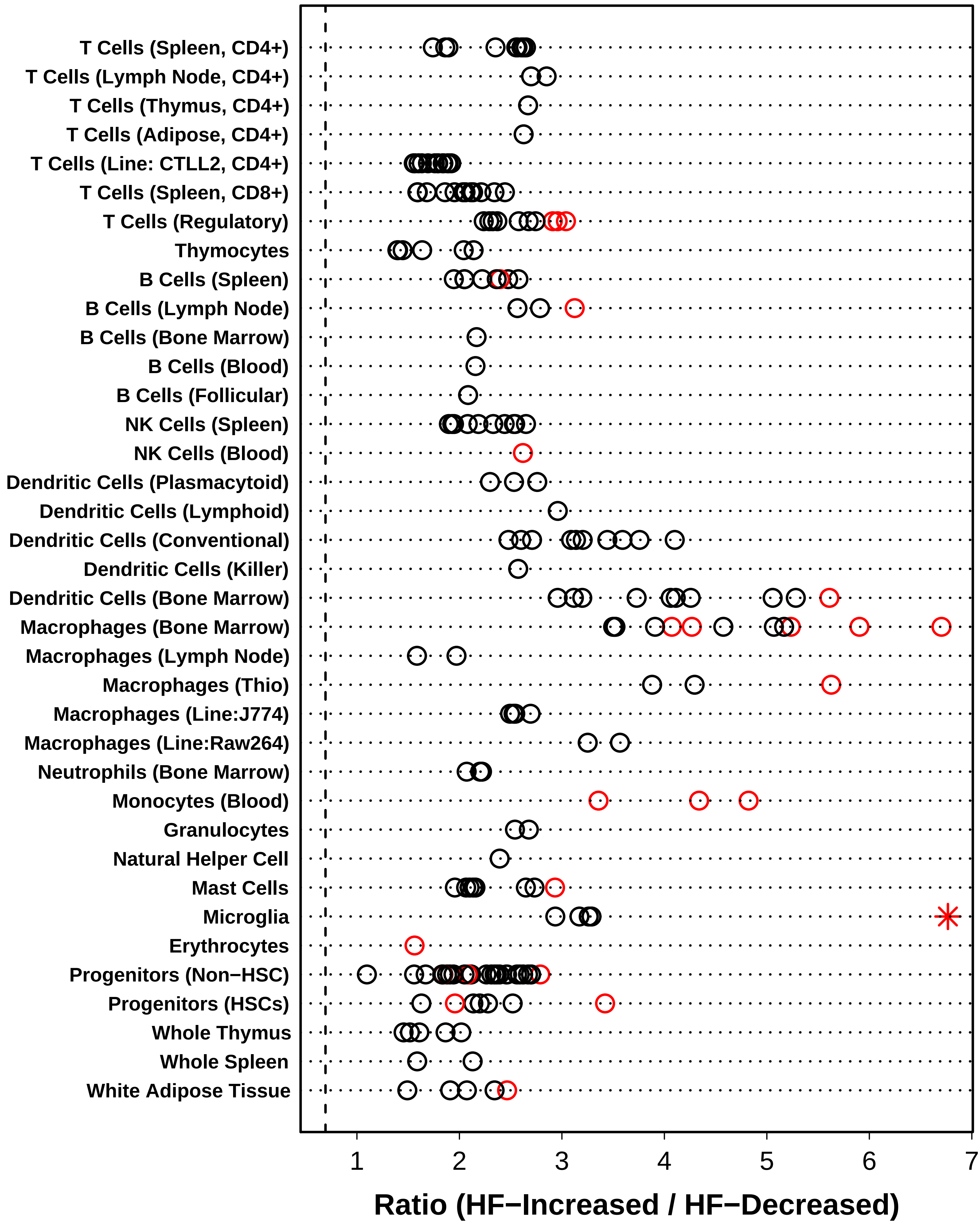

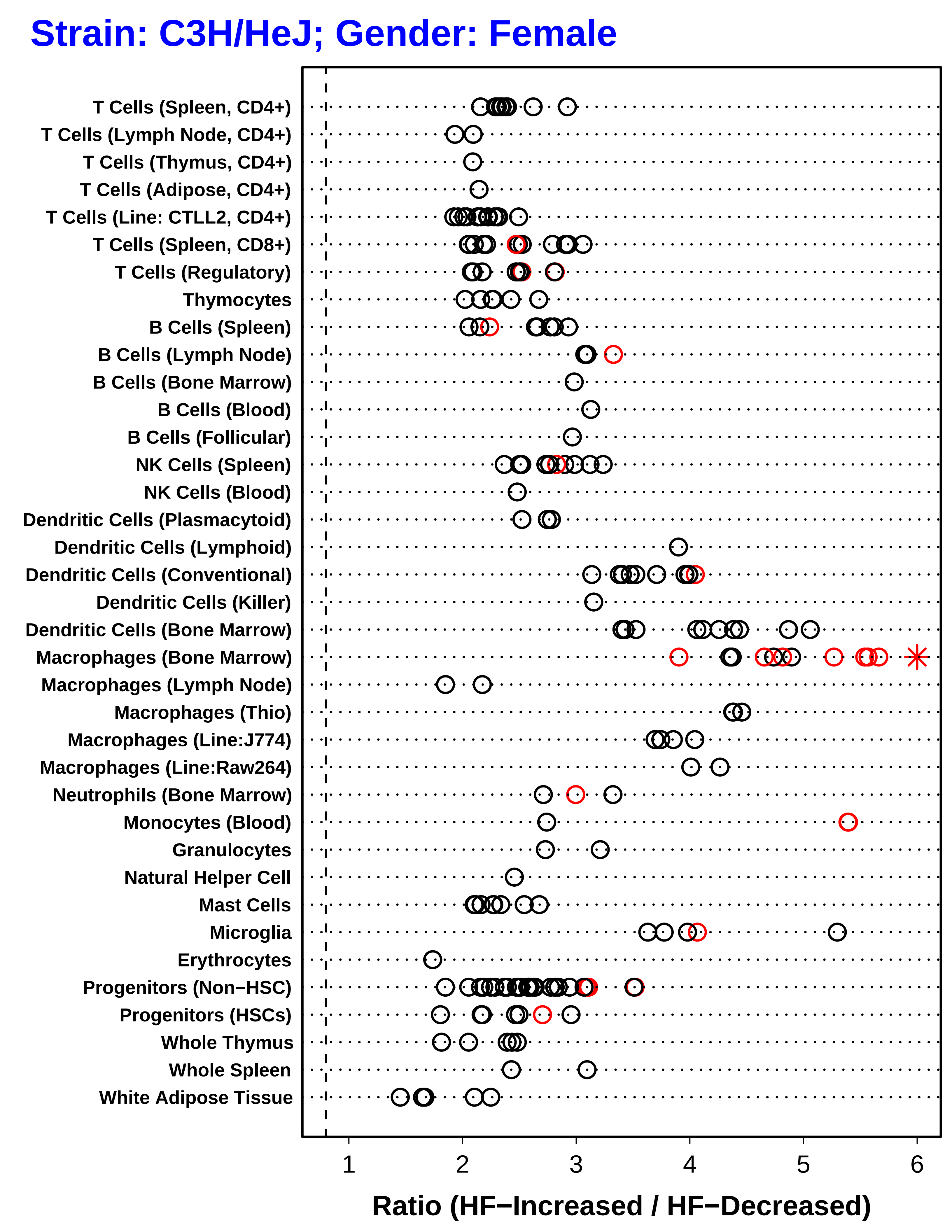
Strain: C3H/HeJ; Gender: Female
T Cells (Spleen, CD4+)
T Cells (Lymph Node, CD4+)
T Cells (Thymus, CD4+)
T Cells (Adipose, CD4+)
T Cells (Line: CTLL2, CD4+)
T Cells (Spleen, CD8+)
T Cells (Regulatory)
Thymocytes
B Cells (Spleen)
B Cells (Lymph Node)
B Cells (Bone Marrow)
B Cells (Blood)
B Cells (Follicular)
NK Cells (Spleen)
NK Cells (Blood)
Dendritic Cells (Plasmacytoid)
Dendritic Cells (Lymphoid)
Dendritic Cells (Conventional)
Dendritic Cells (Killer)
Dendritic Cells (Bone Marrow)
Macrophages (Bone Marrow)
Macrophages (Lymph Node)
Macrophages (Thio)
Macrophages (Line:J774)
Macrophages (Line:Raw264)
Neutrophils (Bone Marrow)
Monocytes (Blood)
Granulocytes
Natural Helper Cell
Mast Cells
Microglia
Erythrocytes
Progenitors (Non−HSC)
Progenitors (HSCs)
Whole Thymus
Whole Spleen
White Adipose Tissue
1
2
3
4
5
6
Ratio (HF−Increased / HF−Decreased)

Strain: C3H/HeJ; Gender: Male

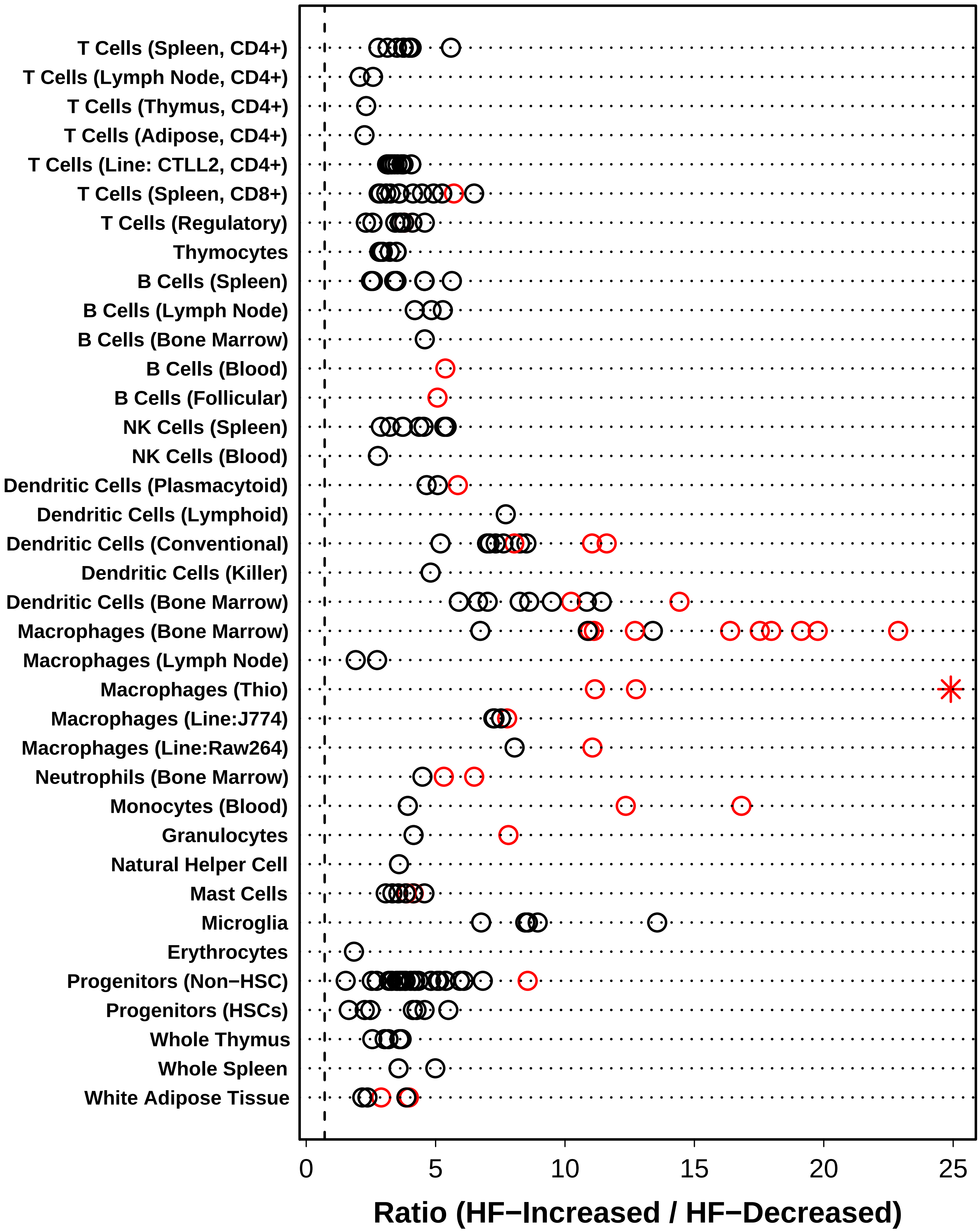

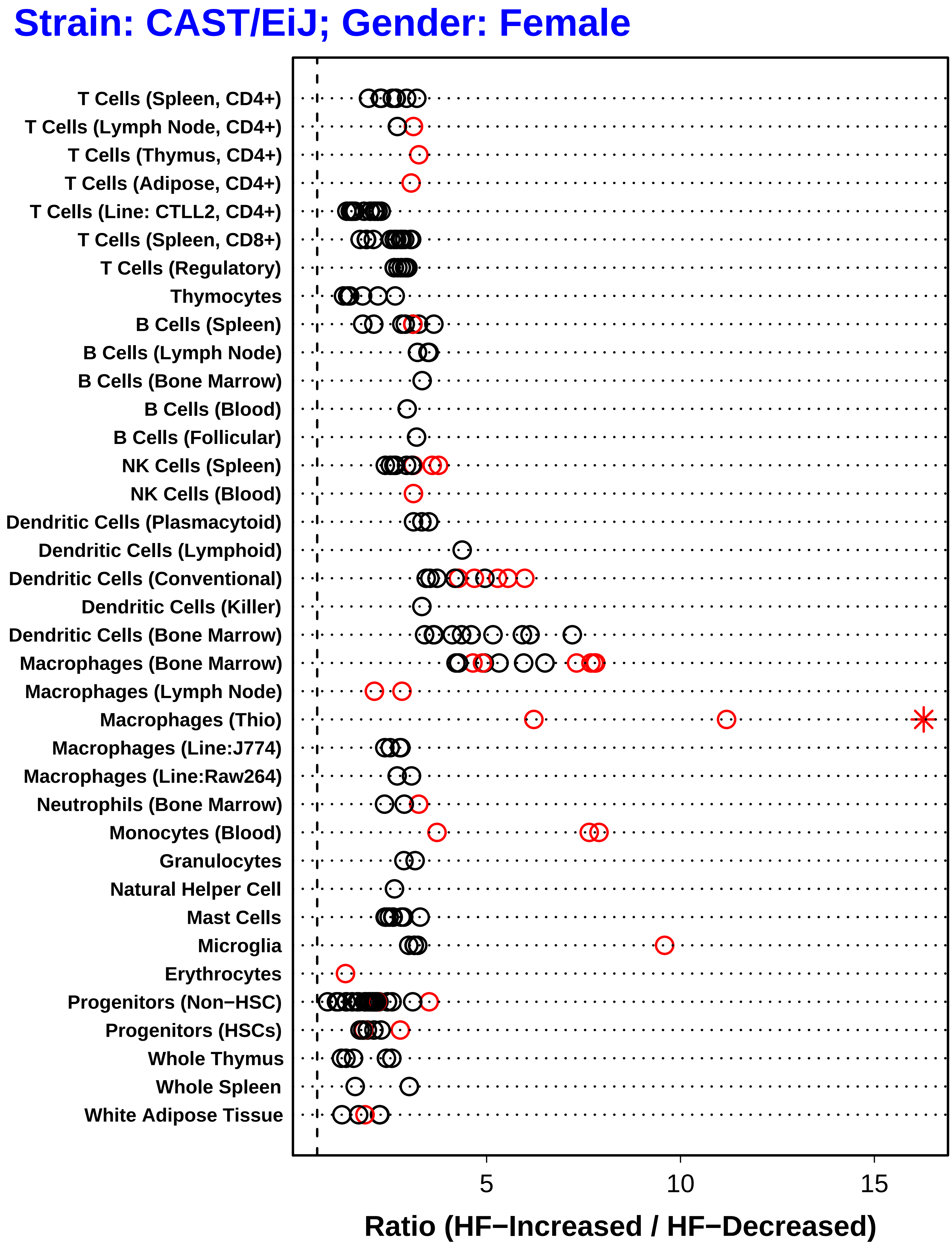

Strain: CAST/EiJ; Gender: Female
T Cells (Spleen, CD4+)
T Cells (Lymph Node, CD4+)
T Cells (Thymus, CD4+)
T Cells (Adipose, CD4+)
T Cells (Line: CTLL2, CD4+)
T Cells (Spleen, CD8+)
T Cells (Regulatory)
Thymocytes
B Cells (Spleen)
B Cells (Lymph Node)
B Cells (Bone Marrow)
B Cells (Blood)
B Cells (Follicular)
NK Cells (Spleen)
NK Cells (Blood)
Dendritic Cells (Plasmacytoid)
Dendritic Cells (Lymphoid)
Dendritic Cells (Conventional)
Dendritic Cells (Killer)
Dendritic Cells (Bone Marrow)
Macrophages (Bone Marrow)
Macrophages (Lymph Node)
Macrophages (Thio)
Macrophages (Line:J774)
Macrophages (Line:Raw264)
Neutrophils (Bone Marrow)
Monocytes (Blood)
Granulocytes
Natural Helper Cell
Mast Cells
Microglia
Erythrocytes
Progenitors (Non−HSC)
Progenitors (HSCs)
Whole Thymus
Whole Spleen
White Adipose Tissue
5
10
15
Ratio (HF−Increased / HF−Decreased)

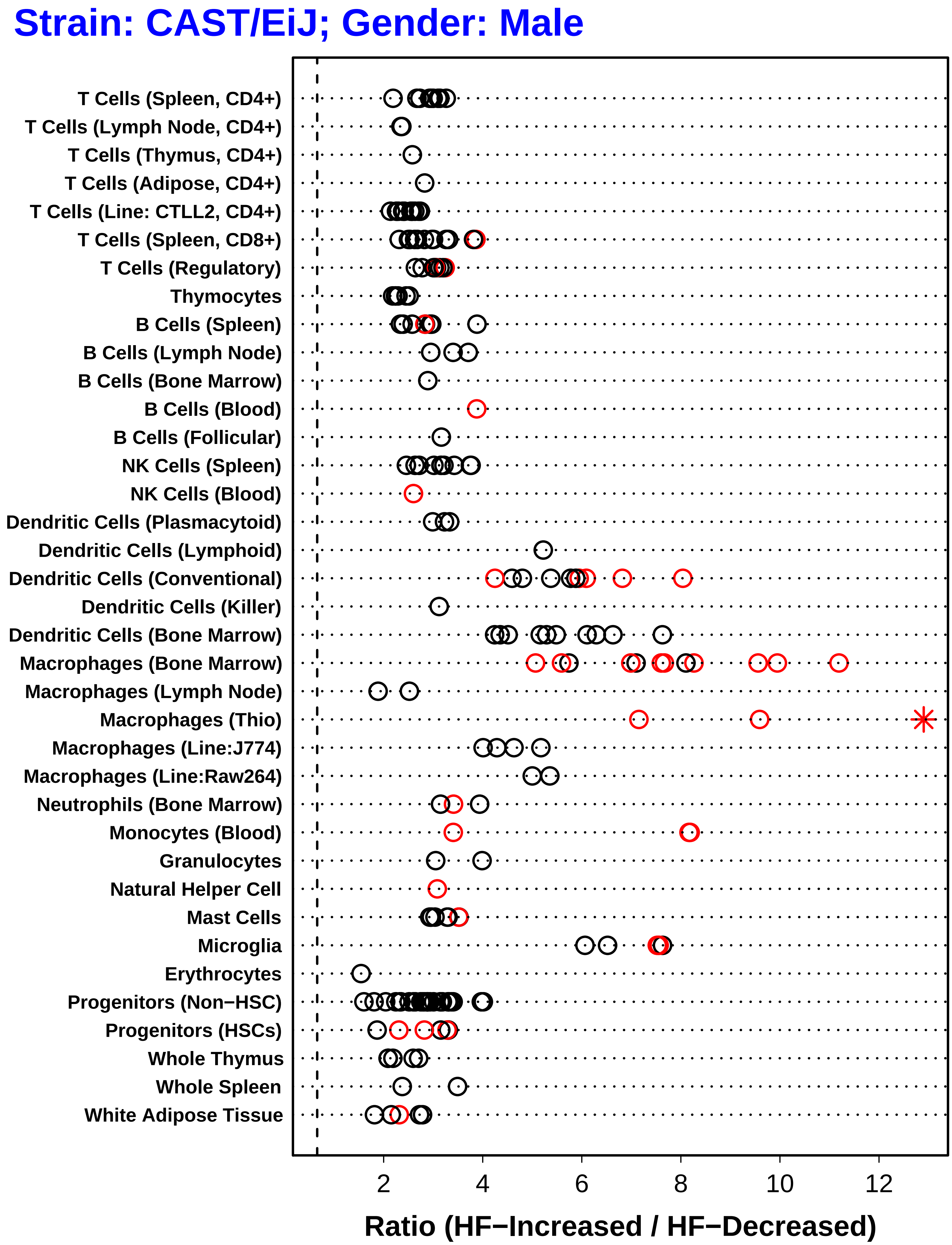
Strain: CAST/EiJ; Gender: Male
T Cells (Spleen, CD4+)
T Cells (Lymph Node, CD4+)
T Cells (Thymus, CD4+)
T Cells (Adipose, CD4+)
T Cells (Line: CTLL2, CD4+)
T Cells (Spleen, CD8+)
T Cells (Regulatory)
Thymocytes
B Cells (Spleen)
B Cells (Lymph Node)
B Cells (Bone Marrow)
B Cells (Blood)
B Cells (Follicular)
NK Cells (Spleen)
NK Cells (Blood)
Dendritic Cells (Plasmacytoid)
Dendritic Cells (Lymphoid)
Dendritic Cells (Conventional)
Dendritic Cells (Killer)
Dendritic Cells (Bone Marrow)
Macrophages (Bone Marrow)
Macrophages (Lymph Node)
Macrophages (Thio)
Macrophages (Line:J774)
Macrophages (Line:Raw264)
Neutrophils (Bone Marrow)
Monocytes (Blood)
Granulocytes
Natural Helper Cell
Mast Cells
Microglia
Erythrocytes
Progenitors (Non−HSC)
Progenitors (HSCs)
Whole Thymus
Whole Spleen
White Adipose Tissue
2
4
6
8
10
12
Ratio (HF−Increased / HF−Decreased)

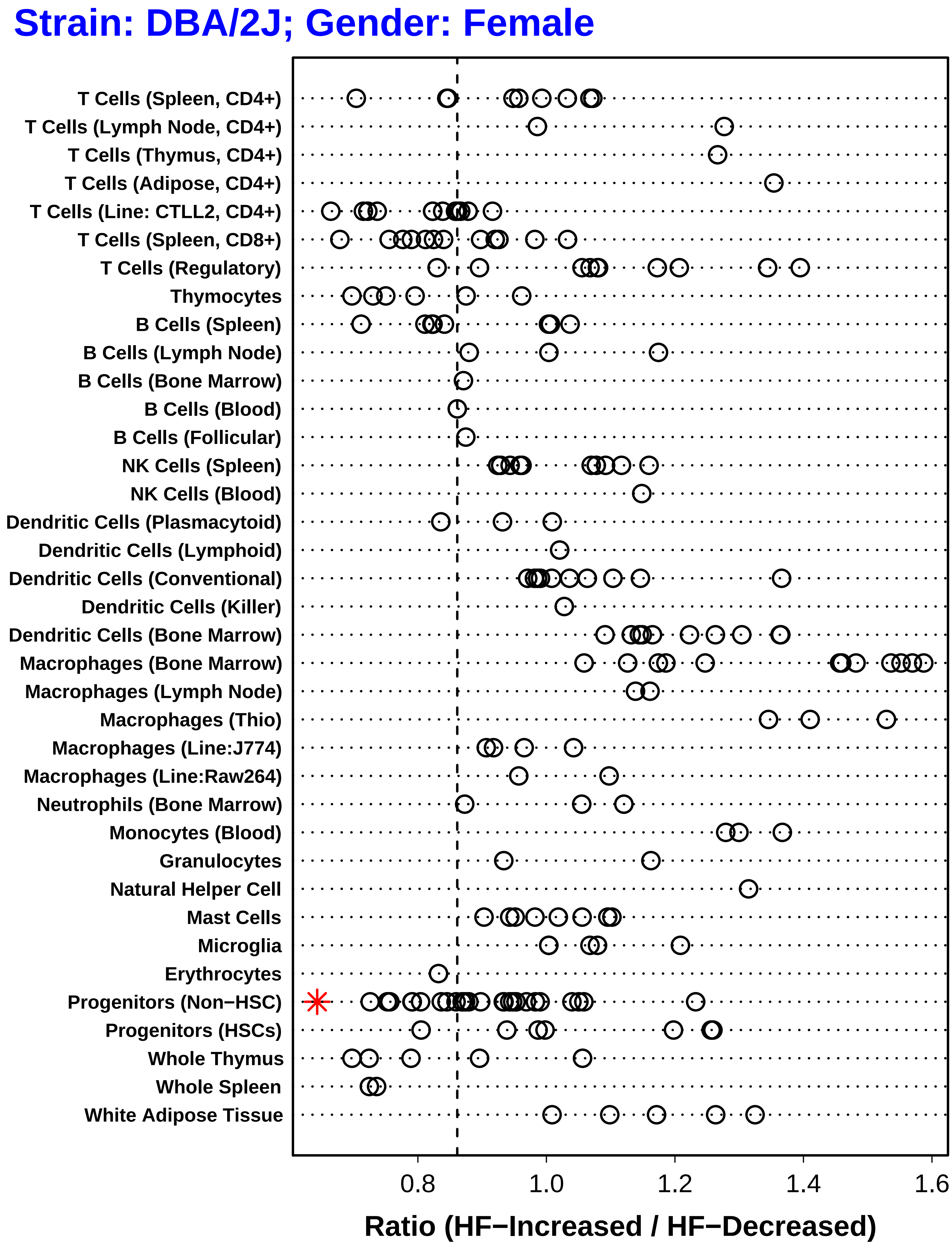

Strain: DBA/2J; Gender: Female
T Cells (Spleen, CD4+)
T Cells (Lymph Node, CD4+)
T Cells (Thymus, CD4+)
T Cells (Adipose, CD4+)
T Cells (Line: CTLL2, CD4+)
T Cells (Spleen, CD8+)
T Cells (Regulatory)
Thymocytes
B Cells (Spleen)
B Cells (Lymph Node)
B Cells (Bone Marrow)
B Cells (Blood)
B Cells (Follicular)
NK Cells (Spleen)
NK Cells (Blood)
Dendritic Cells (Plasmacytoid)
Dendritic Cells (Lymphoid)
Dendritic Cells (Conventional)
Dendritic Cells (Killer)
Dendritic Cells (Bone Marrow)
Macrophages (Bone Marrow)
Macrophages (Lymph Node)
Macrophages (Thio)
Macrophages (Line:J774)
Macrophages (Line:Raw264)
Neutrophils (Bone Marrow)
Monocytes (Blood)
Granulocytes
Natural Helper Cell
Mast Cells
Microglia
Erythrocytes
Progenitors (Non−HSC)
Progenitors (HSCs)
Whole Thymus
Whole Spleen
White Adipose Tissue
0.8
1.0
1.2
1.4
1.6
Ratio (HF−Increased / HF−Decreased)

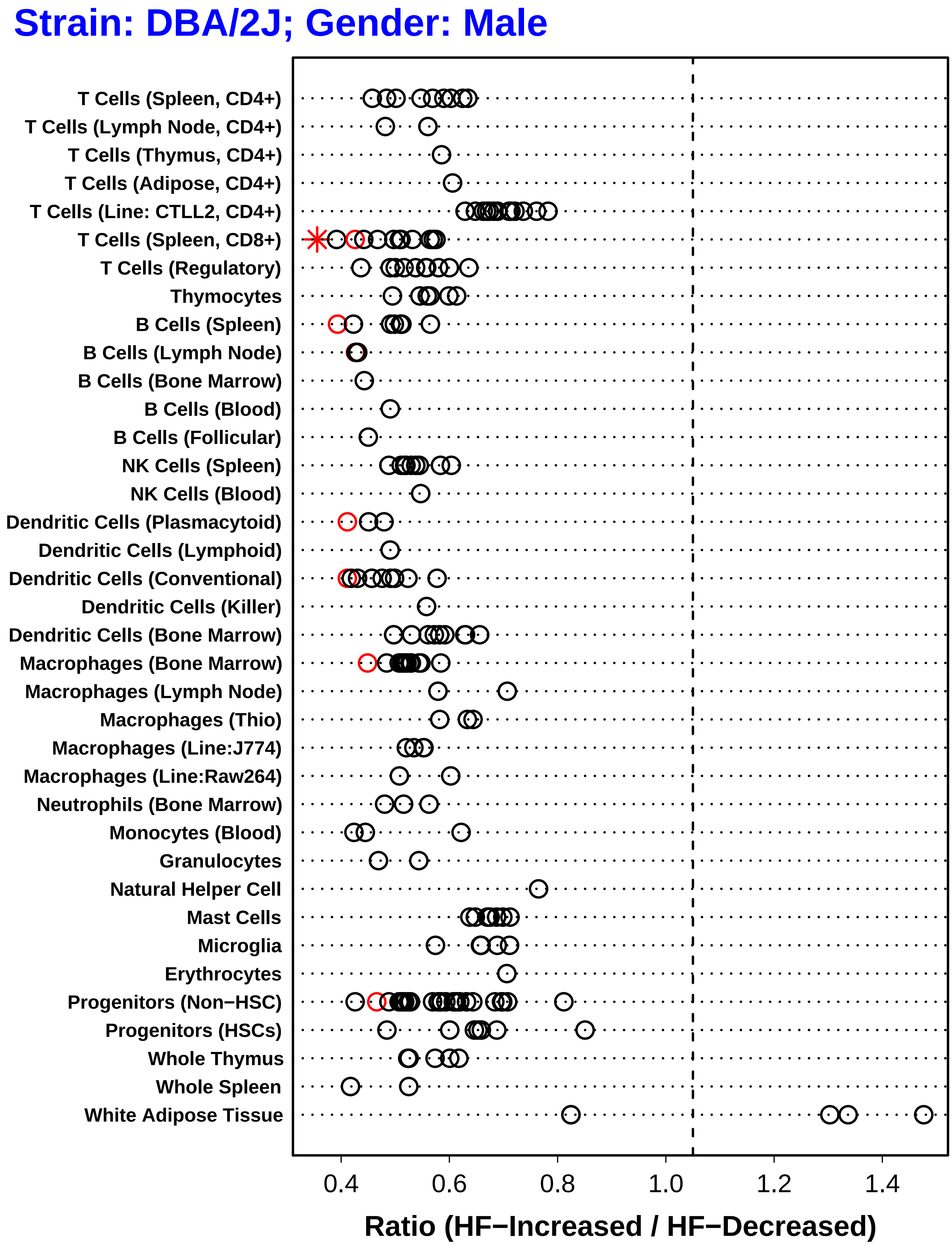
Strain: DBA/2J; Gender: Male
T Cells (Spleen, CD4+)
T Cells (Lymph Node, CD4+)
T Cells (Thymus, CD4+)
T Cells (Adipose, CD4+)
T Cells (Line: CTLL2, CD4+)
T Cells (Spleen, CD8+)
T Cells (Regulatory)
Thymocytes
B Cells (Spleen)
B Cells (Lymph Node)
B Cells (Bone Marrow)
B Cells (Blood)
B Cells (Follicular)
NK Cells (Spleen)
NK Cells (Blood)
Dendritic Cells (Plasmacytoid)
Dendritic Cells (Lymphoid)
Dendritic Cells (Conventional)
Dendritic Cells (Killer)
Dendritic Cells (Bone Marrow)
Macrophages (Bone Marrow)
Macrophages (Lymph Node)
Macrophages (Thio)
Macrophages (Line:J774)
Macrophages (Line:Raw264)
Neutrophils (Bone Marrow)
Monocytes (Blood)
Granulocytes
Natural Helper Cell
Mast Cells
Microglia
Erythrocytes
Progenitors (Non−HSC)
Progenitors (HSCs)
Whole Thymus
Whole Spleen
White Adipose Tissue
0.4
0.6
0.8
1.0
1.2
1.4
Ratio (HF−Increased / HF−Decreased)

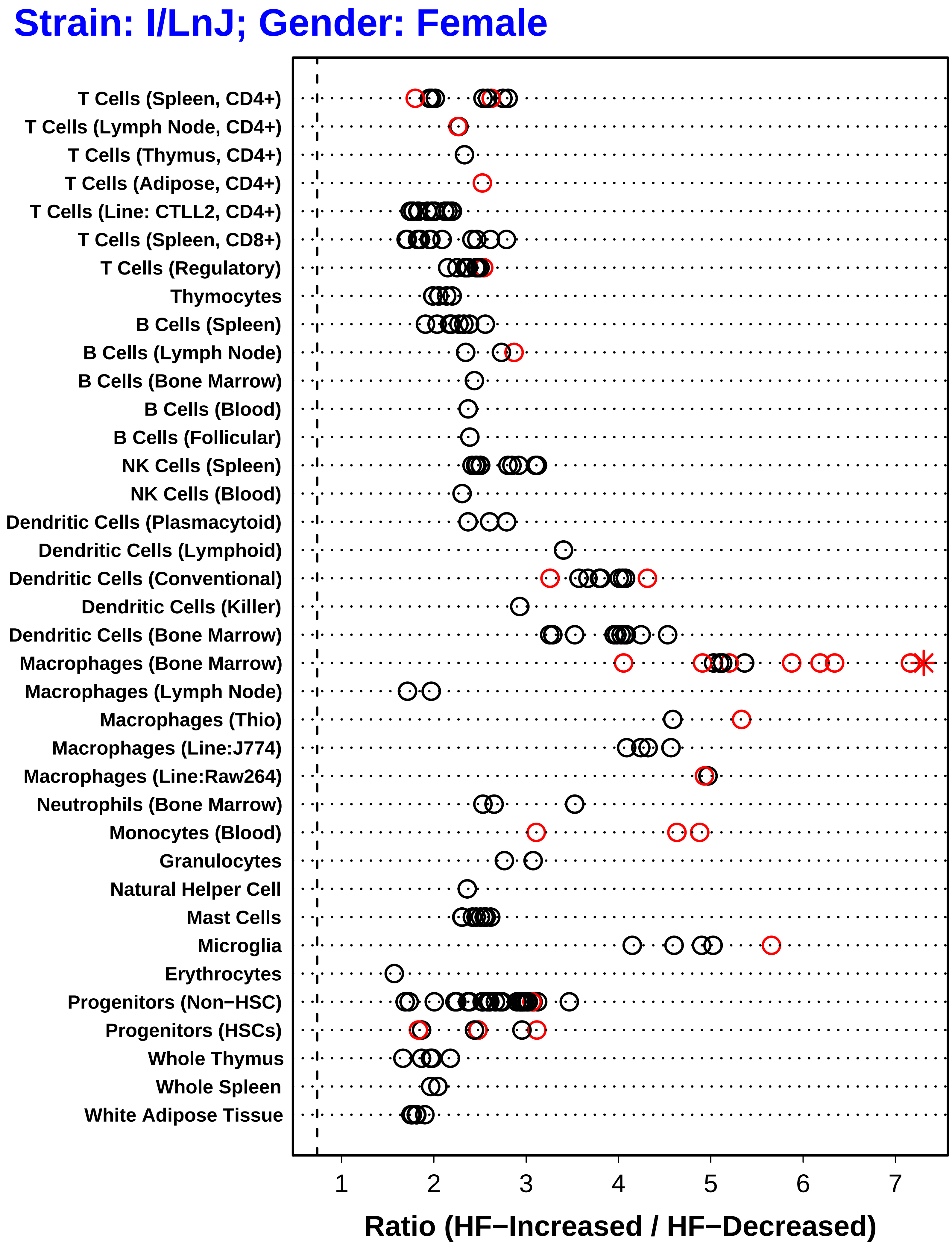

Strain: I/LnJ; Gender: Female
T Cells (Spleen, CD4+)
T Cells (Lymph Node, CD4+)
T Cells (Thymus, CD4+)
T Cells (Adipose, CD4+)
T Cells (Line: CTLL2, CD4+)
T Cells (Spleen, CD8+)
T Cells (Regulatory)
Thymocytes
B Cells (Spleen)
B Cells (Lymph Node)
B Cells (Bone Marrow)
B Cells (Blood)
B Cells (Follicular)
NK Cells (Spleen)
NK Cells (Blood)
Dendritic Cells (Plasmacytoid)
Dendritic Cells (Lymphoid)
Dendritic Cells (Conventional)
Dendritic Cells (Killer)
Dendritic Cells (Bone Marrow)
Macrophages (Bone Marrow)
Macrophages (Lymph Node)
Macrophages (Thio)
Macrophages (Line:J774)
Macrophages (Line:Raw264)
Neutrophils (Bone Marrow)
Monocytes (Blood)
Granulocytes
Natural Helper Cell
Mast Cells
Microglia
Erythrocytes
Progenitors (Non−HSC)
Progenitors (HSCs)
Whole Thymus
Whole Spleen
White Adipose Tissue
1
2
3
4
5
6
7
Ratio (HF−Increased / HF−Decreased)

Strain: I/LnJ; Gender: Male

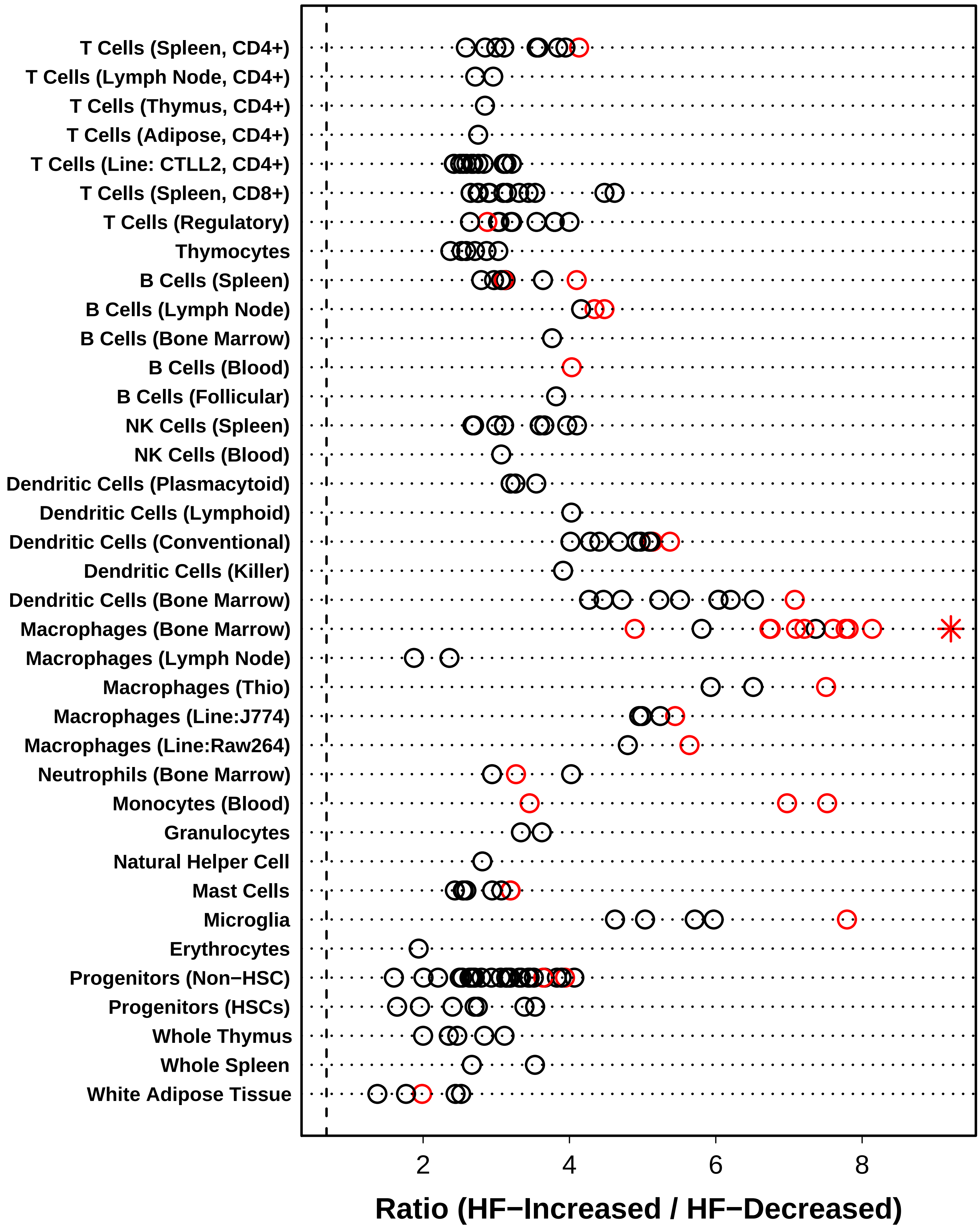

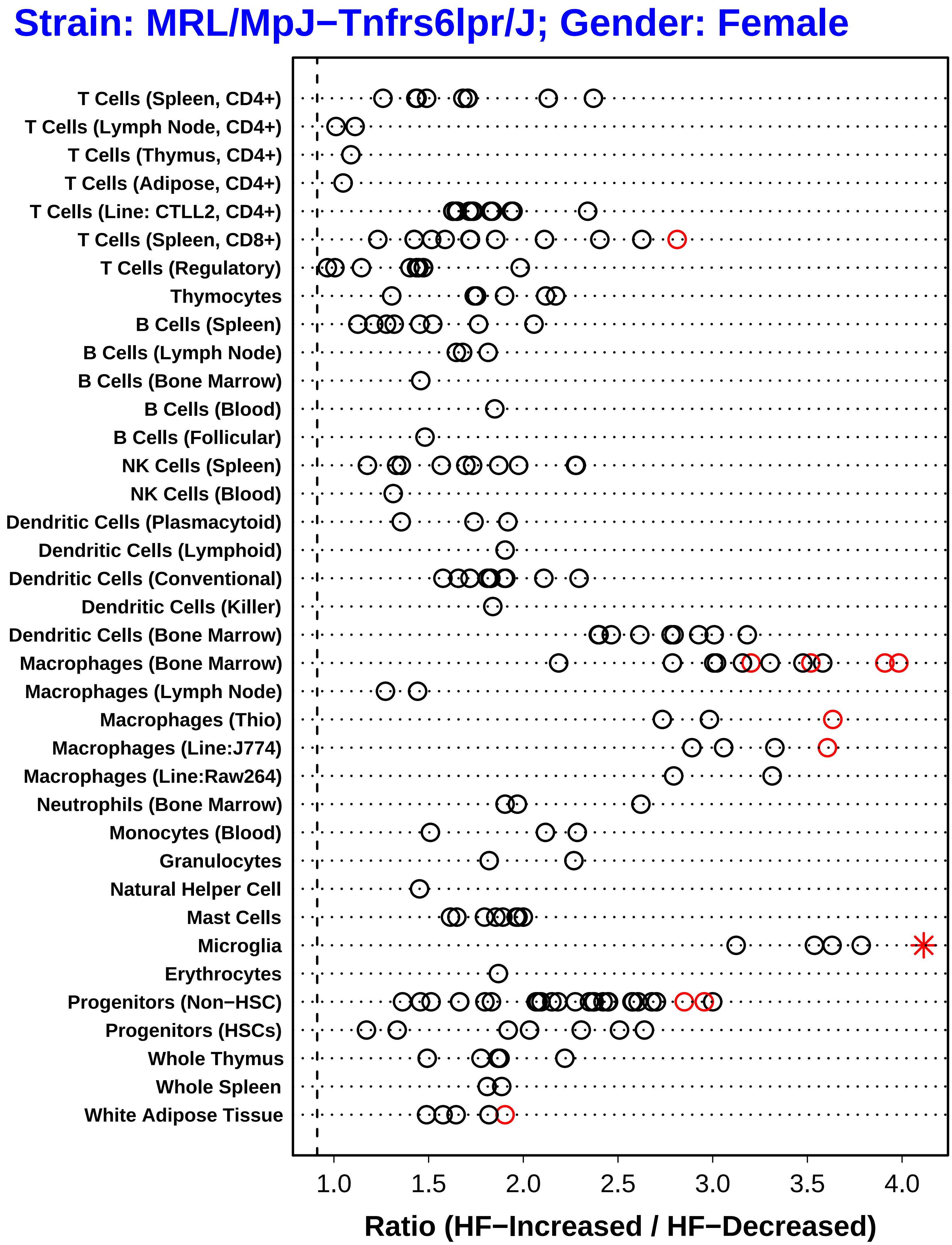
Strain: MRL/MpJ−Tnfrs6lpr/J; Gender: Female
T Cells (Spleen, CD4+)
T Cells (Lymph Node, CD4+)
T Cells (Thymus, CD4+)
T Cells (Adipose, CD4+)
T Cells (Line: CTLL2, CD4+)
T Cells (Spleen, CD8+)
T Cells (Regulatory)
Thymocytes
B Cells (Spleen)
B Cells (Lymph Node)
B Cells (Bone Marrow)
B Cells (Blood)
B Cells (Follicular)
NK Cells (Spleen)
NK Cells (Blood)
Dendritic Cells (Plasmacytoid)
Dendritic Cells (Lymphoid)
Dendritic Cells (Conventional)
Dendritic Cells (Killer)
Dendritic Cells (Bone Marrow)
Macrophages (Bone Marrow)
Macrophages (Lymph Node)
Macrophages (Thio)
Macrophages (Line:J774)
Macrophages (Line:Raw264)
Neutrophils (Bone Marrow)
Monocytes (Blood)
Granulocytes
Natural Helper Cell
Mast Cells
Microglia
Erythrocytes
Progenitors (Non−HSC)
Progenitors (HSCs)
Whole Thymus
Whole Spleen
White Adipose Tissue
1.0
1.5
2.0
2.5
3.0
3.5
4.0
Ratio (HF−Increased / HF−Decreased)

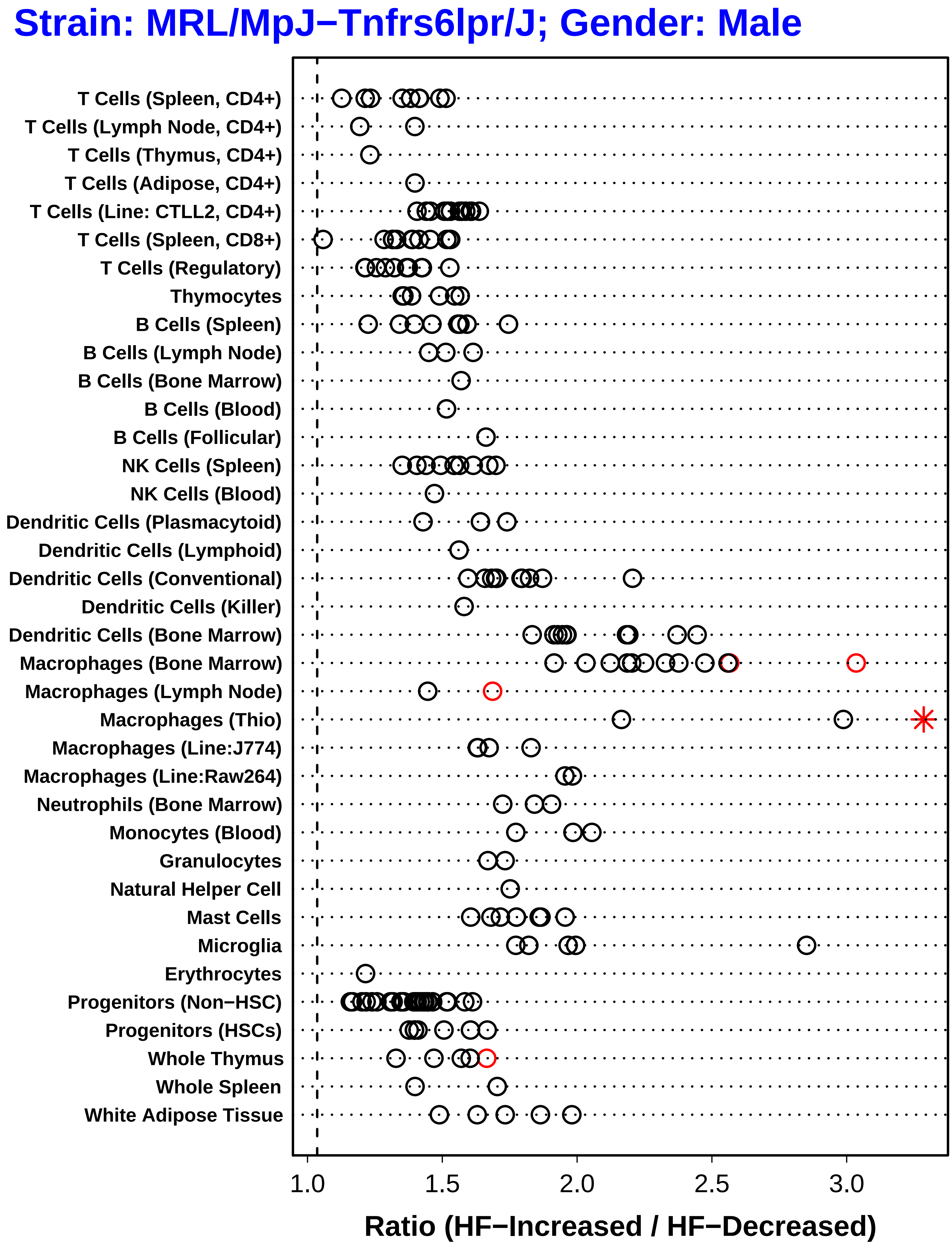
Strain: MRL/MpJ−Tnfrs6lpr/J; Gender: Male
T Cells (Spleen, CD4+)
T Cells (Lymph Node, CD4+)
T Cells (Thymus, CD4+)
T Cells (Adipose, CD4+)
T Cells (Line: CTLL2, CD4+)
T Cells (Spleen, CD8+)
T Cells (Regulatory)
Thymocytes
B Cells (Spleen)
B Cells (Lymph Node)
B Cells (Bone Marrow)
B Cells (Blood)
B Cells (Follicular)
NK Cells (Spleen)
NK Cells (Blood)
Dendritic Cells (Plasmacytoid)
Dendritic Cells (Lymphoid)
Dendritic Cells (Conventional)
Dendritic Cells (Killer)
Dendritic Cells (Bone Marrow)
Macrophages (Bone Marrow)
Macrophages (Lymph Node)
Macrophages (Thio)
Macrophages (Line:J774)
Macrophages (Line:Raw264)
Neutrophils (Bone Marrow)
Monocytes (Blood)
Granulocytes
Natural Helper Cell
Mast Cells
Microglia
Erythrocytes
Progenitors (Non−HSC)
Progenitors (HSCs)
Whole Thymus
Whole Spleen
White Adipose Tissue
1.0
1.5
2.0
2.5
3.0
Ratio (HF−Increased / HF−Decreased)

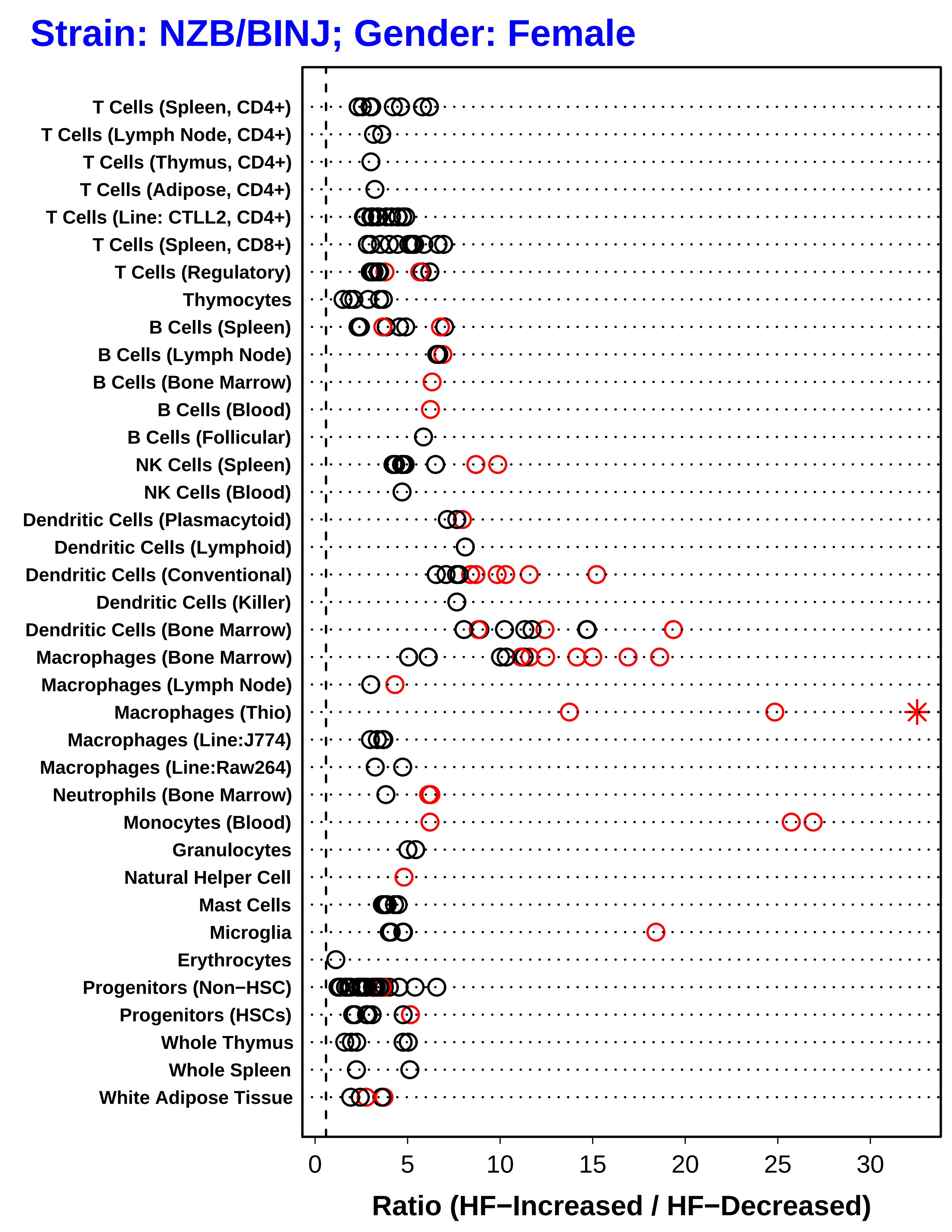

Strain: NZB/BINJ; Gender: Female
T Cells (Spleen, CD4+)
T Cells (Lymph Node, CD4+)
T Cells (Thymus, CD4+)
T Cells (Adipose, CD4+)
T Cells (Line: CTLL2, CD4+)
T Cells (Spleen, CD8+)
T Cells (Regulatory)
Thymocytes
B Cells (Spleen)
B Cells (Lymph Node)
B Cells (Bone Marrow)
B Cells (Blood)
B Cells (Follicular)
NK Cells (Spleen)
NK Cells (Blood)
Dendritic Cells (Plasmacytoid)
Dendritic Cells (Lymphoid)
Dendritic Cells (Conventional)
Dendritic Cells (Killer)
Dendritic Cells (Bone Marrow)
Macrophages (Bone Marrow)
Macrophages (Lymph Node)
Macrophages (Thio)
Macrophages (Line:J774)
Macrophages (Line:Raw264)
Neutrophils (Bone Marrow)
Monocytes (Blood)
Granulocytes
Natural Helper Cell
Mast Cells
Microglia
Erythrocytes
Progenitors (Non−HSC)
Progenitors (HSCs)
Whole Thymus
Whole Spleen
White Adipose Tissue
0
5
10
15
20
25
30
Ratio (HF−Increased / HF−Decreased)

Strain: NZB/BINJ; Gender: Male

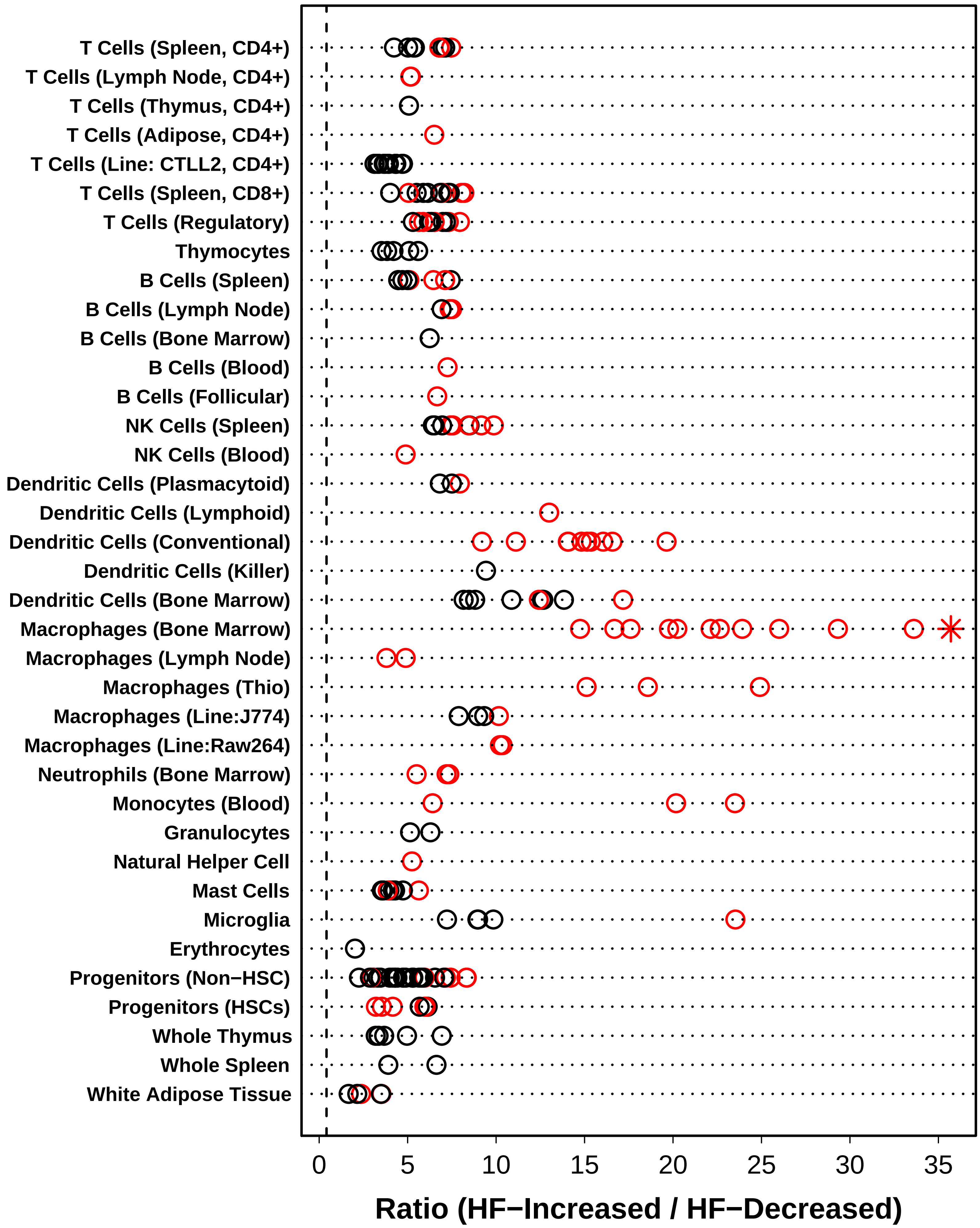

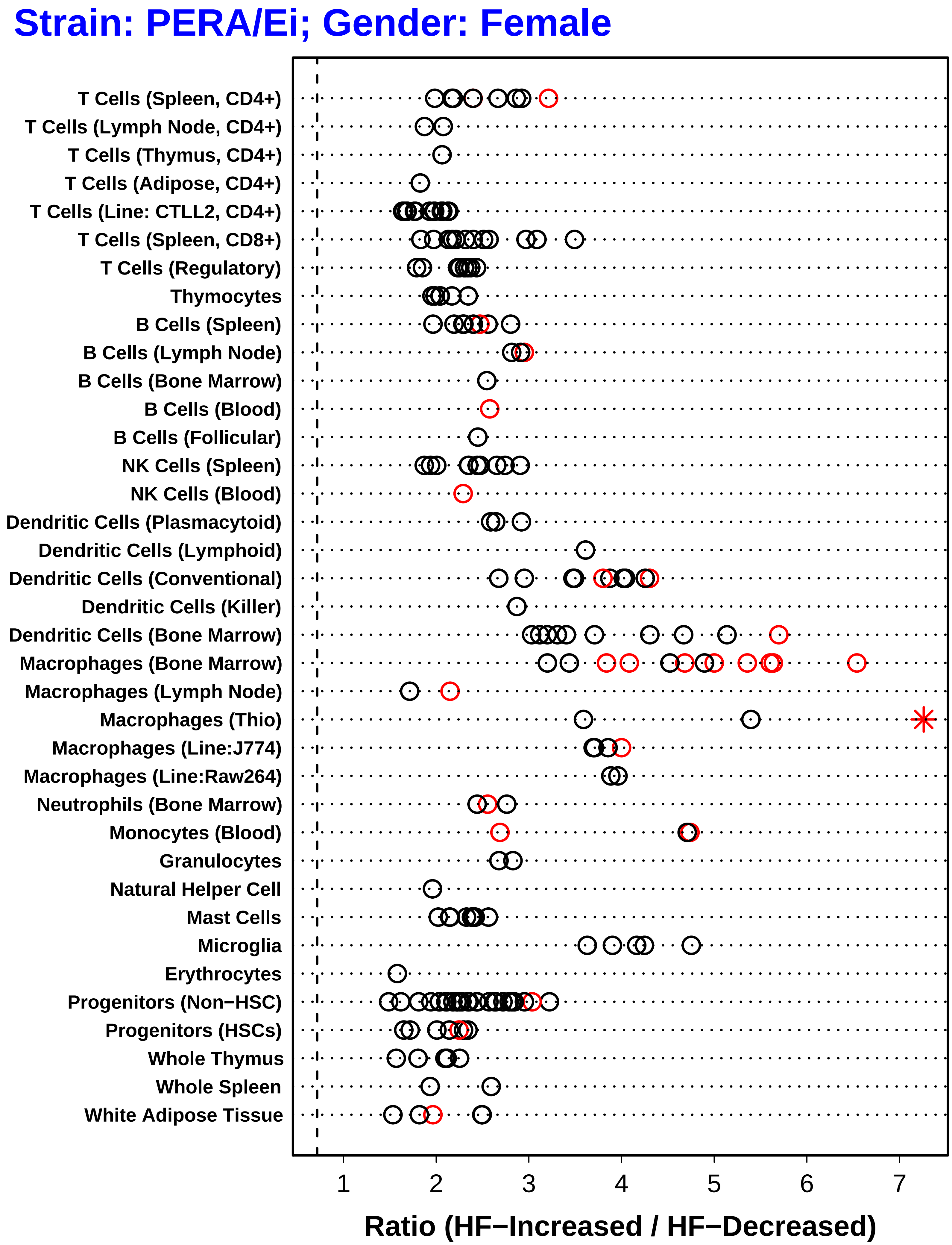
Strain: PERA/Ei; Gender: Female
T Cells (Spleen, CD4+)
T Cells (Lymph Node, CD4+)
T Cells (Thymus, CD4+)
T Cells (Adipose, CD4+)
T Cells (Line: CTLL2, CD4+)
T Cells (Spleen, CD8+)
T Cells (Regulatory)
Thymocytes
B Cells (Spleen)
B Cells (Lymph Node)
B Cells (Bone Marrow)
B Cells (Blood)
B Cells (Follicular)
NK Cells (Spleen)
NK Cells (Blood)
Dendritic Cells (Plasmacytoid)
Dendritic Cells (Lymphoid)
Dendritic Cells (Conventional)
Dendritic Cells (Killer)
Dendritic Cells (Bone Marrow)
Macrophages (Bone Marrow)
Macrophages (Lymph Node)
Macrophages (Thio)
Macrophages (Line:J774)
Macrophages (Line:Raw264)
Neutrophils (Bone Marrow)
Monocytes (Blood)
Granulocytes
Natural Helper Cell
Mast Cells
Microglia
Erythrocytes
Progenitors (Non−HSC)
Progenitors (HSCs)
Whole Thymus
Whole Spleen
White Adipose Tissue
1
2
3
4
5
6
7
Ratio (HF−Increased / HF−Decreased)

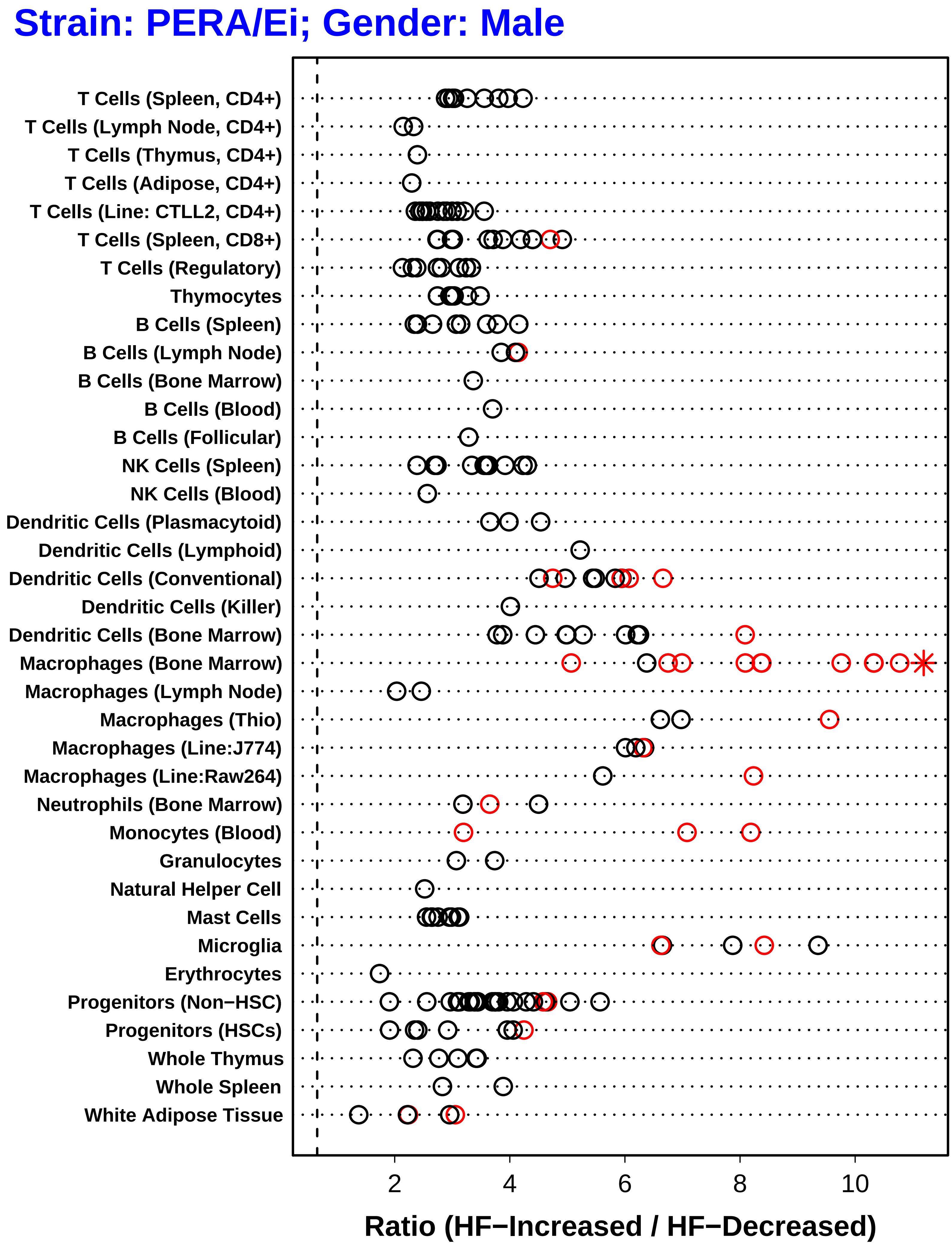

Strain: PERA/Ei; Gender: Male
T Cells (Spleen, CD4+)
T Cells (Lymph Node, CD4+)
T Cells (Thymus, CD4+)
T Cells (Adipose, CD4+)
T Cells (Line: CTLL2, CD4+)
T Cells (Spleen, CD8+)
T Cells (Regulatory)
Thymocytes
B Cells (Spleen)
B Cells (Lymph Node)
B Cells (Bone Marrow)
B Cells (Blood)
B Cells (Follicular)
NK Cells (Spleen)
NK Cells (Blood)
Dendritic Cells (Plasmacytoid)
Dendritic Cells (Lymphoid)
Dendritic Cells (Conventional)
Dendritic Cells (Killer)
Dendritic Cells (Bone Marrow)
Macrophages (Bone Marrow)
Macrophages (Lymph Node)
Macrophages (Thio)
Macrophages (Line:J774)
Macrophages (Line:Raw264)
Neutrophils (Bone Marrow)
Monocytes (Blood)
Granulocytes
Natural Helper Cell
Mast Cells
Microglia
Erythrocytes
Progenitors (Non−HSC)
Progenitors (HSCs)
Whole Thymus
Whole Spleen
White Adipose Tissue
2
4
6
8
10
Ratio (HF−Increased / HF−Decreased)

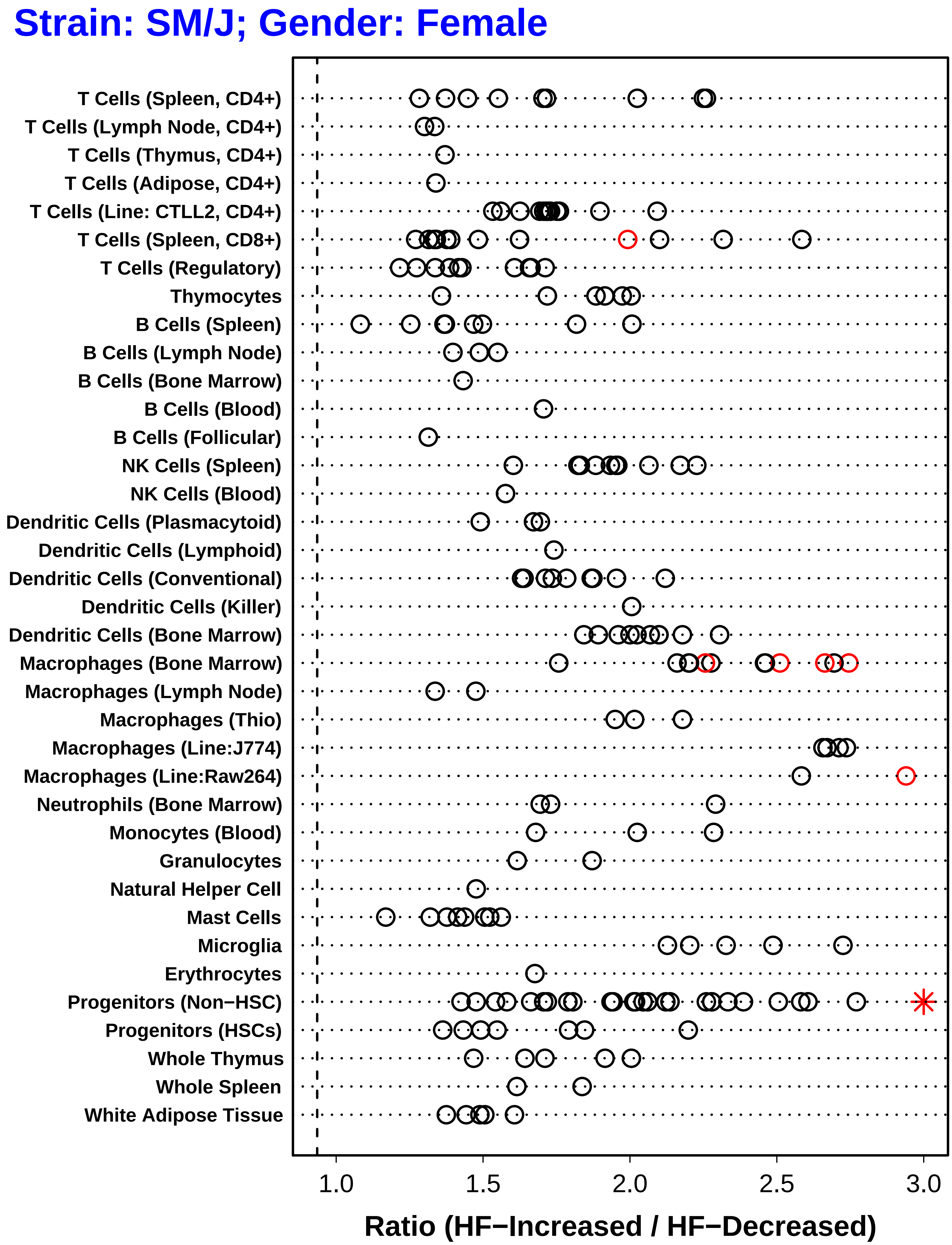
Strain: SM/J; Gender: Female
T Cells (Spleen, CD4+)
T Cells (Lymph Node, CD4+)
T Cells (Thymus, CD4+)
T Cells (Adipose, CD4+)
T Cells (Line: CTLL2, CD4+)
T Cells (Spleen, CD8+)
T Cells (Regulatory)
Thymocytes
B Cells (Spleen)
B Cells (Lymph Node)
B Cells (Bone Marrow)
B Cells (Blood)
B Cells (Follicular)
NK Cells (Spleen)
NK Cells (Blood)
Dendritic Cells (Plasmacytoid)
Dendritic Cells (Lymphoid)
Dendritic Cells (Conventional)
Dendritic Cells (Killer)
Dendritic Cells (Bone Marrow)
Macrophages (Bone Marrow)
Macrophages (Lymph Node)
Macrophages (Thio)
Macrophages (Line:J774)
Macrophages (Line:Raw264)
Neutrophils (Bone Marrow)
Monocytes (Blood)
Granulocytes
Natural Helper Cell
Mast Cells
Microglia
Erythrocytes
Progenitors (Non−HSC)
Progenitors (HSCs)
Whole Thymus
Whole Spleen
White Adipose Tissue
1.0
1.5
2.0
2.5
3.0
Ratio (HF−Increased / HF−Decreased)

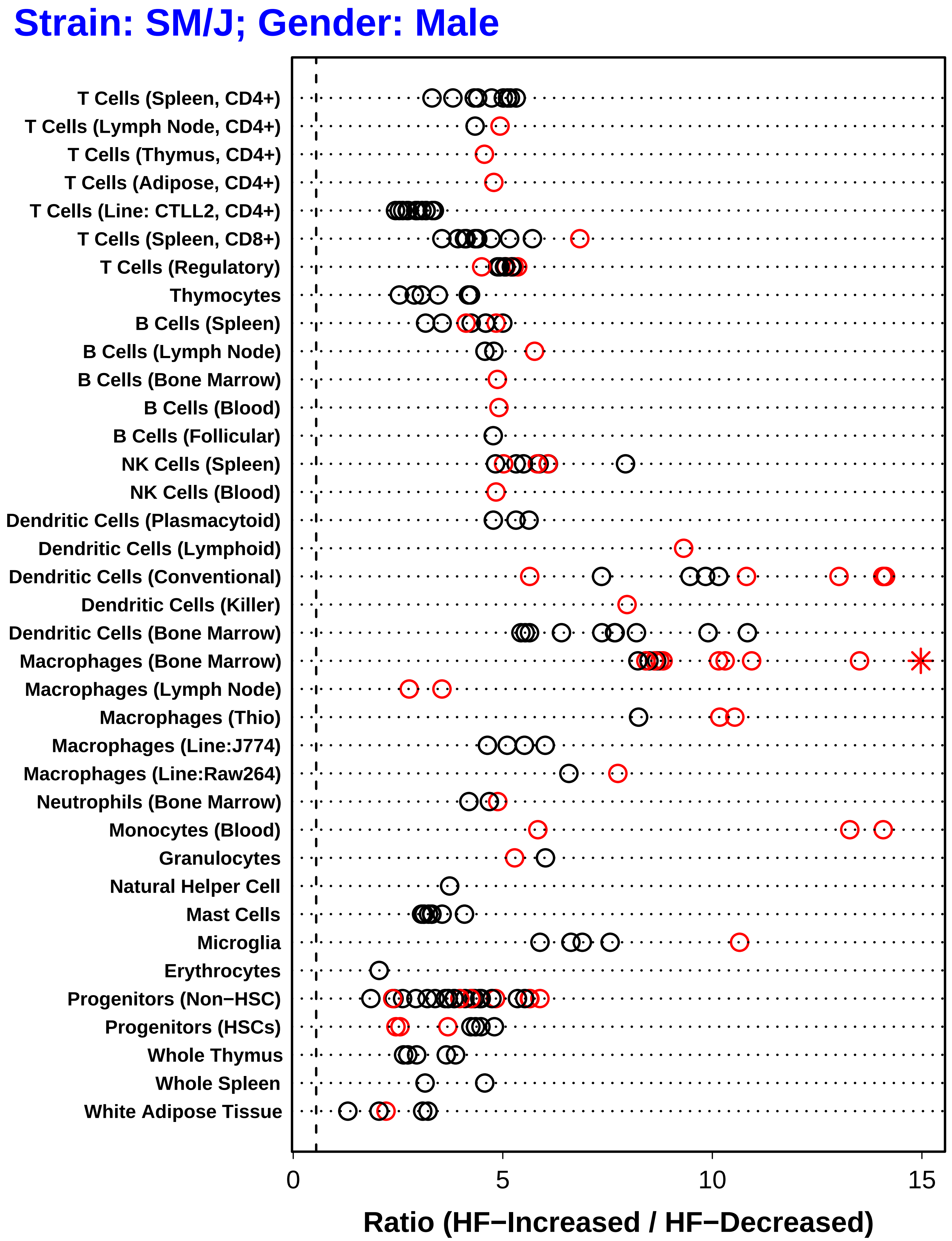

Strain: SM/J; Gender: Male
T Cells (Spleen, CD4+)
T Cells (Lymph Node, CD4+)
T Cells (Thymus, CD4+)
T Cells (Adipose, CD4+)
T Cells (Line: CTLL2, CD4+)
T Cells (Spleen, CD8+)
T Cells (Regulatory)
Thymocytes
B Cells (Spleen)
B Cells (Lymph Node)
B Cells (Bone Marrow)
B Cells (Blood)
B Cells (Follicular)
NK Cells (Spleen)
NK Cells (Blood)
Dendritic Cells (Plasmacytoid)
Dendritic Cells (Lymphoid)
Dendritic Cells (Conventional)
Dendritic Cells (Killer)
Dendritic Cells (Bone Marrow)
Macrophages (Bone Marrow)
Macrophages (Lymph Node)
Macrophages (Thio)
Macrophages (Line:J774)
Macrophages (Line:Raw264)
Neutrophils (Bone Marrow)
Monocytes (Blood)
Granulocytes
Natural Helper Cell
Mast Cells
Microglia
Erythrocytes
Progenitors (Non−HSC)
Progenitors (HSCs)
Whole Thymus
Whole Spleen
White Adipose Tissue
0
5
10
15
Ratio (HF−Increased / HF−Decreased)
