# Supplementary material for: Transcriptional Profiles of Leukocyte Populations Provide a Tool for Interpreting Gene Expression Patterns Associated with High Fat Diet in Mice
Source: PLoS One. 2010 Jul 29;5(7):e11861. doi: 10.1371/journal.pone.0011861 (PMC2912331; doi:10.1371/journal.pone.0011861)
Supplement: Table S1 — Data samples used to construct reference database for liver tissue (i.e., Treatment A from Figure 2). The procedure described in Figure 2 indicates that nA arrays were used as a reference set to identify signature transcripts associated with a given cell population. A total of nA = 56 arrays were obtained from Gene Expression Omnibus and assigned to this reference set treatment. These 56 hybridizations are listed below. As indicated in the table, each sample corresponds to a hybridization that involves RNA extracted from liver of young male or female mice (≤16 weeks of age). (0.04 MB PDF) [file pone.0011861.s007.pdf]

Table S1. Data samples used to construct reference database for liver tissue (i.e., Treatment *A* from Figure 2). The procedure described in Figure 2 indicates that n_A arrays were used as a reference set to identify signature transcripts associated with a given cell population. A total of n_A = 56 arrays were obtained from Gene Expression Omnibus and assigned to this reference set treatment. These 56 hybridizations are listed below. As indicated in the table, each sample corresponds to a hybridization that involves RNA extracted from liver of young male or female mice (≤ 16 weeks of age).

Series	Samples	Age	Strain	Gender
GSE10246	GSM258687<br>GSM258688	9 weeks	C57BL6	Male
GSE10744	GSM271266<br>GSM271269<br>GSM271278	12 weeks	C3HEBFEJ	Male
GSE10744	GSM271279<br>GSM271267<br>GSM271272	12 weeks	C57BL6J	Male
GSE10744	GSM271275<br>GSM271268<br>GSM271280	12 weeks	AKRJ	Male
GSE10744	GSM271270<br>GSM271283<br>GSM271274	12 weeks	DBA2J	Male
GSE10744	GSM271277<br>GSM271281<br>GSM271271	12 weeks	AJ	Male
GSE10744	GSM271282<br>GSM271276<br>GSM271273	12 weeks	129S2	Male
GSE9954	GSM252074<br>GSM252075<br>GSM252076	11weeks	C57BL6	Male
GSE18293	GSM456394<br>GSM456395<br>GSM456396	5 weeks	Swiss Webster	Female
GSE9441	GSM239850<br>GSM239851<br>GSM239852	12 weeks	AKRJ	Male
GSE9441	GSM239856<br>GSM239857<br>GSM239858	12 weeks	C57BL6J	Male
GSE9441	GSM239862<br>GSM239863<br>GSM239864	12 weeks	DBA2J	Male
GSE14395	GSM359832<br>GSM359833	11 weeks	SV129	Male

	GSM359834			
GSE14395	GSM359838<br>GSM359839<br>GSM359840	11 weeks	SV129	Female
GSE7357	GSM177149<br>GSM177150<br>GSM177151	7 weeks	C57BL6	Male
GSE7357	GSM177155<br>GSM177156<br>GSM177157	7 weeks	DBA2	Male
GSE8292	GSM205766<br>GSM205767<br>GSM205768	16 weeks	129S1SVIMJ	Male
GSE12693	GSM318346<br>GSM318643<br>GSM318644	10 weeks	BALBC	Male
GSE5959	GSM138289<br>GSM138290<br>GSM138291	8 weeks	C57BL6J	Female
