# Supplementary material for: Transcriptional Profiles of Leukocyte Populations Provide a Tool for Interpreting Gene Expression Patterns Associated with High Fat Diet in Mice
Source: PLoS One. 2010 Jul 29;5(7):e11861. doi: 10.1371/journal.pone.0011861 (PMC2912331; doi:10.1371/journal.pone.0011861)
Supplement: Table S2 — Data samples used to identify signature transcripts for leukocyte populations (i.e., treatment B in Figure 2). In total, N = 200 cell populations were evaluated, and the procedure carried out for the jth leukocyte population is described in Figure 2, where j = 1, …, 200. This table lists all 200 cell populations for which the procedure was applied. Most cell populations represent leukocytes, although some correspond to progenitor cells, thymus, spleen, or white adipose tissue. For each population, the samples listed correspond to replicate CEL files obtained from Gene Expression Omnibus. These samples were used as the nB biological replicates in Treatment B (see Figure 2) in order to identify signature transcripts for each population. (0.10 MB PDF) [file pone.0011861.s008.pdf]

Table S2. Data samples used to identify signature transcripts for leukocyte populations (i.e., treatment *B* in Figure 2). In total, *N* = 200 cell populations were evaluated, and the procedure carried out for the *j*th leukocyte population is described in Figure 2, where j = 1, ..., 200. This table lists all 200 cell populations for which the procedure was applied. Most cell populations represent leukocytes, although some correspond to progenitor cells, thymus, spleen, or white adipose tissue. For each population, the samples listed correspond to replicate CEL files obtained from Gene Expression Omnibus. These samples were used as the n_B biological replicates in Treatment *B* (see Figure 2) in order to identify signature transcripts for each population.

Population	Series	Samples	Description
1	GSE8836	GSM219522<br>GSM219523<br>GSM219524<br>GSM219525<br>GSM219535<br>GSM219539	CD4+ T cells (spleen)
2	GSE18669	GSM463721<br>GSM463722	CD4+ T cells (spleen)
3	GSE10813	GSM272999<br>GSM273003<br>GSM273004	CD4+ T cells (spleen)
4	GSE13306	GSM335922<br>GSM335923	CD4+ FOXP3- T cells, DC-activated (spleen)
5	GSE13306	GSM335929<br>GSM335930	CD4+ FOXP3- T cells, DC-activated (spleen)
6	GSE6506	GSM149583<br>GSM149584	CD4+ Naive T cells (spleen)
7	GSE6506	GSM149587<br>GSM149588	CD4+ Memory T cells (spleen)
8	GSE13306	GSM335933<br>GSM335934<br>GSM335935	CD4+ Memory T cells (spleen)
9	GSE13306	GSM335936<br>GSM335937<br>GSM335939	CD4+ Memory T cells (spleen)
10	GSE5245	GSM118665<br>GSM118666<br>GSM118667	CD4+ T cells (lymph node)
11	GSE7852	GSM176899<br>GSM180542<br>GSM180544	CD4+ T cells (lymph node)
12	GSE7852	GSM176893<br>GSM180533<br>GSM180536	CD4+ T cells (thymus)
13	GSE7852	GSM190563<br>GSM190564	CD4+ T cells (fat)

		GSM190565	
14	GSE10246	GSM258773<br>GSM258774	CD4+ T cells
15	GSE6259	GSM144164<br>GSM144165<br>GSM144166	CD4+ T cells
16	GSE6085	GSM108334<br>GSM108335<br>GSM108336<br>GSM108337<br>GSM108338	CD4+ T cells, line CTLL2
17	GSE6085	GSM108339<br>GSM108340<br>GSM108341<br>GSM108342	CD4+ T cells, line CTLL2, treated with IL-2 for 4 hours
18	GSE6085	GSM140923<br>GSM140924<br>GSM140925	CD4+ T cells, line CTLL2, treated with IL-2 for 30 minutes
19	GSE6085	GSM140926<br>GSM140927<br>GSM140928	CD4+ T cells, line CTLL2, treated with IL-2 for 1 hour
20	GSE6085	GSM140929<br>GSM140930<br>GSM140931	CD4+ T cells, line CTLL2, treated with IL-2 for 2 hours
21	GSE6085	GSM140932<br>GSM140933<br>GSM140934	CD4+ T cells, line CTLL2, treated with IL-2 for 6 hours
22	GSE6085	GSM140935<br>GSM140936<br>GSM140937	CD4+ T cells, line CTLL2, treated with IL-2 for 8 hours
23	GSE6085	GSM140938<br>GSM140939<br>GSM140940	CD4+ T cells, line CTLL2, treated with IL-2 for 10 hours
24	GSE6085	GSM140941<br>GSM140942<br>GSM140943	CD4+ T cells, line CTLL2, treated with IL-2 for 12 hours
25	GSE6085	GSM140944<br>GSM140945<br>GSM140946	CD4+ T cells, line CTLL2, treated with IL-2 for 16 hours
26	GSE6085	GSM140947<br>GSM140948<br>GSM140949	CD4+ T cells, line CTLL2, treated with IL-2 for 24 hours
27	GSE6085	GSM183455<br>GSM183456<br>GSM183457<br>GSM183458	CD4+ T cells, line CTLL2

28	GSE6085	GSM183459<br>GSM183460<br>GSM183461	CD4+ T cells, line CTLL2
29	GSE10246	GSM258775<br>GSM258776	CD8+ T cells
30	GSE6259	GSM144167<br>GSM144168<br>GSM144169	CD8+ T cells
31	GSE10813	GSM273000<br>GSM273001	CD8+ Naive T cells (spleen)
32	GSE6506	GSM149585<br>GSM149586	CD8+ Naive T cells (spleen)
33	GSE19825	GSM495040<br>GSM495041<br>GSM495042	CD8+ Naive T cells (spleen)
34	GSE10813	GSM273005<br>GSM273006	CD8+ Memory T cells (spleen)
35	GSE6506	GSM149589<br>GSM149590	CD8+ Memory T cells (spleen)
36	GSE9810	GSM247598<br>GSM247599	CD8+ T cells (spleen)
37	GSE8836	GSM219550<br>GSM219551<br>GSM219552<br>GSM219553<br>GSM219563<br>GSM219567	CD8+ T cells (spleen)
38	GSE19825	GSM495043<br>GSM495044<br>GSM495045<br>GSM495046	CD8+ $CD25^{lo}$ T cells (spleen)
39	GSE19825	GSM495047<br>GSM495048<br>GSM495049<br>GSM495050	CD8+ $CD25^{hi}$ T cells (spleen)
40	GSE10813	GSM272995<br>GSM273002<br>GSM272996<br>GSM272997<br>GSM272998	CD8+ T cells, NKT-like (spleen)
41	GSE6681	GSM154369<br>GSM154373	FoxP3+ YFP+ regulatory T cells
42	GSE10246	GSM258777<br>GSM258778	FoxP3+ regulatory T cells
43	GSE19512	GSM486563<br>GSM486564	FoxP3+ regulatory T cells

		GSM486565	
44	GSE19512	GSM486566<br>GSM486567<br>GSM486568	FoxP3+ regulatory T cells
45	GSE13306	GSM335931<br>GSM335932	FoxP3+ regulatory T cells (spleen)
46	GSE13306	GSM335924<br>GSM335925	FoxP3+ regulatory T cells, DC-activated (spleen)
47	GSE13306	GSM335927<br>GSM335928	FoxP3+ regulatory T cells (spleen)
48	GSE7852	GSM176897<br>GSM180538<br>GSM180540	CD4+ regulatory T cells (lymph node)
49	GSE7852	GSM176892<br>GSM180519<br>GSM180531	CD4+ regulatory T cells (thymus)
50	GSE7852	GSM190555<br>GSM190556<br>GSM190562	CD4+ regulatory T cells (fat)
51	GSE10246	GSM258783<br>GSM258784	CD4+ thymocytes
52	GSE10246	GSM258785<br>GSM258786	CD8+ thymocytes
53	GSE10246	GSM258781<br>GSM258782	CD4+ CD8+ thymocytes
54	GSE19528	GSM486995<br>GSM486996<br>GSM486997<br>GSM486998	CD4+ CD8+ thymocytes
55	GSE18281	GSM456501<br>GSM456502<br>GSM456503<br>GSM456504<br>GSM456505	CD3- CD45+ cortical thymocytes (CD90+ or CD117+)
56	GSE18281	GSM456506<br>GSM456507<br>GSM456508<br>GSM456510<br>GSM456511	$CD3^{hi}$ CD45+ medullary thymocytes
57	GSE10246	GSM258621<br>GSM258622	Marginal zone B cells (spleen)
58	GSE9810	GSM247595<br>GSM247596<br>GSM247597	B cells (spleen)
59	GSE6506	GSM149591<br>GSM149592	B cells (spleen)

60	GSE6259	GSM144161<br>GSM144162<br>GSM144163	B cells (spleen)
61	GSE4142	GSM94741<br>GSM94744<br>GSM94745	Naive B cells (spleen)
62		GSM94747<br>GSM94762<br>GSM94763<br>GSM94764	Plasma B cells (spleen)
63	GSE4142	GSM94765<br>GSM94766<br>GSM94767	Germinal center B cells (spleen)
64	GSE4142	GSM94768<br>GSM94769<br>GSM94771<br>GSM94772	Memory B cells (spleen)
65	GSE3203	GSM71992<br>GSM71993<br>GSM71994<br>GSM71995	B cells (lymph node)
66	GSE3203	GSM71996<br>GSM71997<br>GSM71998<br>GSM71999	B cells from influenza-infected mice (lymph node)
67	GSE3203	GSM72004<br>GSM72005<br>GSM72006<br>GSM72007	IFN-stimulated B cells (spleen)
68	GSE15808	GSM459907<br>GSM459908<br>GSM459909<br>GSM459910	B cells (bone marrow)
69	GSE10871	GSM275576<br>GSM275577	Memory B cells (blood)
70	GSE10246	GSM258663<br>GSM258664	Follicular B cells
71	GSE7764	GSM188001<br>GSM188003<br>GSM188004	Resting NK cells (spleen)
72	GSE9735	GSM245980<br>GSM245981<br>GSM245982	NK cells (spleen)
73	GSE11918	GSM301246<br>GSM301247	NK cells (spleen)
74	GSE9810	GSM247593	NK cells (spleen)

		GSM247594	
75	GSE13229	GSM334150<br>GSM334151	CD11B- NK cells (spleen)
76	GSE13229	GSM334152<br>GSM334153	CD11B+ CD27+ NK cells (spleen)
77	GSE13229	GSM334154<br>GSM334155	CD11B+ CD27- NK cells (spleen)
78	GSE9735	GSM245974<br>GSM245975<br>GSM245976	IL-12 and IL-18 treated NK cells (spleen)
79	GSE9735	GSM245977<br>GSM245978<br>GSM245979	IL-12 and IL-18 treated NK cells, infected (spleen)
80	GSE7764	GSM188002<br>GSM188005<br>GSM188006	NK cells stimulated with IL-15 for 24 hours (spleen)
81	GSE6506	GSM149581<br>GSM149582	NK cells (blood)
82	GSE10246	GSM258731<br>GSM258732	NK cells
83	GSE10246	GSM258649<br>GSM258650	B220+ Plasmacytoid Dendritic Cells
84	GSE9810	GSM247591<br>GSM247592	Plasmacytoid Dendritic Cells (spleen)
85	GSE12505	GSM314209<br>GSM314210	Plasmacytoid Dendritic Cells (spleen)
86	GSE10246	GSM258645<br>GSM258646	CD8A+ lymphoid Dendritic Cells
87	GSE11918	GSM301244<br>GSM301245	Conventional Dendritic Cells (spleen)
88	GSE9810	GSM247587<br>GSM247588	CD8alpha+ Conventional Dendritic Cells (spleen)
89	GSE9810	GSM247589<br>GSM247590	CD11B+ Conventional Dendritic Cells (spleen)
90	GSE12392	GSM310386<br>GSM310390	CD8alpha- Conventional Dendritic Cells (spleen)
91	GSE12392	GSM310388<br>GSM310391	CD8alpha+ Conventional Dendritic Cells (spleen)
92	GSE7219	GSM173580<br>GSM173581<br>GSM173582	Conventional Dendritic Cells (spleen)
93	GSE7219	GSM173588<br>GSM173590<br>GSM173591	Conventional Dendritic Cells treated with LPS and AntiCD40 agonist (spleen)
94	GSE6259	GSM144152<br>GSM144153	33D1+ Conventional Dendritic Cells (spleen)

		GSM144154	
95	GSE6259	GSM144158<br>GSM144159<br>GSM144160	DEC205+ Conventional Dendritic Cells (spleen)
96	GSE10246	GSM258647<br>GSM258648	CD8A- Conventional Dendritic Cells
97	GSE11918	GSM301248<br>GSM301249	Interferon producing killer dendritic cells (spleen)
98	GSE18460	GSM459835<br>GSM459836<br>GSM459837	Dendritic cells treated with *Lacto bacilli* for 4 hours (bone marrow)
99	GSE18460	GSM459838<br>GSM459839<br>GSM459840	Dendritic cells treated with *Lacto bacilli* for 10 hours (bone marrow)
100	GSE18460	GSM459841<br>GSM459842<br>GSM459843	Dendritic cells treated with *Lacto bacilli* for 18 hours (bone marrow)
101	GSE18460	GSM459844<br>GSM459845<br>GSM459846	Dendritic cells (bone marrow)
102	GSE18460	GSM459847<br>GSM459848	Dendritic cells (bone marrow)
103	GSE18460	GSM459849<br>GSM459850	Dendritic cells (bone marrow)
104	GSE20302	GSM508907<br>GSM508908<br>GSM508909	Dendritic cells (bone marrow)
105	GSE20302	GSM508910<br>GSM508911<br>GSM508912	Dendritic Cells treated with *lactobacillus* (bone marrow)
106	GSE20302	GSM508913<br>GSM508914<br>GSM508915	Dendritic Cells treated with Bifidobacterium (bone marrow)
107	GSE20302	GSM508916<br>GSM508917<br>GSM508918	Dendritic Cells treated with Bifidobacterium and Lactobacillus (bone marrow)
108	GSE15610	GSM390796<br>GSM390797<br>GSM390798	Macrophages (bone marrow)
109	GSE10246	GSM258693<br>GSM258694	Macrophages (bone marrow)
110	GSE15610	GSM390802<br>GSM390803<br>GSM390804	Macrophages treated with LPS for 4 hours (bone marrow)
111	GSE10246	GSM258695<br>GSM258696	Macrophages treated with LPS for 24 hours (bone marrow)

112	GSE10246	GSM258697<br>GSM258698	Macrophages treated with LPS for 2 hours (bone marrow)
113	GSE10246	GSM258699<br>GSM258700	Macrophages treated with LPS for 6 hours (bone marrow)
114	GSE19374	GSM480874<br>GSM480875<br>GSM480876	Macrophages (bone marrow)
115	GSE19374	GSM480877<br>GSM480878	Infected Macrophages (bone marrow)
116	GSE18500	GSM516564<br>GSM516567<br>GSM516578	Macrophages (bone marrow)
117	GSE18500	GSM516565<br>GSM516568	LM-infected Macrophages (bone marrow)
118	GSE18500	GSM516566<br>GSM516569	ST-infected Macrophages (bone marrow)
119	GSE18500	GSM516570<br>GSM516579	VSV-infected Macrophages (bone marrow)
120	GSE15767	GSM395738<br>GSM395739<br>GSM395740	Macrophages (lymph node)
121	GSE15767	GSM395741<br>GSM395742<br>GSM395743	Macrophages (lymph node)
122	GSE10246	GSM258701<br>GSM258702	Thioglycollate-elicited peritoneal macrophages
123	GSE10246	GSM258703<br>GSM258704	Thioglycollate-elicited peritoneal macrophages, treated with LPS for 1 hour
124	GSE10246	GSM258705<br>GSM258706	Thioglycollate-elicited peritoneal macrophages, treated with LPS for 7 hours
125	GSE4288	GSM237902<br>GSM237903<br>GSM237904	Macrophages, Line J774
126	GSE4288	GSM237905<br>GSM237906<br>GSM237907	Macrophages, Line J774, treated with LPS for 1 hour
127	GSE4288	GSM237908<br>GSM237909<br>GSM237910	Macrophages, Line J774, treated with LPS for 2 hours
128	GSE4288	GSM237911<br>GSM237912<br>GSM237913	Macrophages, Line J774, treated with LPS for 4 hours
129	GSE10246	GSM258755<br>GSM258756	Macrophages, Line RAW264

130	GSE12518	GSM314244<br>GSM314245<br>GSM314246	Macrophages, Line RAW264
131	GSE13693	GSM344315<br>GSM344316<br>GSM344318	Neutrophils (bone marrow)
132	GSE11973	GSM303257<br>GSM303268<br>GSM303276	Neutrophils (bone marrow)
133	GSE12001	GSM303489<br>GSM303490<br>GSM303491	Neutrophils (bone marrow)
134	GSE6506	GSM149593<br>GSM149594	Monocytes (blood)
135	GSE17256	GSM432167<br>GSM432168<br>GSM432169<br>GSM432170	Ly6C^{hi} Monocytes (blood)
136	GSE17256	GSM432171<br>GSM432172<br>GSM432173<br>GSM432174	Ly6C^{lo} Monocytes (blood)
137	GSE6506	GSM149595<br>GSM149596	Granulocytes (blood)
138	GSE10246	GSM258667<br>GSM258668	Mac1+ Gr1+ Granulocytes
139	GSE18752	GSM465593<br>GSM465594	Natural helper cells (mesentery)
140	GSE10246	GSM258711<br>GSM258712	Mast cells
141	GSE10246	GSM258713<br>GSM258714	Mast cells
142	GSE10246	GSM258715<br>GSM258716	Mast cells, treated with IgE+ antigen for 1 hour
143	GSE10246	GSM258717<br>GSM258718	Mast cells, treated with IgE+ antigen for 6 hours
144	GSE18500	GSM461107<br>GSM516561<br>GSM516571<br>GSM516580	Mast cells
145	GSE18500	GSM461108<br>GSM516562<br>GSM516572	Mast cells, LM-infected
146	GSE18500	GSM461109<br>GSM516563<br>GSM516573	Mast cells, ST-infected

147	GSE18500	GSM461110<br>GSM516574<br>GSM516581	Mast cells, VSV-infected
148	GSE18740	GSM465440<br>GSM465441<br>GSM465442	Microglia, Line BV2
149	GSE18740	GSM465443<br>GSM465444<br>GSM465445	Microglia, Line BV2, luteolin-treated
150	GSE18740	GSM465446<br>GSM465447<br>GSM465448	Microglia, Line BV2, LPS-treated
151	GSE18740	GSM465449<br>GSM465450<br>GSM465451	Microglia, Line BV2, treated with LPS and luteolin
152	GSE10246	GSM258721<br>GSM258722	Microglia
153	GSE6506	GSM149597<br>GSM149598	Nucleated Erythrocytes (bone marrow)
154	GSE6623	GSM153717<br>GSM153718<br>GSM153719	Myeloid progenitor cells (bone marrow)
155	GSE17765	GSM443766<br>GSM443767<br>GSM443768	Myeloid progenitor cells (bone marrow)
156	GSE19142	GSM474589<br>GSM474590	Common lymphoid progenitors , $RAG1^{high}$ (bone marrow)
157	GSE19142	GSM474591<br>GSM474592	Common lymphoid progenitors, $RAG1^{low}$ (bone marrow)
158	GSE14833	GSM371216<br>GSM371217	Common lymphoid progenitors (bone marrow)
159	GSE18669	GSM463714<br>GSM463715<br>GSM463716	Multipotent progenitors (bone marrow)
160	GSE10246	GSM258719<br>GSM258720	Mega-Erythrocytes
161	GSE10246	GSM258665<br>GSM258666	Granulocyte-monocyte progenitors (bone marrow)
162	GSE14833	GSM371221<br>GSM371222	Granulocyte-monocyte progenitors (bone marrow)
163	GSE14833	GSM371223<br>GSM371224<br>GSM371225	Granulocyte-monocyte progenitors (bone marrow)
164	GSE17765	GSM443769<br>GSM443770	B cell progenitors (bone marrow)
165	GSE15808	GSM459882	B cell progenitors (bone marrow)

		GSM459883	
166	GSE8726	GSM216494<br>GSM216495<br>GSM216496<br>GSM216497	Erythroblasts (bone marrow)
167	GSE14833	GSM371253<br>GSM371254<br>GSM371255	Proerythroblasts (bone marrow)
168	GSE18669	GSM463717<br>GSM463718<br>GSM463719<br>GSM463720	Megakaryocyte-Erythrocytes (bone marrow)
169	GSE14833	GSM371235<br>GSM371236<br>GSM371237<br>GSM371238	Megakaryocyte-Erythrocytes (bone marrow)
170	GSE13693	GSM344320<br>GSM344321<br>GSM344322	CD117+ Gr1+ MAC1- Myeloblasts (bone marrow)
171	GSE13693	GSM344325<br>GSM344326<br>GSM344328	CD117+ Gr1+ MAC1+ Myeloblasts (bone marrow)
172	GSE14833	GSM371248<br>GSM371249<br>GSM371250	Pre-CFUE (bone marrow)
173	GSE14833	GSM371213<br>GSM371214<br>GSM371215	CFUE (bone marrow)
174	GSE14833	GSM371211<br>GSM371212	CD4+ progenitors (bone marrow)
175	GSE14833	GSM371218<br>GSM371219<br>GSM371220	Early T lineage progenitors (bone marrow)
176	GSE14833	GSM371226<br>GSM371227	IgM+ progenitors (bone marrow)
177	GSE14833	GSM371228<br>GSM371229<br>GSM371230<br>GSM371231<br>GSM371232	LMPP progenitors (bone marrow)
178	GSE14833	GSM371239<br>GSM371240<br>GSM371241	MkP progenitors (bone marrow)
179	GSE14833	GSM371242<br>GSM371243	Mature NK cells (bone marrow)
180	GSE14833	GSM371244	Pre-B cells (bone marrow)

		GSM371245<br>GSM371246<br>GSM371247	
181	GSE14833	GSM371251<br>GSM371252	Pro-B cells (bone marrow)
182	GSE14833	GSM371256<br>GSM371257	Hematopoietic stem cells, short term (bone marrow)
183	GSE14833	GSM371233<br>GSM371234	Hematopoietic stem cells, long term (bone marrow)
184	GSE6506	GSM149579<br>GSM149580	Hematopoietic stem cells, long term (bone marrow)
185	GSE6623	GSM153707<br>GSM153709<br>GSM153710	Hematopoietic stem cells, LSK (bone marrow)
186	GSE20352	GSM509837<br>GSM509838<br>GSM509839	Hematopoietic stem cells, LSK (bone marrow)
187	GSE18669	GSM463711<br>GSM463712<br>GSM463713	Hematopoietic stem cells (bone marrow)
188	GSE17765	GSM443761<br>GSM443762	Hematopoietic stem cells (bone marrow)
189	GSE18281	GSM456512<br>GSM456514<br>GSM456515	Subscapular cortical region of thymus
190	GSE18281	GSM456517<br>GSM456518<br>GSM456519	Central cortical region of thymus
191	GSE18281	GSM456521<br>GSM456522<br>GSM456523	Perimedullary cortical region of thymus
192	GSE18281	GSM456524<br>GSM456525<br>GSM456526<br>GSM456527<br>GSM456528<br>GSM456529<br>GSM456530	Thymus, whole cortex
193	GSE18281	GSM456531<br>GSM456532<br>GSM456533<br>GSM456534<br>GSM456535<br>GSM456536<br>GSM456537	Thymus, whole medulla
194	GSE7020	GSM162086	Spleen

		GSM162087<br>GSM162088<br>GSM162089	
195	GSE9287	GSM235399<br>GSM236566<br>GSM236567<br>GSM236573	Spleen
196	GSE13432	GSM338983<br>GSM338984<br>GSM338985	White Adipose Tissue
197	GSE13432	GSM338989<br>GSM338990<br>GSM338991	White Adipose Tissue
198	GSE8679	GSM215234<br>GSM215235	White Adipose Tissue
199	GSE8044	GSM198456<br>GSM198457<br>GSM198458	White Adipose Tissue
200	GSE9132	GSM231019<br>GSM231020<br>GSM231021	White Adipose Tissue
